# D-EE: Distributed software for visualizing intrinsic structure of large-scale single-cell data

**DOI:** 10.1093/gigascience/giaa126

**Published:** 2020-11-11

**Authors:** Shaokun An, Jizu Huang, Lin Wan

**Affiliations:** NCMIS, LSEC, LSC, Academy of Mathematics and Systems Science, Chinese Academy of Sciences, Zhongguancun East Road, Haidian District, Beijing, 100190, China; School of Mathematical Sciences, University of Chinese Academy of Sciences, Yuquan Road, Shijingshan District, Beijing, 100049, China; NCMIS, LSEC, LSC, Academy of Mathematics and Systems Science, Chinese Academy of Sciences, Zhongguancun East Road, Haidian District, Beijing, 100190, China; School of Mathematical Sciences, University of Chinese Academy of Sciences, Yuquan Road, Shijingshan District, Beijing, 100049, China; NCMIS, LSEC, LSC, Academy of Mathematics and Systems Science, Chinese Academy of Sciences, Zhongguancun East Road, Haidian District, Beijing, 100190, China; School of Mathematical Sciences, University of Chinese Academy of Sciences, Yuquan Road, Shijingshan District, Beijing, 100049, China

**Keywords:** dimensionality reduction, distributed storage, distributed computation, large-scale data, single-cell sequencing

## Abstract

**Background:**

Dimensionality reduction and visualization play vital roles in single-cell RNA sequencing (scRNA-seq) data analysis. While they have been extensively studied, state-of-the-art dimensionality reduction algorithms are often unable to preserve the global structures underlying data. Elastic embedding (EE), a nonlinear dimensionality reduction method, has shown promise in revealing low-dimensional intrinsic local and global data structure. However, the current implementation of the EE algorithm lacks scalability to large-scale scRNA-seq data.

**Results:**

We present a distributed optimization implementation of the EE algorithm, termed distributed elastic embedding (D-EE). D-EE reveals the low-dimensional intrinsic structures of data with accuracy equal to that of elastic embedding, and it is scalable to large-scale scRNA-seq data. It leverages distributed storage and distributed computation, achieving memory efficiency and high-performance computing simultaneously. In addition, an extended version of D-EE, termed distributed optimization implementation of time-series elastic embedding (D-TSEE), enables the user to visualize large-scale time-series scRNA-seq data by incorporating experimentally temporal information. Results with large-scale scRNA-seq data indicate that D-TSEE can uncover oscillatory gene expression patterns by using experimentally temporal information.

**Conclusions:**

D-EE is a distributed dimensionality reduction and visualization tool. Its distributed storage and distributed computation technique allow us to efficiently analyze large-scale single-cell data at the cost of constant time speedup. The source code for D-EE algorithm based on C and MPI tailored to a high-performance computing cluster is available at https://github.com/ShaokunAn/D-EE.

## Background

The advent of single-cell sequencing provides high-dimensional profiles of cellular states at single-cell resolutions (e.g., single-cell RNA sequencing [scRNA-seq] of transcriptomes), offering the opportunity to unveil intrinsic biological processes and mechanisms. Dimensionality reduction and visualization methods have been extensively studied because they play vital roles in revealing the intrinsic structures underlying scRNA-seq high-dimensional data [1]. Nonetheless, it is still challenging for these state-of-the-art methods of dimensionality reduction and visualization to preserve both local and global structures of data in low-dimensional space. For example, the celebrated *t*-distributed stochastic neighbor embedding (t-SNE) algorithm [2] is widely used in the single-cell community [3]. It emphasizes the preservation of local structures, but it often distorts global structures [4–6]. As a solution, the Uniform Manifold Approximation and Projection (UMAP) algorithm [7] was developed, with the aim to preserve global structures, drawing increasing attention in the single-cell data analysis community [8]. However, a recent study showed that UMAP does not improve upon t-SNE in this regard when using the same initialization [9], making the validity of UMAP debatable.

In contrast, elastic embedding (EE), a nonlinear dimensionality reduction method, attempts to preserve both local and global structures underlying the data [4]. To achieve this goal, EE penalizes the placement of latent points in close proximity away from dissimilar data points in high-dimensional space, thus resolving the difficulty of global structure preservation (see [4], or Methods for details). EE has attracted increasing interest among statistical researchers [10]. It has also shown remarkable performance on visualizing the intrinsic structures of scRNA-seq data [1,5,11]. However, the current implementations of the EE algorithm are not scalable to sample size *N* (e.g., number of cells). Thus, it cannot be used for large-scale scRNA-seq datasets. For example, the storage of the attractive and the repulsive weight matrices of the EE algorithm is $\mathcal {O}(N^2)$.

**Figure 1:**
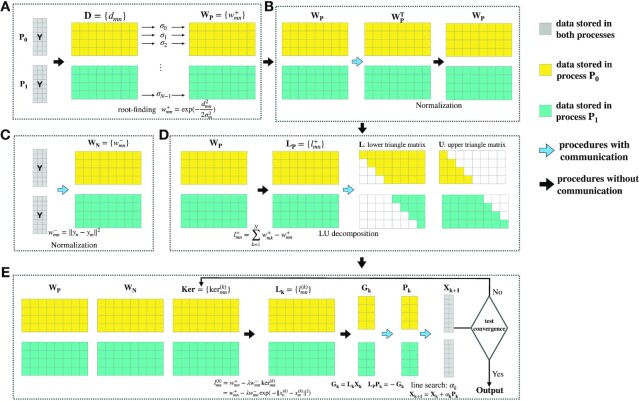
Overview of D-EE algorithm. The D-EE algorithm can be decomposed into 5 parts. Part A: the computation of matrices $\mathbf {D}$ and $\mathbf {W_P}$. To obtain $\mathbf {W_P}$, the parameters {σ_*n*_} are determined by solving a series of root-finding problems. Part B: the symmetry and normalization of the matrix $\mathbf {W_P}$. Part C: the computation and normalization of $\mathbf {W_N}$. Part D: the computation of the Laplacian matrix $\mathbf {L_P}$ together with LU decomposition. Part E: the computation of $\mathbf {X_{k+1}}$ by solving an optimization problem with the classic Quasi-Newton methods iteratively. In the *k*th iteration, $\mathbf {L_k}$ is computed on the basis of $\mathbf {W_P, W_N}$, and $\mathbf {X_k}$, which are used to obtain the gradient $\mathbf {G_k = L_kX_k}$. The descent direction $\mathbf {P_k}$ is then determined by solving a linear system $\mathbf {L_PP_k=-G_k}$. Finally, $\mathbf {X_{k+1}}$ is updated according to $\mathbf {X_{k+1}=X_k+}\alpha _k\mathbf {P_k}$, where the step size α_*k*_ is calculated by a line search method.

Therefore, we present a distributed optimization implementation of EE, termed D-EE. D-EE not only reveals the low-dimensional intrinsic structures of data with the same accuracy as EE but also is scalable to large-scale scRNA-seq data. It leverages distributed storage and distributed computation, achieving memory efficiency and high-performance computing simultaneously (Fig. 1). In addition, a distributed optimization implementation of the time-series EE (TSEE) algorithm [11], termed D-TSEE, is also provided for visualizing large-scale time-series scRNA-seq data. In this study, we demonstrate the power of D-EE and D-TSEE on both simulated and real datasets. Both D-EE and D-TSEE (1) achieve the same accuracy as EE and TSEE, respectively; and (2) gain high strong scaling performance on large-scale datasets.

## Methods

### Elastic embedding algorithm

EE was proposed by Carreira-Perpiñán [4]. It optimizes an energy function containing the attractive and repulsive terms.

Given *N* samples $\boldsymbol{Y} = \lbrace y_1, y_2, \dots , y_N\rbrace$, where $y_i \in \mathbb {R}^D$ represents its high-dimensional coordinates, the goal of EE is to map the data from high-dimensional space onto a low-dimensional representation $\mathbf {X}=\lbrace x_1, x_2, \dots , x_N\rbrace$ with $x_i\in \mathbb {R}^d$ and *d* ≪ *D* by minimizing an energy function

\begin{eqnarray*}
{
E(\mathbf {X}, \lambda )=\sum \nolimits _{m,n=1}^N w_{nm}^+\Vert x_n-x_m\Vert ^2+\lambda \sum \nolimits _{m,n=1}^N w_{nm}^- \exp (-\Vert x_n-x_m\Vert ^2), }
\end{eqnarray*}

where $w_{nm}^+=\exp {\left[-(1/2)\Vert y_n-y_m\Vert ^2/\sigma _n ^2\right]}$ and $w_{nm}^- = \Vert y_n-y_m\Vert ^2$. The first term acts as an attractive force to preserve local distances, while the second term acts as a repulsive force to preserve global structures or to separate latent points. The parameter $\lambda \in \mathbb {R}^+$ trades off the 2 terms, and a larger value implies that preservation of global structures is more important. In single-cell data analysis, with λ = 10, EE can achieve robust performance with high accuracy [5]. Therefore, we set the default value of λ as 10 for D-EE.

The σ_*m*_ in $w_{mn}^+$ is a sample-specific scaling parameter. It is estimated adaptively by solving a sample-specific root-finding problem, such that the sample-specific distribution over its neighbors has a desired perplexity (see [12] for details). We set a default value of perplexity as 20 in this study. It is worth noting that a newly proposed combinational perplexity has been applied to t-SNE [3], which greatly enhances the performance of t-SNE in preservation of global structures. The combinational perplexity can be also adopted by D-EE in a future update.

An extension of EE, TSEE [11], was recently proposed to handle the dimensionality reduction problems of time-series scRNA-seq data. It works by minimizing

\begin{equation*} \begin{aligned} E(\mathbf {X}, \lambda )=&\sum \nolimits _{m,n=1}^N w_{nm}^+\Vert x_n-x_m\Vert ^2\\ &+\lambda \sum \nolimits _{m,n=1}^N (w_{nm}^- +\beta t_{nm})\exp (-\Vert x_n-x_m\Vert ^2), \end{aligned} \end{equation*}

where *t_nm_* represents the dissimilarity of time of pairwise points, and β trades off the weights between dissimilarities of time stages and expression space.

## Numerical optimization of EE

Because the optimization solution of TSEE is basically the same as that of EE, we only give the numerical solution of EE. First, we denote $\mathbf {W_P}=\lbrace w_{nm}^+\rbrace$ and $\mathbf {W_N}=\lbrace w_{nm}^-\rbrace$. Owing to the existence of parameters {σ_*n*_}, $\mathbf {W_P}$ is not a symmetrical matrix but we make it symmetric by taking $\mathbf {W_P}:=\mathbf {W_P}+\mathbf {W_P}^T$. Next, the diagonal elements of $\mathbf {W_P}$ and $\mathbf {W_N}$ are set to zero. Finally, each element is normalized by being divided by the sum of all elements in the matrix.

To solve the optimization problem, the classic quasi-Newton methods update $\mathbf {X_{k+1}}$ according to $\mathbf {X_{k+1}=X_k+\alpha _k P_k}$ in the *k*th iteration, where $\mathbf {\alpha _k}$ is the step length determined by a line search procedure and $\mathbf {P_k}$ is the search direction obtained by solving the Jacobian system $\mathbf {B_kP_k=-G_k}$. In this equation, $\mathbf {B_k}$ is positive-definite to guarantee the decrease of the objective function. $\mathbf {G_k = L_kX_k}$ is the gradient of the objective function in the *k*th iteration, where $\mathbf {L_k}$ is the Laplacian of $\mathbf {W_k} = \lbrace w^{(k)}_{mn}\rbrace$ with $w_{mn}^{(k)} = w_{mn}^+-\lambda w_{mn}^- \exp {(-\Vert x_n^{(k)}-x_m^{(k)}\Vert ^2)}$. These procedures are repeated until a certain termination criterion is satisfied. During the iteration, $\mathbf {B_k}$ generally needs to be updated in each iteration as well.

To solve the EE-like optimization problems, a technique termed ''partial-Hessian optimization strategies" has been proposed to use partial information of the Hessian $\mathbf {L_P}$ [13], which is the Laplacian of $\mathbf {W_P}$ and is invariant in each iteration. This invariance makes it possible to utilize some precondition approaches, e.g., lower–upper (LU) decomposition, to improve calculation efficiency. The effectiveness of the determined direction, called "spectral direction", has been validated experimentally in previous work [13].

## D-EE algorithm

We provide a distributed optimization implementation of EE, termed D-EE. The overview of the newly proposed D-EE algorithm is given in Fig. 1. During whole optimization implementation, multiple processes are used for computation and storage of data. In Fig. 1, 2 processes, ${\cal P}_0$ and ${\cal P}_1$, are taken as an example. To achieve high performance in computing and memory efficiency simultaneously, our proposed distributed algorithm divides data ($\mathbf {W_P}$, $\mathbf {W_N}$, and $\mathbf {G_k}$) by rows for the multiple processes assigned. To avoid frequent communication, the whole original high-dimensional dataset $\mathbf {Y}$ is read and stored in each process, and the low-dimensional embedding $\mathbf {X}$ is established in each process as well because the storage consumed by $\mathbf {Y}$ and $\mathbf {X}$ is much less when compared to other *N* × *N* matrices used during computation. It is worth noting that, because most of the computation of each row in 1 matrix generally merely depends on the same row of other matrices (see the approximated computational complexity of D-EE in the following section for details), the partition procedure that we design in the D-EE algorithm is an almost optimal partition in parallel computing as a result of the optimal leverage of computation and communication. On the one hand, the total computational cost of the D-EE algorithm is almost the same as that of the centralized algorithm of EE. On the other hand, most procedures in the D-EE algorithm are communication-free, as indicated in Fig. 1 by the black arrows. Even though some procedures still exist with communication, as shown in Fig. 1 by blue arrows, the communication volume is on a much lower order than the cost of computation.

### Computation of matrices $\mathbf {D, W_P, W_N}$

As mentioned before, the matrices $\mathbf {W_P, W_N}$ depend on the high-dimensional data $\mathbf {Y}$ and $\lbrace \sigma _n\rbrace _{n=1}^N$. Because each σ_*n*_ is obtained by solving a root-finding problem from the *n*th row of the distance matrix $\mathbf {D}$, each matrix is equally, or almost equally, partitioned into multiple nonoverlapping parts by rows and stored in multiple processes, as shown in Fig. 1A. Let us denote $\mathbf {D}=[\mathbf {D}^1,\cdots ,\mathbf {D}^P]$, where submatrix $\mathbf {D}^i$ with size of *M_i_* × *N* is stored in the *i*th process and *P* is the number of processes we used. The [⋅⋅⋅] represents a column vector. Similar notations are used for the other *N* × *N* matrices. It is clear that each row of matrices $\mathbf {D, W_P, W_N}$ depends on all original high-dimensional data $\mathbf {Y}$. Therefore, we load a copy of $\mathbf {Y}$ into each process to avoid frequent communication.

In the centralized implementation of EE, the parameters σ_*n*_, *n* = 1, …, *N*, are calculated by iteratively solving a sequence of root-finding problems. The iteration method for the root-finding problems is improved by reordering the computation of $\lbrace \sigma _n\rbrace _{n=1}^N$ according to the distances of all samples ($\mathbf {Y}$), which is also the complete distance matrix $\mathbf {D}$ [12]. Then the reordered root-finding problems are sequentially solved by taking the solution of the previous one as the initial value of the next. Because the parameters are distributed in different processes, it is clear that the sequential root-finding approach cannot be parallelized without modifications. In the D-EE algorithm, we calculate $\lbrace \sigma _n\rbrace _{n=1}^N$ in the following parallel way. First, we decompose $\lbrace \sigma _n\rbrace _{n=1}^N$ into *P* subsets as $\Sigma _i=\lbrace \sigma _n\rbrace _{n={{\cal M}_i+1}}^{{\cal M}_{i+1}}$ with *i* = 0, ⋅⋅⋅, *P* − 1. The elements in the *i*th subset Σ_*i*_ are computed and stored in the *i*th process. Similar to the centralized algorithm of EE, we then reorder Σ_*i*_ according to the distance matrix $\mathbf {D}^i$ and iteratively solve the corresponding root-finding problems within the *i*th process. According to the distributions of the initial data $\mathbf {Y}$ and the matrices established before, we conclude that the D-EE algorithm calculates $\lbrace \sigma _n\rbrace _{n=1}^N$ in parallel, which is communication-free. The efficiency of the root-finding approach is also guaranteed by the local order. Because we only change the order and initial guesses of the root-finding problems, the solutions of the root-finding problems, as obtained from D-EE, are almost the same as those from EE. With the whole original high-dimensional dataset $\mathbf {Y}$ and the subset Σ_*i*_, we can compute the following submatrices $\mathbf {W_P}^i,\, \mathbf {W_N}^i$ in the *i*th process. Thus, we give a parallel and communication-free approach to compute matrices $\mathbf {D,\, W_P,\, W_N}$.

### Normalization of $\mathbf {W_P}$ and $\mathbf {W_N}$

After computing matrices $\mathbf {W_P},\, \mathbf {W_N}$, each process sets the diagonal elements belonging to it as 0 in parallel. Then, we set $\mathbf {W_P}:=\mathbf {W_P}+\mathbf {W_P}^T$ such that $\mathbf {W_P}$ becomes a symmetric matrix. Let us denote $\mathbf {W_P}^T:=\hat{\mathbf {W}}_{\mathbf {P}}=[\hat{\mathbf {W}}_{\mathbf {P}}^1, \cdots , \hat{\mathbf {W}}_{\mathbf {P}}^p]$, where submatrix $\hat{\mathbf {W}}_{\mathbf {P}}^i$ has the size of *M_i_* × *N*. In the *i*th process, we first obtain the elements of the submatrix $\hat{\mathbf {W}}_{\mathbf {P}}^i$ from the other *P* − 1 processes by communication and then compute $\mathbf {W_P}^i:=\mathbf {W_P}^i+\hat{\mathbf {W}}_{\mathbf {P}}^i$. Here point-to-point communication happens, and the communication volume for each process is ${\cal O}(N^2/P)$.

To normalize the matrices $\mathbf {W_P},\, \mathbf {W_N}$, each element should be divided by the sum of all elements in the matrix. The sum of all elements in matrix $\mathbf {W_P}$ is parallel computed as follows. First, each process calculates the sum of all elements in the submatrix $\mathbf {W_P}^i$ independently. We denote the sum of all elements in the submatrix $\mathbf {W_P}$ and $\mathbf {W_P}^i$ as ${\cal S}$ and ${\cal S}^i$, respectively. Then we compute the sum of all elements in matrix $\mathbf {W_P}$ by ${\cal S}=\sum _{i=1}^P{\cal S}_i$ through an MPI_Allgather action. Here all-to-all communication happens, and the communication volume for each process is ${\cal O}(P)$. Then, we normalize matrix $\mathbf {W_P}$ in each process by taking $\mathbf {W_P}^i=\mathbf {W_P}^i/{\cal S}$ in parallel without communication. The normalization of matrix $\mathbf {W_N}$ is done in a similar way.

### Computation of low-dimensional embedding $\mathbf {X}$

After normalizing $\mathbf {W_P}$, its Laplacian $\mathbf {L_P}$, which is needed for the subsequent determination of descent direction, is computed in parallel as follows. In the *i*th process, we calculate the elements of submatrix $\mathbf {L_P}^i$ by using $l_{mn}^+=\sum \limits _{k=1}^Nw_{mk}^+-w_{mn}^+$, where $l^+_{mn}$ and $w^+_{mn}$ are the elements of matrices $\mathbf {L_P}$ and $\mathbf {W_P}$, respectively. Because the 2 matrices are partitioned by row in the same way, the computation of $\mathbf {L_P}$ is also communication-free.

The low-dimensional embedding $\mathbf {X}$ is obtained by solving the optimization problem with the partial-Hessian optimization strategy. During the quasi-Newton procedures, the dense linear system $\mathbf {L_P}\mathbf {P_k}=-\mathbf {G_k}$ must be solved in parallel. In the D-EE algorithm, we perform LU decomposition on $\mathbf {L_P}$. Considering that $\mathbf {L_P}$ is positive semi-definite but not positive definite, a small value μ is added to the diagonal of $\mathbf {L_P}$ in practice. During the following sections, we still use $\mathbf {L_P}$ to denote the adjusted matrix. LU decomposition on $\mathbf {L_P}={\cal L}{\cal U}$ is done with PETSc, which provides uniform and efficient access to all linear system solvers in the package, including parallel and sequential, direct, and iterative [14–16]. Here, ${\cal L}$ and ${\cal U}$ are the corresponding lower and upper triangle matrices, respectively. With the decomposition of LU, the dense linear system $\mathbf {L_P}\mathbf {P_k}=-\mathbf {G_k}$ is replaced by 2 sublinear systems ${\cal L}\hat{\mathbf {P}}_\mathbf {k}=-\mathbf {G_k}$ and ${\cal U}\mathbf { P_k}=\hat{\mathbf {P}}_\mathbf {k}$, which can be solved by the backward substitution method.

As shown in Fig. 1D, the partitions of $\mathbf {L_P}$, ${\cal L}$, and ${\cal U}$ are the same as $\mathbf {W_P}$. Let us denote $\mathbf {P_k}=[\mathbf {P_k}^1,\cdots ,\mathbf {P_k}^P]$, where submatrix $\mathbf {P_k}^i$ with size of *M_i_* × *d* is stored in the *i*th process, and a similar partition is performed on $\mathbf {G_k}$. Based on the partitions of $\mathbf {L_P}$, ${\cal L}$, ${\cal U}$, $\mathbf {P_k}$, and $\mathbf {G_k}$, the computational complexities per process of LU decomposition and backward substitution are ${\cal O}(N^3/P)$ and ${\cal O}(N^2/P)$, with corresponding communication volumes of ${\cal O}(N^2/P)$ and ${\cal O}(N/P)$, respectively. According to the analysis, LU decomposition is only done in the first iteration of the quasi-Newton method, and matrices ${\cal L}$ and ${\cal U}$ are stored and reused during the whole quasi-Newton procedure.

The gradient $\mathbf {G_k}$ on the right-hand side of the linear system $\mathbf {L_P}\mathbf {P_k}=-\mathbf {G_k}$ is calculated by $\mathbf {G_k}=\mathbf {L_k}\mathbf {X_k}$, where the *N* × *N* matrix $\mathbf {L_k}$ depends on matrices $\mathbf {W_P, \, W_N,}$ and $\mathbf {Ker}$. Here the elements of matrix $\mathbf {Ker}$ are defined as $\ker _{mn} = \exp {(-\Vert x_m-x_n\Vert ^2)}$, and the elements of matrix $\mathbf {L_k}$ are defined as $l^{(k)}_{mn} = w_{mn}^+-\lambda w_{mn}^- \ker _{mn}^{(k)}$. As shown in Fig. 1E, the partitions of matrices $\mathbf {L_k}$ and $\mathbf {Ker}$ are the same as those of $\mathbf {W_P}$. In D-EE, we store all elements of $\mathbf {X_k}$ in each process, which is the same as the original high-dimensional data $\mathbf {Y}$. Thus, we can parallel-compute matrices $\mathbf {L_k}$ and $\mathbf {Ker}$ in the same way with the matrix $\mathbf {W_N}$, which means the procedure is also communication-free.

After solving the linear system, we obtain the search direction $\mathbf {P_k}$. Then, we update $\mathbf {X_{k+1}}$ according to $\mathbf {X_{k+1}=X_k +\alpha _kP_k}$, where α_*k*_ is determined by a line search approach. As mentioned before, the low-dimensional embedding $\mathbf {X_k}$ is stored sequentially, but $\mathbf {P_k}$ is distributed stored. Thus, we first compute the elements of submatrix $\mathbf {P_{k+1}}^i$ in the *i*th process and then gather all elements of $\mathbf {P_{k}}$ in each process by the all-gather function in MPI. Here all-to-all communication happens, and the order of communication volume for each process is ${\cal O} (Nd )$. In line search steps, we need to calculate the energy function $E(\mathbf {X}, \lambda )$ several times, which is computed in parallel according to the following formula:

\begin{equation*} \begin{aligned} E(\mathbf {X}, \lambda )=&\sum \nolimits _{i=1}^P\Bigg \{\sum \nolimits _{{m={\cal M}_i+1}}^{{{\cal M}_{i+1} }}\sum \nolimits_{n=1}^N \Big [ w_{mn}^+\Vert x_n-x_m\Vert ^2\\ &+\lambda w_{mn}^- \exp (-\Vert x_n-x_m\Vert ^2)\Big ] \Bigg \}. \end{aligned} \end{equation*}

The summation included in the curly braces is calculated in each process simultaneously and then gathered by the MPI all-gather function. Here all-to-all communication happens, and the communication volume for each process is ${\cal O} (P )$.

## Results

### Data description

We test the accuracy and scalability of D-EE on 3 datasets. The first simulated dataset [17], named PHATE data for convenience, consists of 1,440 samples and 60 features. It is a complex tree structure that simulates a cellular developmental process, namely, progressions, branch or split in progressions, and end state of progression, composed of 10 branches in total. We first perform principal component analysis (PCA) on the original data, reserving a 1,440 samples × 7 features matrix.

The second dataset characterizes the process of mouse hematopoietic stem and progenitor cells (HSPC) bifurcating to myeloid and erythroid precursors [18], consisting of 4,423 samples. The obtained single-cell read count dataset is pre-processed by the Seurat package (version 3.2.1) [19,20]. First, we normalize the gene expression in each sample as follows: we divide each gene read count by the total read counts for each cell and then multiply by a scale factor of 10^4^ and add 1, followed by taking a logarithmic transformation. Second, we select the top 2,000 variable genes using the default “vst” method of the Seurat package, i.e., variance-stabilizing transformation [21]. Finally, we conduct PCA on the processed data and select the top 50 largest principal components, resulting in a 4,423 samples × 50 features matrix as input of EE and D-EE.

The third dataset is a large-scale time-series scRNA-seq dataset containing ~250,000 cells [22]. The data characterize reprogramming of fibroblasts to induced pluripotent stem cells (iPSC), which were collected at half-day intervals across 18 days, resulting in 39 time points. Because the final time point of the iPSC status was not annotated temporally, we therefore set the final point as 20th day for the input to D-TSEE. We pre-process these data with the Seurat package as well. Same as the pre-processing of the HSPC data, we first filter cells and genes to include cells where ≥200 features are detected and to include genes detected in ≥50 cells, obtaining 259,081 cells and 19,427 genes. After that, we perform logarithmic transformation, select variable features, and perform PCA as described in the HSPC dataset, obtaining a 259,081 samples × 50 features matrix as input for the dimensionality reduction methods.

### D-EE achieves high strong scaling efficiency

We evaluate D-EE using both simulated and real scRNA-seq datasets. The numerical tests are carried out on the LSSC-IV supercomputer. The 400 computing nodes of LSSC-IV comprise 2 18-core Intel Xeon Gold CPUs with 192 GB local memory and are interconnected via a proprietary high-performance network. First, we use PHATE data and HSPC data to test the consistency between D-EE and EE results. We use 36 processes in both D-EE algorithms. The low dimensions are set to 2 for the convenience of visualization for both datasets, and the parameter λ used is set to the default value 10. We use the same initialization generated from the Gaussian distribution as that in the original EE Matlab code.

**Figure 2:**
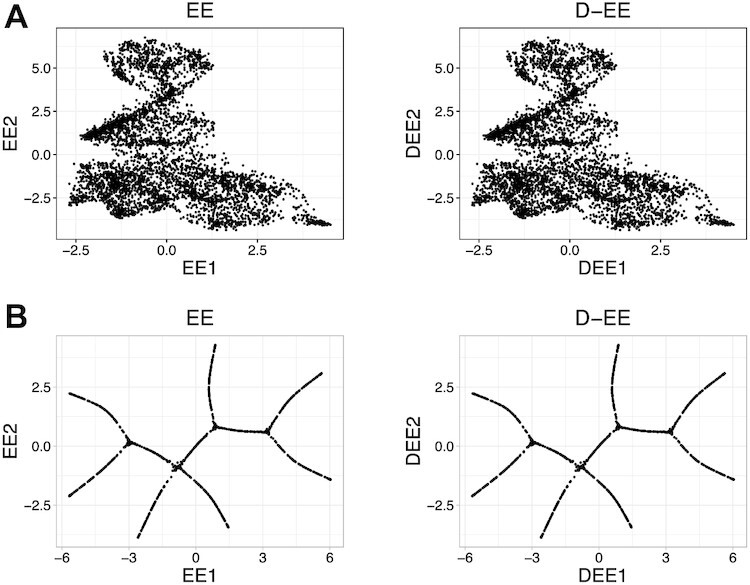
D-EE and EE achieve the same results on 2 datasets when using the same initializations. A: The 2-D mapping of HSPC data obtained by the 2 algorithms. B: The 2-D mapping of PHATE data obtained by the 2 algorithms.

D-EE achieves 2D embedding consistent with that of EE (Fig. 2). To measure the consistency quantitatively, we calculate the relative error, which is defined by

\begin{equation*} \text{relative error}=\frac{\Vert A-B\Vert _{\mathrm{F}}}{\Vert A\Vert _\mathrm{F}}, \end{equation*}

where ‖ · ‖_F_ is the Frobenius norm of the matrix and *A* and *B* represent the output of EE and D-EE, respectively. The Frobenius norm of a matrix $A \in \mathbb {R}^{m\times n}$ is defined as

\begin{equation*} \Vert A\Vert _\mathrm{F}=\sqrt{\sum _{i=1}^m\sum _{j=1}^n |a_{ij}|^2}. \end{equation*}

Their relative errors of D-EE in the HSPC dataset and PHATE dataset are 2.42 × 10^−6^ and 1.60 × 10^−6^, respectively, thus further validating the consistency of results by D-EE and EE.

To test the performance of parallel efficiency of D-EE on the large-scale dataset, we apply D-EE to the iPSC dataset (∼250,000 cells) using 500, 1,000, 2,000, and 4,000 processes, respectively, and for each setting of number of processes we run it at least twice. The averaged computation times of D-EE for the iPSC dataset are 5.83, 3.19, 2.36, and 2.02 hours, when the numbers of processes are 500, 1,000, 2,000, and 4,000, respectively. Next, we evaluate the parallel performance of D-EE based on 2 widely used indexes, the strong scaling speedup ratio and the parallel efficiency. The strong speedup ratio is defined as

\begin{equation*} S=\frac{T_s}{T_p}, \end{equation*}

where *T_s_* and *T_p_* are the time of computation by using a single process and *p* processes, respectively. The parallel efficiency is defined as

\begin{equation*} E=\frac{S}{p}=\frac{T_s}{pT_p}. \end{equation*}

The ideal strong speedup should be *p*, and the corresponding parallel efficiency should be 1 when *p* processes are used. However, it is impossible to run the data with 250,000 samples on a single process owing to limited memory and the low efficiency of the EE algorithm. Thus, we take the time of computation of 500 processes as *T_s_* with *s* = 500, and the parallel efficiency is then reformulated as

\begin{equation*} E=\frac{sT_s}{pT_p}. \end{equation*}

**Figure 3:**
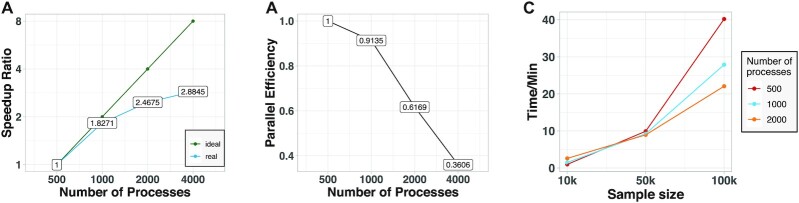
Strong scaling results and parallel efficiency of the D-EE algorithm on an LSSC-IV supercomputer. We apply D-EE on the iPSC dataset by using 500, 1,000, 2,000, and 4,000 processes, respectively. A: The strong speedup ratio increases with increase in the number of processes.The green line represents the ideal speedup ratio and the blue line represents the speedup ratio obtained by D-EE. B: The parallel efficiency decreases at an acceptable rate with the increase in number of processes. C: Computational times consumed for 10,000, 50,000, 100,000 samples under 500, 1,000 and 2,000 processes, respectively.

When adopting the speedup ratio and parallel efficiency as the indexes for scaling, a strong scaling performance at remarkable speedup is observed when increasing CPUs from 500 to 4,000 processes (Fig. 3A and B).

We further evaluate the performance of D-EE on computational times in our supercomputer. Three test cases with sample sizes of 10,000, 50,000, and 100,000 are run on 500, 1,000, and 2,000 processors, respectively. It is shown that when the number of processes is held constant, it is natural that the computational time increases as total sample size increases (as shown in Fig.   3C).

In practice, for analysis of a large-scale single-cell dataset, a workstation with multiple CPUs and large memory is recommended. Meanwhile, we also test D-EE on datasets at different sample sizes and find that D-EE can be efficiently implemented on a typical personal computer (e.g., 8 CPU threads, 16 GB RAM) with a sample size of ≤12,000, while on a conventional workstation (e.g., 40 CPU threads, 256 GB RAM) with a sample size of ≤48,000.

### D-EE and D-TSEE recover intrinsic low-dimensional structures of large-scale scRNA-seq data

**Figure 4:**
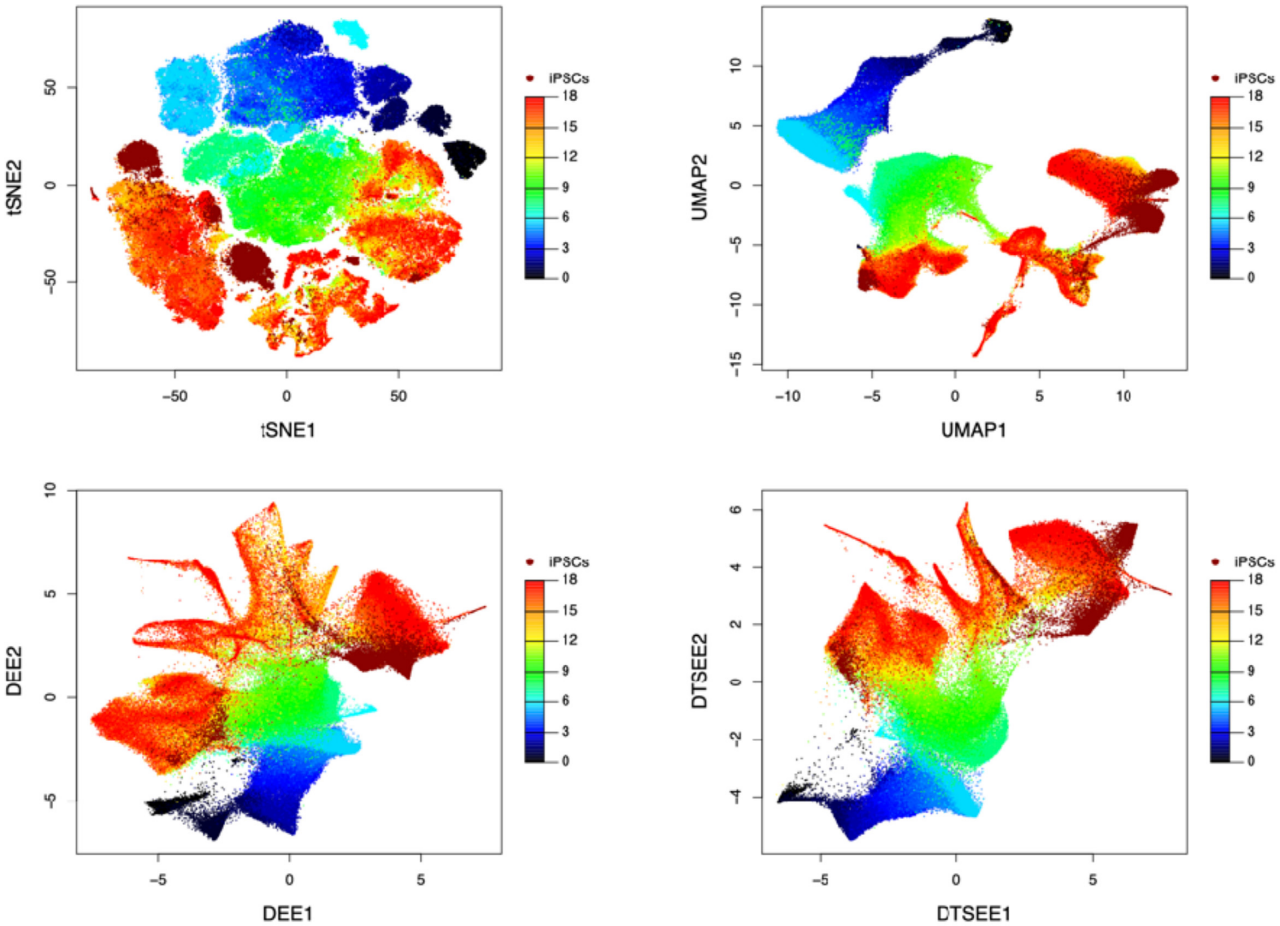
Cells are colored by time stages in the iPSC dataset on the 2D space obtained by 4 dimensionality reduction methods, i.e., t-SNE, UMAP, D-EE, and D-TSEE.

We illustrate the application of D-EE and D-TSEE on the large-scale iPSC dataset. We visualize the iPSC data on the 2D space using t-SNE, UMAP, D-EE, and D-TSEE, respectively (Fig. 4). The same pre-processed single-cell data are used for all 4 methods. The t-SNE is conducted by the FIt-SNE method [23] with a PCA initialization, and we choose the learning rate as 1/12 of the sample size (according to [3]) for better preservation of the global structures. UMAP is conducted by adjusting the number of nearest neighbors (NNs) to balance the preservation of local and global structures. We find that UMAP is not sensitive when choosing the number of NNs from 30 (defaults) to 500 (square root of the number of samples), and we thus set the number of NNs to be 100 in our study. Both t-SNE and UMAP are implemented by the Seurat software (version 3.2.1). In our implementations, both D-EE and D-TSEE are used with their default parameters.

We color the cells on the basis of time stages on their low-dimensional embeddings obtained by the 4 dimensionality reduction results (Fig. 4). We find that t-SNE preserves the time lineage structure of data in an “S” shape with small gaps (Fig. 4, upper left panel); UMAP also shows a time lineage structure but with a large gap occurring between time stages 5.5 and 6 (Fig. 4, upper right panel). In contrast, both D-EE and D-TSEE preserve continuous time lineage structures in low-dimensional space (Fig. 4, lower panels).

**Figure 5:**
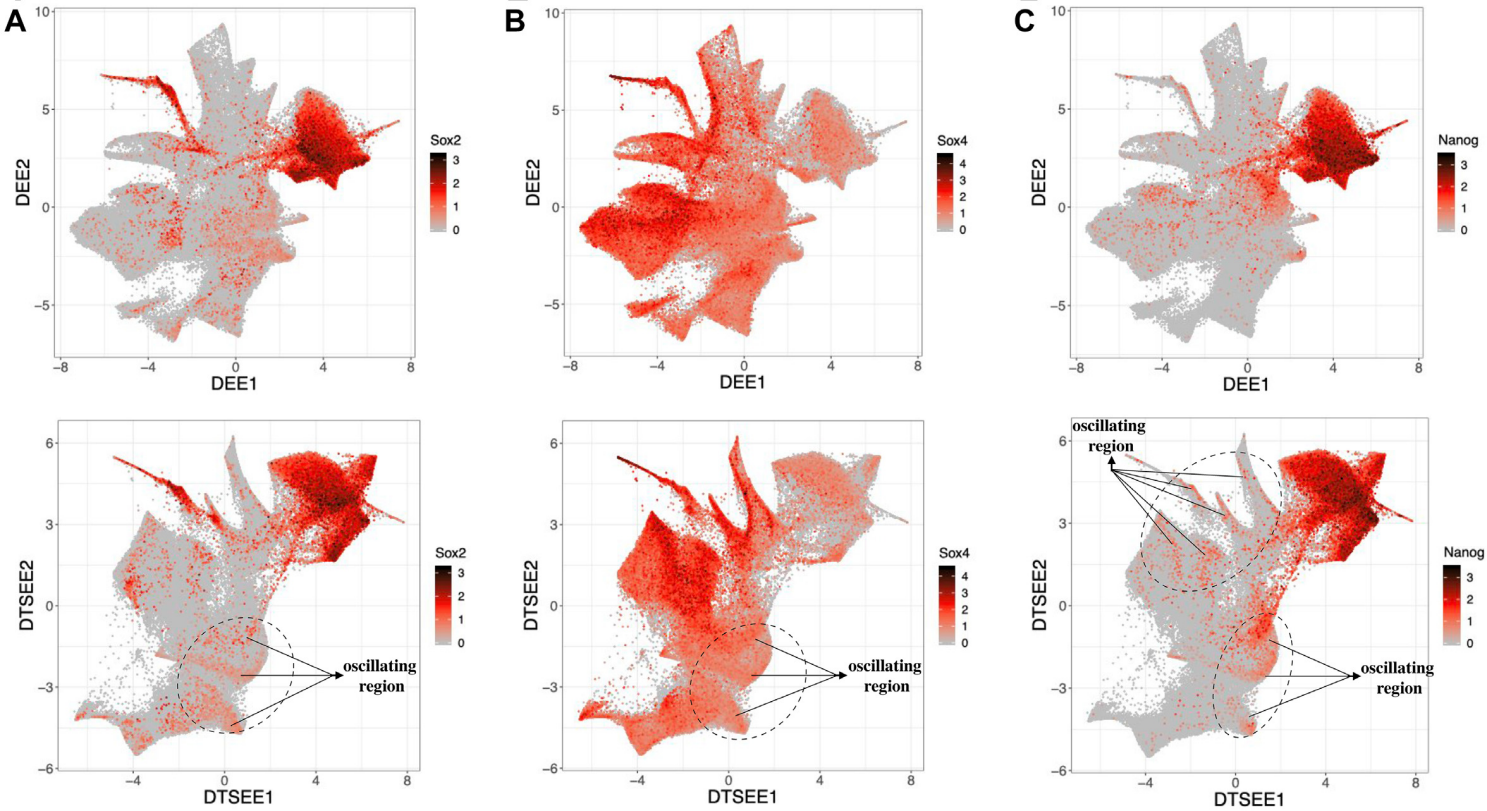
Cells are colored by gene expression of Sox2, Sox4, and Nanog in the iPSC dataset on the 2D embeddings obtained by D-EE and D-TSEE, respectively.

We further explore the gene expression patterns of Sox2, Sox4, and Nanog on both D-EE and D-TSEE embeddings (Fig. 5). These genes are key regulators during stem cell differentiation and reprogramming process [24–26]. Previous study has shown evidence that these genes may oscillate during cell development progression [11,26]. These genes display oscillatory gene expression patterns in the early stage of iPSC on the D-TSEE view (Fig. 5), providing useful information and clues for downstream analysis.

## Conclusion

In this work, we develop a novel tool, D-EE, for visualizing large-scale scRNA-seq data. D-EE implements the distributed storage and distributed computing techniques to a powerful nonlinear dimensionality reduction method, EE. The optimal distributed computational strategies implemented by D-EE allow it to achieve not only strong scalability on large-scale datasets but also the exact optimization solution as original EE by fully utilizing the whole data. Numerical experiments validate the correctness and parallel efficiency of D-EE. Considering the emergence of time-series scRNA-seq data, our D-TSEE tool allows us efficiently to perform dimensionality reduction on large-scale single-cell data by using experimentally temporal information. Besides, when incorporating temporal information if it is available, D-TSEE can reveal dynamic gene expression patterns, providing insights for subsequent analysis of molecular mechanisms and dynamic transition progression.

We demonstrate that D-EE and D-TSEE work efficiently on large-scale datasets at a supercomputer. However, the proposed distributed algorithm D-EE still has disadvantages due to the huge computational cost and storage with a relatively large number of cells. Therefore, D-EE is limited to handling and analyzing huge-scale datasets with the number of cells up to the order of millions [27]. In comparison, the state-of-the-art accelerated implementations of t-SNE (e.g., FIt-SNE) and UMAP are of close-to-linear computational complexities, showing great efficiency in huge data analysis.

In a future study, to resolve the limitation of D-EE on huge-scale data computation, we can accelerate D-EE by adopting either the fast Fourier transform as used in FIt-SNE, or adopting the state-of-the-art neural network framework used by net-SNE [28]. On the other hand, because a huge-scale single-cell dataset can be highly redundant, we can also select a subset of informative samples using an advanced geometric sketching tool [29] prior to application of D-EE.

## Availability of Source Code and Requirements

• Project name: D-EE

• Project home page: https://github.com/ShaokunAn/D-EE

• Operating systems: Linux

• Programming language: C, R

• Other requirements: Multi-core processor, implementation of MPI library (i.e., OpenMPI or IntelMPI) installed on each node of the cluster, a reasonably fast interconnecting infrastructure, PETSc 3.11.4 or higher

• License: GNU General Public License

• biotools: d-ee

• RRID:SCR_019058

## Availability of Supporting Data and Materials

The PHATE data supporting the results of this article are available in the GitHub repository [17]. The iPSC data are available in the NCBI repository with No. GSE122662 [22]. The HSPC data are available in the NCBI repository with accession No. GSE72857 and the dataset used in our study is downloaded from their GitHub repository [30]. An archival copy of the code is available via the*GigaScience* database, GigaDB [31].

## Abbreviations

CAS: Chinese Academy of Sciences; CPU: central processing unit; D-EE: distributed optimization implementation of elastic embedding; D-TSEE: distributed optimization implementation of time-series elastic embedding; EE: elastic embedding; HSPC: hematopoietic stem and progenitor cells; LU: lower–upper; NCBI: National Center for Biotechnology Information; NNs: nearest neighbors; scRNA-seq: single-cell RNA sequencing; PCA: principal component analysis; PETSc: Portable, Extensible Toolkit for Scientific Computation; PHATE: Potential of Heat-diffusion for Affinity-based Transition Embedding; RAM: random access memory; TSEE: time-series elastic embedding; t-SNE: *t*-distributed stochastic neighbor embedding; UMAP: Uniform Manifold Approximation and Projection.

## Competing Interests

The authors declare that they have no competing interests.

## Funding

This work is supported by the National Key R&D Program of China under Grant 2018YFB0704304, NSFC grants (Nos. 11871069, 12071466), NCMIS of Chinese Academy of Sciences (CAS), LSEC of CAS, LSC of CAS, and the Youth Innovation Promotion Association of CAS.

## Authors' Contributions

Conceptualization and Methodology: S.A., L.W. Software: S.A., J.H. Supervision: S.A., L.W., J.H. Funding Acquisition: L.W., J.H. Writing—Original Draft Preparation: S.A. Writing—Review & Editing: all authors.

## Supplementary Material

giaa126_GIGA-D-20-00236_Original_Submission

giaa126_GIGA-D-20-00236_Revision_1

giaa126_GIGA-D-20-00236_Revision_2

giaa126_Response_to_Reviewer_Comments_Original_Submission

giaa126_Response_to_Reviewer_Comments_Revision_1

giaa126_Reviewer_1_Report_Original_Submission Brian Hie -- 8/18/2020 Reviewed

giaa126_Reviewer_1_Report_Revision_1 Brian Hie -- 10/1/2020 Reviewed

giaa126_Reviewer_2_Report_Original_Submission Dmitry Kobak -- 9/15/2020 Reviewed

giaa126_Reviewer_2_Report_Revision_1 Dmitry Kobak -- 10/7/2020 Reviewed
