# Supplementary material for: D-EE: Distributed software for visualizing intrinsic structure of large-scale single-cell data
Source: Gigascience. 2020 Nov 11;9(11):giaa126. doi: 10.1093/gigascience/giaa126 (PMC7657844; doi:10.1093/gigascience/giaa126)
Supplement: giaa126_GIGA-D-20-00236_Original_Submission [file giaa126_giga-d-20-00236_original_submission.pdf]

GigaScience

D-EE: a distributed software for visualizing intrinsic structure of large-scale single-cell data

--Manuscript Draft--

**Manuscript Number:**	GIGA-D-20-00236
**Full Title:**	D-EE: a distributed software for visualizing intrinsic structure of large-scale single-cell data
**Article Type:**	Technical Note
**Funding Information:**	National Key R&D Program of China (2018YFB0704304) — Dr. Lin Wan
**Abstract:**	Background: Dimensionality reduction and visualization play vital roles in single-cell RNA sequencing (scRNA-seq) data analysis. While they have been extensively studied, state-of-the-art dimensionality reduction algorithms are often unable to preserve the global structures underlying data. Elastic Embedding (EE), a nonlinear dimensionality reduction method, has shown promise in revealing low-dimensional intrinsic local and global data structure. However, the current implementation of the EE algorithm lacks scalability to large-scale scRNA-seq data.<br><br>Results: We present a distributed optimization implementation of the EE algorithm, termed distributed Elastic Embedding (D-EE). D-EE reveals the low-dimensional intrinsic structures of data with accuracy equal to that of Elastic Embedding, and it is scalable to large-scale scRNA-seq data. It leverages distributed storage and distributed computation, achieving memory efficiency and high-performance computing simultaneously. In addition, an extended version of D-EE, termed distributed optimization implementation of time series Elastic Embedding (D-TSEE), enables the user to visualize large-scale time series scRNA-seq data by incorporating experimental temporal information. Results with a large-scale scRNA-seq data indicate D-TSEE can uncover oscillatory gene expression patterns by employing experimentally temporal information.<br><br>Conclusions: D-EE is a distributed dimensionality reduction and visualization tool. Its distributed storage and distributed computation technique allow us to efficiently analyze large-scale single-cell data. The source code for D-EE algorithm based on C and MPI tailored to a High Performance Computing cluster is available at https://github.com/ShaokunAn/D-EE.
**Corresponding Author:**	Lin Wan<br>Academy of Mathematics and Systems Science, Chinese Academy of Sciences<br>Beijing, CHINA
**Corresponding Author Secondary Information:**	
**Corresponding Author's Institution:**	Academy of Mathematics and Systems Science, Chinese Academy of Sciences
**Corresponding Author's Secondary Institution:**	
**First Author:**	Shaokun An
**First Author Secondary Information:**	
**Order of Authors:**	Shaokun An
	Jizu Huang
	Lin Wan
**Order of Authors Secondary Information:**	
**Additional Information:**	
**Question**	**Response**

Powered by Editorial Manager® and ProduXion Manager® from Aries Systems Corporation

Are you submitting this manuscript to a special series or article collection?	No
**Experimental design and statistics**<br><br>Full details of the experimental design and statistical methods used should be given in the Methods section, as detailed in our Minimum Standards Reporting Checklist. Information essential to interpreting the data presented should be made available in the figure legends.<br><br>Have you included all the information requested in your manuscript?	Yes
**Resources**<br><br>A description of all resources used, including antibodies, cell lines, animals and software tools, with enough information to allow them to be uniquely identified, should be included in the Methods section. Authors are strongly encouraged to cite Research Resource Identifiers (RRIDs) for antibodies, model organisms and tools, where possible.<br><br>Have you included the information requested as detailed in our Minimum Standards Reporting Checklist?	Yes
**Availability of data and materials**<br><br>All datasets and code on which the conclusions of the paper rely must be either included in your submission or deposited in publicly available repositories (where available and ethically appropriate), referencing such data using a unique identifier in the references and in the "Availability of Data and Materials" section of your manuscript.<br><br>Have you have met the above requirement as detailed in our Minimum	Yes

Standards Reporting Checklist?	

Manuscript PDF

Click here to access/download;Manuscript;1 Reference PDF.pdf

GigaScience, 2020, 1–8

doi: xx.xxxx/xxxx
Manuscript in Preparation
Technical Note

TECHNICAL NOTE

D-EE: a distributed software for visualizing intrinsic structure of large-scale single-cell data

Shaokun An[1,2], Jizu Huang[1,2,*] and Lin Wan[1,2,*]

[1]NCMIS, LSEC, LSC, Academy of Mathematics and Systems Science, Chinese Academy of Sciences, Beijing, 100190, China and [2]School of Mathematical Sciences, University of Chinese Academy of Sciences, Beijing, 100049, China

[*]huangjz@lsec.cc.ac.cn; lwan@amss.ac.cn

Abstract

Background: Dimensionality reduction and visualization play vital roles in single-cell RNA sequencing (scRNA-seq) data analysis. While they have been extensively studied, state-of-the-art dimensionality reduction algorithms are often unable to preserve the global structures underlying data. Elastic Embedding (EE), a nonlinear dimensionality reduction method, has shown promise in revealing low-dimensional intrinsic local and global data structure. However, the current implementation of the EE algorithm lacks scalability to large-scale scRNA-seq data. **Results**: We present a distributed optimization implementation of the EE algorithm, termed distributed Elastic Embedding (D-EE). D-EE reveals the low-dimensional intrinsic structures of data with accuracy equal to that of Elastic Embedding, and it is scalable to large-scale scRNA-seq data. It leverages distributed storage and distributed computation, achieving memory efficiency and high-performance computing simultaneously. In addition, an extended version of D-EE, termed distributed optimization implementation of time series Elastic Embedding (D-TSEE), enables the user to visualize large-scale time series scRNA-seq data by incorporating experimental temporal information. Results with a large-scale scRNA-seq data indicate D-TSEE can uncover oscillatory gene expression patterns by employing experimentally temporal information. **Conclusions**: D-EE is a distributed dimensionality reduction and visualization tool. Its distributed storage and distributed computation technique allow us to efficiently analyze large-scale single-cell data. The source code for D-EE algorithm based on C and MPI tailored to a High Performance Computing cluster is available at https://github.com/ShaokunAn/D-EE.

Key words: dimensionality reduction; distributed storage; distributed computation; large-scale data; single-cell sequencing.

Background

The advent of single-cell sequencing provides high-dimensional profiles of cellular states at single-cell resolutions (e.g., single-cell RNA sequencing (scRNA-seq) of transcriptomes), offering the opportunity to unveil intrinsic biological processes and mechanisms. Dimensionality reduction and visualization methods have been extensively studied, as they play vital roles in revealing the intrinsic structures underlying scRNA-seq high-dimensional data [1]. Nonetheless, it is still challenging for these state-of-the-art methods of dimensionality reduction and visualization to preserve both local and global structures of data in low-dimensional space. For example, the celebrated t-distributed Stochastic Neighbor Embedding (t-SNE) algorithm [2] is widely used in the single-cell community. It emphasizes the preservation of local structures, but it often distorts global structures [3, 4, 5]. As a solution, the Uniform Manifold Approximation and Projection (UMAP) algorithm [6] was developed, showing better performances than that of t-SNE on preserving the global structures of data [7]. However, a recent study showed that UMAP does not improve upon t-SNE in this regard when using the same initialization [8], making the validity of UMAP debatable.

In contrast, Elastic Embedding (EE), a nonlinear dimensionality reduction method, attempts to preserve both local and

Compiled on: August 9, 2020.
Draft manuscript prepared by the author.

global structures underlying the data [3]. To achieve this goal, EE extends t-SNE by penalizing placement of latent points in close proximity away from dissimilar data points in high-dimensional space, thus resolving the difficulty of global structure preservation (see [3], or Methods for details). EE has attracted increasing interest among statistical researchers [9]. It has also shown remarkable performance on visualizing the intrinsic structures of scRNA-seq data [1, 4, 10]. However, the current implementations of the EE algorithm is not scalable to sample size N (e.g., number of cells). Thus, it cannot be used for large-scale scRNA-seq datasets. For example, the storage of the attractive and the repulsive weight matrixes of the EE algorithm is $\mathcal{O}(N^2)$.

Therefore, we present a distributed optimization implementation of EE, termed D-EE. D-EE not only reveals the low-dimensional intrinsic structures of data with the same accuracy as EE, but also is scalable to large-scale scRNA-seq data. It leverages distributed storage and distributed computation, achieving memory efficiency and high-performance computing simultaneously (Figure 1). In addition, a distributed optimization implementation of the time series EE (TSEE) algorithm [10], termed D-TSEE, is also provided for visualizing large-scale time series scRNA-seq data. In this study, we demonstrate the power of D-EE and D-TSEE on both simulated and real data. Both D-EE and D-TSEE (1) achieve the same accuracy as EE and TSEE, respectively, outperforming t-SNE and UMAP in preserving the global structures; (2) gain high strong scaling performance on large-scale dataset.

Methods

Elastic Embedding algorithm

EE was proposed by [3]. It optimizes an energy function containing the attractive and repulsive terms.

Given N samples $\mathbf{Y} = \{y_1, y_2, \ldots, y_N\}$, where $y_i \in \mathbb{R}^D$ represents its high-dimensional coordinates, the goal of EE is to map the data from high-dimensional space onto a low-dimensional representation $\mathbf{X} = \{x_1, x_2, \ldots, x_N\}$ with $x_i \in \mathbb{R}^d$ and $d \ll D$ by minimizing an energy function

$$E(\mathbf{X}, \lambda) = \sum_{m,n=1}^{N} w^{+}_{nm} \|x_n - x_m\|^2 + \lambda \sum_{m,n=1}^{N} w^{-}_{nm} \exp(-\|x_n - x_m\|^2),$$

where $w^{+}_{nm} = \exp(-\frac{1}{2}\|y_n - y_m\|^2/\sigma_n^2)$ and $w^{-}_{nm} = \|y_n - y_m\|^2$. The first term acts as an *attractive* force to preserve local distances, while the second term acts as a *repulsive* force to preserve global structures or to separate latent points. The parameter $\lambda \in \mathbb{R}^+$ trades off the two terms, and a larger value implies preservation of global structures is more important. The parameters $\{\sigma_m\}_{m=1}^N$ in w^{+}_{mn} are obtained based on the distance matrix $\mathbf{D} = \{d_{mn}\}$ by solving a series of root-finding problems [11].

An extension of EE, TSEE [10], was recently proposed to handle the dimensionality reduction problems of time series scRNA-seq data. It works by minimizing

$$E(\mathbf{X}, \lambda) = \sum_{m,n=1}^{N} w^{+}_{nm} \|x_n - x_m\|^2 + \lambda \sum_{m,n=1}^{N} (w^{-}_{nm} + \beta t_{nm}) \exp(-\|x_n - x_m\|^2),$$

where t_{nm} represents the dissimilarity of time of pairwise points, and β trades off the weights between dissimilarities of time stages and expression space.

Numerical optimization of EE

Since the optimization solution of TSEE is basically the same as that of EE, we only give the numerical solution of EE. First, we denote $\mathbf{W_P} = \{w^{+}_{nm}\}$ and $\mathbf{W_N} = \{w^{-}_{nm}\}$. Owing to the existence of parameters $\{\sigma_n\}$, $\mathbf{W_P}$ is not a symmetrical matrix but we make it to be symmetric by taking $\mathbf{W_P} := \mathbf{W_P} + \mathbf{W_P}^T$. Next, the diagonal elements of $\mathbf{W_P}$ and $\mathbf{W_N}$ are set to zero. Finally, each element is normalized by dividing the sum of all elements in the matrix.

To solve the optimization problem, the classic Quasi-Newton methods update $\mathbf{X_{k+1}}$ according to $\mathbf{X_{k+1}} = \mathbf{X_k} + \alpha_\mathbf{k} \mathbf{P_k}$ in the k-th iteration, where $\alpha_\mathbf{k}$ is the step length determined by a line search procedure, and $\mathbf{P_k}$ is the search direction obtained by solving a Jacobian system $\mathbf{B_k P_k} = -\mathbf{G_k}$. In this equation, $\mathbf{B_k}$ is positive-definite to guarantee the decrease of objective function. $\mathbf{G_k} = \mathbf{L_k X_k}$ is the gradient of the objective function in the k-th iteration, where $\mathbf{L_k}$ is the Laplacian of $\mathbf{W_k} = \{w^{(k)}_{mn}\}$ with $w^{(k)}_{mn} = w^{+}_{mn} - \lambda w^{-}_{mn} \exp(-\|x^{(k)}_n - x^{(k)}_m\|^2)$. These procedures are repeated until a certain termination criterion is satisfied. During the iteration, $\mathbf{B_k}$ generally needs to be updated in each iteration as well.

When optimizing the EE-like optimization problems, a technique termed Partial-Hessian optimization strategies has been proposed to employ partial information of Hessian $\mathbf{L_P}$ [12], which is the Laplacian of $\mathbf{W_P}$ and is invariant in each iteration. This invariance makes it possible to utilize some precondition approaches, e.g., LU decomposition, to improve calculation efficiency. The effectiveness of the determined direction, called Spectral Direction, has been validated experimentally in previous work [12].

D-EE algorithm

We provide a distributed optimization implementation of EE, termed D-EE. The overview of the newly proposed D-EE algorithm is given in Figure 1. During whole optimization implementation, multiple processes are employed for computation and storage of data. In Figure 1, two processes, $\mathcal{P}_0$ and $\mathcal{P}_1$, are taken as an example. To achieve high performance in computing and memory efficiency simultaneously, our proposed distributed algorithm divides data ($\mathbf{W_P}$, $\mathbf{W_N}$, and $\mathbf{G_k}$) by rows for the multiple processes assigned. To avoid frequent communication, the whole original high-dimensional data $\mathbf{Y}$ is read and stored in each process, and the low-dimensional embedding $\mathbf{X}$ is established in each process as well since the storage consumed by $\mathbf{Y}$ and $\mathbf{X}$ is much less when compared to other $N \times N$ matrixes used during computation. It is worth to note that, since most of the computation of each row in one matrix generally merely depends on the same row of other matrixes (see the approximated computational complexity of D-EE in the following section for details), the partition procedure we design for in the D-EE algorithm is an almost optimal partition in parallel computing as a result of the optimal leverage of computation and communication. On the one hand, the total computational cost of the D-EE algorithm is almost the same as that of the centralized algorithm of EE. On the other hand, most procedures in the D-EE algorithm are communication-free, as shown in Figure 1 by black arrow. Even though some procedures still exist with communication, as shown in Figure 1 by blue arrow, the communication volume is in a much lower order than the cost of computation.

Computation of matrixes $\mathbf{D}, \mathbf{W_P}, \mathbf{W_N}$

As mentioned before, the matrixes $\mathbf{W_P}, \mathbf{W_N}$ depend on the high-dimensional data $\mathbf{Y}$ and $\{\sigma_n\}_{n=1}^N$. Since each σ_n is obtained by solving a root-finding problem from the n-th row of the distance matrix $\mathbf{D}$, each matrix is equally, or almost equally, partitioned into multiple nonoverlapping parts by row and stored in multiple processes, as shown in Figure 1A. Let us denote $\mathbf{D} = [\mathbf{D}^1, \cdots, \mathbf{D}^P]$, where sub-matrix $\mathbf{D}^i$ with size of $M_i \times N$ is stored in the i-th process, and P is the number of processes we used. The $[\cdots]$ represents a column vector. Similar notations are used for the other $N \times N$ matrixes. It is clear that each row of matrixes $\mathbf{D}, \mathbf{W_P}, \mathbf{W_N}$ depends on all original high-dimensional data $\mathbf{Y}$. Therefore, we load a copy of $\mathbf{Y}$ into each process to avoid communication.

In the centralized implementation of EE, the parameters $\sigma_n, n = 1, \ldots, N$, are calculated by iteratively solving a sequence of root-finding problems. The iteration method for the root-finding problems is improved by reordering the computation of $\{\sigma_n\}_{n=1}^N$ according to the distances of all samples ($\mathbf{Y}$), which is also the complete distance matrix $\mathbf{D}$ [11]. Then the reordered root-finding problems are sequentially solved by taking the solution of the previous one as the initial value of the next. Since the parameters are distributed in different processes, it is clear that the sequential root-finding approach cannot be parallelized without modifications. In the D-EE algorithm, we calculate $\{\sigma_n\}_{n=1}^N$ in the following parallel way. First, we decompose $\{\sigma_n\}_{n=1}^N$ into P subsets as $\Sigma_i = \{\sigma_n\}_{n=\mathcal{M}_i+1}^{\mathcal{M}_{i+1}}$ with $i = 0, \cdots, P-1$. The elements in the i-th subset Σ_i are computed and stored in the i-th process. Similar to the centralized algorithm of EE, we then reorder Σ_i according to the distance matrix $\mathbf{D}^i$ and iteratively solve the corresponding root-finding problems within the i-th process. According to the distributions of the initial data $\mathbf{Y}$ and the matrixes established before, we conclude that the D-EE algorithm calculates $\{\sigma_n\}_{n=1}^N$ in parallel, which is communication-free. The efficiency of the root-finding approach is also guaranteed by the local order. Since we only change the order and initial guesses of the root-finding problems, the solutions of the root-finding problems, as obtained from D-EE, are almost the same as those from EE. With the whole original high-dimensional data $\mathbf{Y}$ and the subset Σ_i, we can compute the following submatrixes $\mathbf{W_P}^i$, $\mathbf{W_N}^i$ in the i-th process. Thus, we give a parallel and communication-free approach to compute matrixes $\mathbf{D}$, $\mathbf{W_P}$, $\mathbf{W_N}$.

Normalization of $\mathbf{W_P}$ and $\mathbf{W_N}$

After computing matrixes $\mathbf{W_P}$, $\mathbf{W_N}$, each process sets the diagonal elements belonging to it as 0 in parallel. Then, we set $\mathbf{W_P} := \mathbf{W_P} + \mathbf{W_P}^T$ such that $\mathbf{W_P}$ becomes a symmetric matrix. Let us denote $\mathbf{W_P}^T := \hat{\mathbf{W}}_\mathbf{P} = [\hat{\mathbf{W}}_\mathbf{P}^1, \cdots, \hat{\mathbf{W}}_\mathbf{P}^P]$, where submatrix $\hat{\mathbf{W}}_\mathbf{P}^i$ has the size of $M_i \times N$. In the i-th process, we first obtain the elements of the submatrix $\hat{\mathbf{W}}_\mathbf{P}^i$ from the other $P-1$ processes by communication and then compute $\mathbf{W_P}^i := \mathbf{W_P}^i + \hat{\mathbf{W}}_\mathbf{P}^i$. Here point-to-point communication happens, and the communication volume for each process is $\mathcal{O}(N^2/P)$.

To normalize the matrixes $\mathbf{W_P}$, $\mathbf{W_N}$, each element should be divided by the sum of all elements in the matrix. The sum of all elements in matrix $\mathbf{W_P}$ is parallel computed as follows. First, each process calculates the sum of all elements in the submatrix $\mathbf{W_P}^i$ independently. We denote the sum of all elements in the submatrix $\mathbf{W_P}$ and $\mathbf{W_P}^i$ as $\mathcal{S}$ and $\mathcal{S}^i$, respectively. Then we compute the sum of all elements in matrix $\mathbf{W_P}$ by $\mathcal{S} = \sum_{i=1}^P \mathcal{S}_i$ through an MPI_Allgather action. Here all-to-all communication happens, and the communication volume for each process is $\mathcal{O}(P)$. Then, we normalize matrix $\mathbf{W_P}$ in each process by taking $\mathbf{W_P}^i = \mathbf{W_P}^i/\mathcal{S}$ in parallel without communication. The normalization of matrix $\mathbf{W_N}$ is done in a similar way.

Computation of low-dimensional embedding $\mathbf{X}$

After normalizing $\mathbf{W_P}$, its Laplacian $\mathbf{L_P}$, which is needed for the subsequent determination of descent direction, is computed in parallel as follows. In the i-th process, we calculate the elements of submatrix $\mathbf{L_P}^i$ by using $l_{mn}^+ = \sum_{k=1}^N w_{mk}^+ - w_{mn}^+$, where l_{mn}^+ and w_{mn}^+ are the elements of matrixes $\mathbf{L_P}$ and $\mathbf{W_P}$, respectively. Since the two matrixes are partitioned by row in the same way, the computation of $\mathbf{L_P}$ is also communication-free.

The low-dimensional embedding $\mathbf{X}$ is obtained by solving the optimization problem with the Partial-Hessian optimization strategy. During the Quasi-Newton procedures, a dense linear system $\mathbf{L_P}\mathbf{P_k} = -\mathbf{G_k}$ must be solved in parallel. In the D-EE algorithm, we perform LU decomposition on $\mathbf{L_P}$. Considering that $\mathbf{L_P}$ is positive semi-definite, but not positive definite, a small value μ is added to the diagonal of $\mathbf{L_P}$ in practice. During the following sections, we still use $\mathbf{L_P}$ to denote the adjusted matrix. LU decomposition on $\mathbf{L_P} = \mathcal{L}\mathcal{U}$ is done with PETSc, which provides uniform and efficient access to all linear system solvers in the package, including parallel and sequential, direct and iterative [13, 14, 15]. Here, $\mathcal{L}$ and $\mathcal{U}$ are the corresponding lower and upper triangle matrixes, respectively. With the decomposition of LU, the dense linear system $\mathbf{L_P}\mathbf{P_k} = -\mathbf{G_k}$ is replaced by two sublinear systems $\mathcal{L}\hat{\mathbf{P}}_\mathbf{k} = -\mathbf{G_k}$ and $\mathcal{U}\mathbf{P_k} = \hat{\mathbf{P}}_\mathbf{k}$, which can be solved by the backward substitution method.

As shown in Figure 1D, the partitions of $\mathbf{L_P}$, $\mathcal{L}$, and $\mathcal{U}$ are the same as $\mathbf{W_P}$. Let us denote $\mathbf{P_k} = [\mathbf{P_k}^1, \cdots, \mathbf{P_k}^P]$, where submatrix $\mathbf{P_k}^i$ with size of $M_i \times d$ is stored in the i-th process, and a similar partition is performed on $\mathbf{G_k}$. Based on the partitions of $\mathbf{L_P}$, $\mathcal{L}$, $\mathcal{U}$, $\mathbf{P_k}$, and $\mathbf{G_k}$, the computational complexities per process of LU decomposition and backward substitution are $\mathcal{O}(N^3/P)$ and $\mathcal{O}(N^2/P)$, with corresponding communication volumes of $\mathcal{O}(N^2/P)$ and $\mathcal{O}(N/P)$, respectively. According to the analysis, LU decomposition is only done in the first iteration of the Quasi-Newton method, and matrixes $\mathcal{L}$ and $\mathcal{U}$ are stored and reused during the whole Quasi-Newton procedure.

The gradient $\mathbf{G_k}$ in the right-hand side of the linear system $\mathbf{L_P}\mathbf{P_k} = -\mathbf{G_k}$ is calculated by $\mathbf{G_k} = \mathbf{L_k}\mathbf{X_k}$, where the $N \times N$ matrix $\mathbf{L_k}$ depends on matrixes $\mathbf{W_P}$, $\mathbf{W_N}$, and $\mathbf{Ker}$. Here the elements of matrix $\mathbf{Ker}$ are defined as $\mathrm{ker}_{mn} = \exp(-\|x_m - x_n\|^2)$, and the elements of matrix $\mathbf{L_k}$ are defined as $l_{mn}^{(k)} = w_{mn}^+ - \lambda w_{mn}^- \mathrm{ker}_{mn}^{(k)}$. As shown in Figure 1E, the partitions of matrixes $\mathbf{L_k}$ and $\mathbf{Ker}$ are the same as those of $\mathbf{W_P}$. In D-EE, we store all elements of $\mathbf{X_k}$ in each process, which is the same as the original high-dimensional data $\mathbf{Y}$. Thus, we can parallel compute matrixes $\mathbf{L_k}$ and $\mathbf{Ker}$ in the same way with the matrix $\mathbf{W_N}$, which means the procedure is also communication-free.

After solving the linear system, we obtain the search direction $\mathbf{P_k}$. Then, we update $\mathbf{X_{k+1}}$ according to $\mathbf{X_{k+1}} = \mathbf{X_k} + \alpha_k \mathbf{P_k}$, where α_k is determined by a line search approach. As mentioned before, the low-dimensional embedding $\mathbf{X_k}$ is stored sequentially, but $\mathbf{P_k}$ is distributed stored. Thus, we first compute the elements of submatrix $\mathbf{P_{k+1}}^i$ in the i-th process and then gather all elements of $\mathbf{P_k}$ in each process by the all-gather function in MPI. Here all-to-all communication happens, and the order of communication volume for each process is $\mathcal{O}(Nd)$. In line search steps, we need to calculate the energy function $E(\mathbf{X}, \lambda)$ several times, which is computed in parallel according

to the following formula

$$E(\mathbf{X}, \lambda) = \sum_{i=1}^{P} \left(\sum_{m=\mathcal{M}_i+1}^{\mathcal{M}_{i+1}} \sum_{n=1}^{N} \left\{ w_{mn}^{+} \|x_n - x_m\|^2 + \lambda w_{mn}^{-} \exp(-\|x_n - x_m\|^2) \right\} \right).$$

The summation included in the parentheses is calculated in each process simultaneously and then gathered by the MPI all-gather function. Here all-to-all communication happens, and the communication volume for each process is $\mathcal{O}(P)$.

Results

Data Description

We test the accuracy and scalability of D-EE on three datasets. The first simulated dataset [16], named PHATE data for convenience, consists of 1,440 samples and 60 features. It's a complex tree structure which simulates a cellular developmental process, namely, progressions, branch or split in progressions and end state of progression, composed of ten branches in total. We first perform principal component analysis (PCA) on the original data, reserving a 1,440 samples $\times$ 7 features matrix.

The second dataset characterizes process of mouse hematopoietic stem and progenitor cells (HSPCs) bifurcating to myeloid and erythroid precursors [17], named HSPCs data, consisting of 4,423 samples. The obtained raw data is pre-processed by Seurat package [18, 19]. We filter cells and genes with default parameters, perform logarithmic transformation, find variable features and finally perform PCA on it, obtaining a 4,423 samples $\times$ 50 features matrix as input of EE and D-EE.

The third data is a large-scale time series scRNA-seq dataset containing ~250k cells [20]. The data characterizes reprogramming of fibroblasts to induced pluripotent stem cells (iPSCs), which was collected at half-day intervals across 18 days, resulting in 39 time points. Since the final time point of the iPSCs status was not annotated temporally, we therefore set the final point as 20-th day as the input to D-TSEE. We pre-process this data with Seurat package as well. Same as the pre-process of HSPCs data, we first filter cells and genes to include cells where at least 200 features are detected and to include genes detected in at least 50 cells, obtaining 259,081 cells and 19,427 genes. After that, we perform logarithmic transformation, find variable features and perform PCA, obtaining a 259,081 samples $\times$ 50 features matrix as input of dimensionality reduction methods.

D-EE achieves high strong scaling efficiency

We evaluate D-EE using both simulated and real scRNA-seq datasets. First, we employ PHATE data and HSPCs data to test the consistency between D-EE and EE results. We employ 36 processes in both D-EE algorithms. The low dimensions are set to 2 for the convenience of visualization for both datasets, and the parameter λ used is set to the default values. Figure 2 displays the visually consistent dimensionality reduction results. Their numeric relative errors are 2.420804×10^{-6} and 1.596752×10^{-6} (Frobenius norm), respectively, thus validating the consistency of results by D-EE and EE.

To test the scaling of D-EE, we use iPSCs data and apply D-EE using 500, 1,000, 2,000, and 4,000 processes, respectively. We mainly focus on the strong scaling speedup ratio and parallel efficiency of D-EE software, two important indexes by which to characterize the efficiency of a parallel algorithm. The strong speedup ratio is defined as

$$S = \frac{T_s}{T_p},$$

where T_s and T_p are the time of computation by using single process and p processes, respectively. The parallel efficiency is defined as

$$E = \frac{S}{p} = \frac{T_s}{pT_p}.$$

The ideal strong speedup should be p, and the corresponding parallel efficiency should be 1 when p processes are used. However, it is impossible to run the data with 250k samples on a single process owing to limited memory and low efficiency of EE algorithm. Thus, we take the time of computation of 500 processes as T_s with $s = 500$, and the parallel efficiency is then reformulated as

$$E = \frac{sT_s}{pT_p}.$$

We apply D-EE to iPSCs data [20], using 500, 1,000, 2,000 and 4,000 CPU processes, respectively. When adopting the speedup ratio and parallel efficiency as the indexes for scaling, a strong scaling performance at remarkable speedup is observed when increasing CPUs from 500 to 4,000 processes (Figure 3).

D-EE and D-TSEE outperform t-SNE and UMAP in recovering intrinsic low-dimensional structures of large-scale scRNA-seq data

We demonstrate that D-EE and D-TSEE show their superiority over t-SNE and UMAP on preserving the intrinsic low-dimensional structure hidden in the single-cell data. We explore the iPSCs data with t-SNE, UMAP, D-EE and D-TSEE to uncover potential dynamics of reprogramming of fibroblasts to iPSCs. The input of t-SNE, UMAP and D-EE is the pre-processed PCA matrix while the input of D-TSEE needs both gene expression matrix and time label of each sample since D-TSEE is aim to deal with time series high-dimensional data.

We color the cells based on time label on their low-dimensional embeddings obtained by the four dimensionality reduction results (Figure 4). We find that t-SNE breaks the continuous time structures of data, even disturbing time order of day 1 and day 3 (Figure 4, upper-left panel). UMAP also breaks the continuous time structures, with large gap occurring between day 5.5 and day 6 (Figure 4, upper-right panel), and tends to collapse data points into dense clusters (Figure 4, upper-right panel) compared with the other three embeddings, which is unfavorable to explore more subtle and further detailed structures. In contrast, both D-EE and D-TSEE preserve whole time order in low-dimensional space as well as maintain its continuous structures in time. Furthermore, D-TSEE shows gradual change as time propagates (Figure 4, lower-right panel), while D-EE shows a noisier temporal result (Figure 4, lower-left panel). The difference indicates that D-TSEE gains temporal resolution by integrating time point information of cells.

We further explore the gene expression patterns of Sox2,

Sox4 and Nanog on both D-EE and D-TSEE embeddings (Figure 5). These genes are key regulators during stem cell differentiation and reprogramming process [21, 22, 23]. Previous study has shown evidence that these genes may oscillate during cell development progression [10, 23]. We also find that these genes display oscillatory gene expression patterns in the early stage of iPSCs on the D-TSEE embedding. Therefore, D-TSEE provides novel insights and perspectives for subsequent dynamic modeling of the complex processes based on large-scale scRNA-seq data.

Conclusion

In this work, we develop a novel tool, D-EE, for visualizing large-scale scRNA-seq data. D-EE implements the distributed storage and distributed computing techniques to a powerful nonlinear dimensionality reduction method, Elastic Embedding. The optimal distributed computational strategies implemented by D-EE allowing it to achieve not only the strong scalability on large-scale dataset, but also the exact optimization solution as original EE by fully utilizing the whole data. Numerical experiments validate the correctness and parallel efficiency of D-EE. Considering the emergence of time series scRNA-seq data, our D-TSEE tool allow us efficiently to perform dimensionality reduction to large-scale single-cell data by employing experimentally temporal information. The temporal distribution on the results of four dimensionality reduction methods demonstrates that D-EE and D-TSEE outperforms UMAP and t-SNE in uncovering low-dimensional structures when analyzing large-scale single-cell data. Besides, when incorporating temporal information if it's available, D-TSEE can reveal dynamic gene expression patterns, providing insights for subsequent analysis of molecular mechanisms and dynamic transition progression.

Availability of source code and requirements

Lists the following:

- Project name: D-EE
- Project home page: https://github.com/ShaokunAn/D-EE
- Operating system(s): Linux
- Programming language: C, R
- Other requirements: Multi-core processor, implementation of MPI library (i.e., OpenMPI or IntelMPI) installed on each node of the cluster, a reasonably fast interconnecting infrastructure, PETSc 3.11.4 or higher
- License: GNU General Public License

Availability of supporting data and materials

The PHATE data supporting the results of this article is available in the Github repository [16]. The iPSCs data is available in NCBI repository with number GSE 122662 [20]. The HPSCs data is available in the NCBI repository with accession number GSE72857 and the dataset used in our study is downloaded from their Github https://github.com/ManuSetty/wishbone.

Declarations

List of abbreviations

D-EE: distributed optimization implementation of Elastic Embedding; D-TSEE: distributed optimization implementation of time series Elastic Embedding; EE: Elastic Embedding; scRNA-seq: single-cell RNA sequencing; PCA: Principal Component Analysis; TSEE: time series Elastic Embedding; t-SNE: t-distributed Stochastic Neighbor Embedding; UMAP: Uniform Manifold Approximation and Projection.

Consent for publication

Not applicable.

Competing Interests

The authors declare that they have no competing interests.

Funding

This work is supported by the National Key R&D Program of China under Grant 2018YFB0704304, NSFC grants (Nos.11571349, Nos.11871069), NCMIS of CAS, LSEC of CAS, LSC of CAS, and the Youth Innovation Promotion Association of CAS. S.A. thanks the above fundings for offering computing equipment.

Author's Contributions

Conceptualization and Methodology: S.A., L.W. Software: S.A., J.H. Supervision: S.A., L.W., J.H. Funding Acquisition: L.W., J.H. Writing - Original Draft Preparation: S.A. Writing - Review & Editing: all authors.

References

1. Hie B, Peters J, Nyquist SK, Shalek AK, Berger B, Bryson BD. Computational Methods for Single-Cell RNA Sequencing. Annual Review of Biomedical Data Science 2020 2020/08/03;3(1):339–364. https://doi.org/10.1146/annurev-biodatasci-012220-100601.
2. van der Maaten LJP, Hinton GE. Visualizing High-Dimensional Data Using t-SNE. Journal of Machine Learning Research 2008;9:2579–2625.
3. Carreira-Perpiñán MÁ. The Elastic Embedding Algorithm for Dimensionality Reduction. In: Proceedings of the 27th International Conference on Machine Learning (ICML-10), June 21-24, 2010, Haifa, Israel; 2010. p. 167–174. http://www.icml2010.org/papers/123.pdf.
4. Chen Z, An S, Bai X, Gong F, Ma L, Wan L. DensityPath: an algorithm to visualize and reconstruct cell state-transition path on density landscape for single-cell RNA sequencing data. Bioinformatics 2019 4;35(15):2593–2601.
5. Nguyen LH, Holmes S. Ten quick tips for effective dimensionality reduction. PLOS Computational Biology 2019 06;15(6):1–19. https://doi.org/10.1371/journal.pcbi.1006907.
6. McInnes L, Healy J, Melville J. UMAP: Uniform Manifold Approximation and Projection for Dimension Reduction. arXiv e-prints 2018 Feb;p. arXiv:1802.03426.
7. Becht E, McInnes L, Healy J, Dutertre CA, Kwok IWH, Ng LG, et al. Dimensionality reduction for visualizing single-cell data using UMAP. Nature Biotechnology 2019;37(1):38–44. https://doi.org/10.1038/nbt.4314.
8. Kobak D, Linderman GC. UMAP does not preserve global structure any better than t-SNE when using the same initialization. bioRxiv 2019;https://www.biorxiv.org/content/early/2019/12/19/2019.12.19.877522.
9. Wasserman L. Topological Data Analysis. Annual Review

of Statistics and Its Application 2018;5(1):501–532. https://doi.org/10.1146/annurev-statistics-031017-100045.

10. An S, Ma L, Wan L. TSEE: an elastic embedding method to visualize the dynamic gene expression patterns of time series single-cell RNA sequencing data. BMC Genomics 2019;20(2):224. https://doi.org/10.1186/s12864-019-5477-8.
11. Vladymyrov M, Carreira-Perpinan M. Entropic Affinities: Properties and Efficient Numerical Computation. In: Dasgupta S, McAllester D, editors. Proceedings of the 30th International Conference on Machine Learning, vol. 28 of Proceedings of Machine Learning Research Atlanta, Georgia, USA: PMLR; 2013. p. 477–485. http://proceedings.mlr.press/v28/vladymyrov13.html.
12. Vladymyrov M, Carreira-Perpinan M. Partial-Hessian Strategies for Fast Learning of Nonlinear Embeddings. arXiv e-prints 2012 Jun;p. arXiv:1206.4646.
13. Balay S, Abhyankar S, Adams MF, Brown J, Brune P, Buschelman K, et al., PETSc Web page; 2019. https://www.mcs.anl.gov/petsc. https://www.mcs.anl.gov/petsc.
14. Balay S, Abhyankar S, Adams MF, Brown J, Brune P, Buschelman K, et al. PETSc Users Manual. Argonne National Laboratory; 2019.
15. Balay S, Gropp WD, McInnes LC, Smith BF. Efficient Management of Parallelism in Object Oriented Numerical Software Libraries. In: Arge E, Bruaset AM, Langtangen HP, editors. Modern Software Tools in Scientific Computing Birkhäuser Press; 1997. p. 163–202.
16. Moon KR, van Dijk D, Wang Z, Gigante S, Burkhardt DB, Chen WS, et al. Visualizing structure and transitions in high-dimensional biological data. Nature Biotechnology 2019;37(12):1482–1492. https://doi.org/10.1038/s41587-019-0336-3.
17. Setty M, Tadmor MD, Reich-Zeliger S, Angel O, Salame TM, Kathail P, et al. Wishbone identifies bifurcating developmental trajectories from single-cell data. Nature Biotechnology 2016;34(6):637–645. https://doi.org/10.1038/nbt.3569.
18. Butler A, Hoffman P, Smibert P, Papalexi E, Satija R. Integrating single-cell transcriptomic data across different conditions, technologies, and species. Nature Biotechnology 2018;36(5):411–420. https://doi.org/10.1038/nbt.4096.
19. Stuart T, Butler A, Hoffman P, Hafemeister C, Papalexi E, Mauck I William M, et al. Comprehensive Integration of Single-Cell Data. Cell 2019 2020/08/03;177(7):1888–1902.e21. https://doi.org/10.1016/j.cell.2019.05.031.
20. Schiebinger G, Shu J, Tabaka M, Cleary B, Subramanian V, Solomon A, et al. Optimal-Transport Analysis of Single-Cell Gene Expression Identifies Developmental Trajectories in Reprogramming. Cell 2019 2020/02/05;176(4):928–943.e22. https://doi.org/10.1016/j.cell.2019.01.006.
21. Seo E, Basu-Roy U, Gunaratne PH, Coarfa C, Lim DS, Basilico C, et al. SOX2 Regulates YAP1 to Maintain Stemness and Determine Cell Fate in the Osteo-Adipo Lineage. Cell Reports 2013;3(6):2075–2087. http://www.sciencedirect.com/science/article/pii/S2211124713002465.
22. Hanieh H, Ahmed EA, Vishnubalaji R, Alajez NM. SOX4: Epigenetic regulation and role in tumorigenesis. Seminars in Cancer Biology 2019;http://www.sciencedirect.com/science/article/pii/S1044579X18301809.
23. Yu P, Nie Q, Tang C, Zhang L. Nanog induced intermediate state in regulating stem cell differentiation and reprogramming. BMC Systems Biology 2018;12(1):22. https://doi.org/10.1186/s12918-018-0552-3.

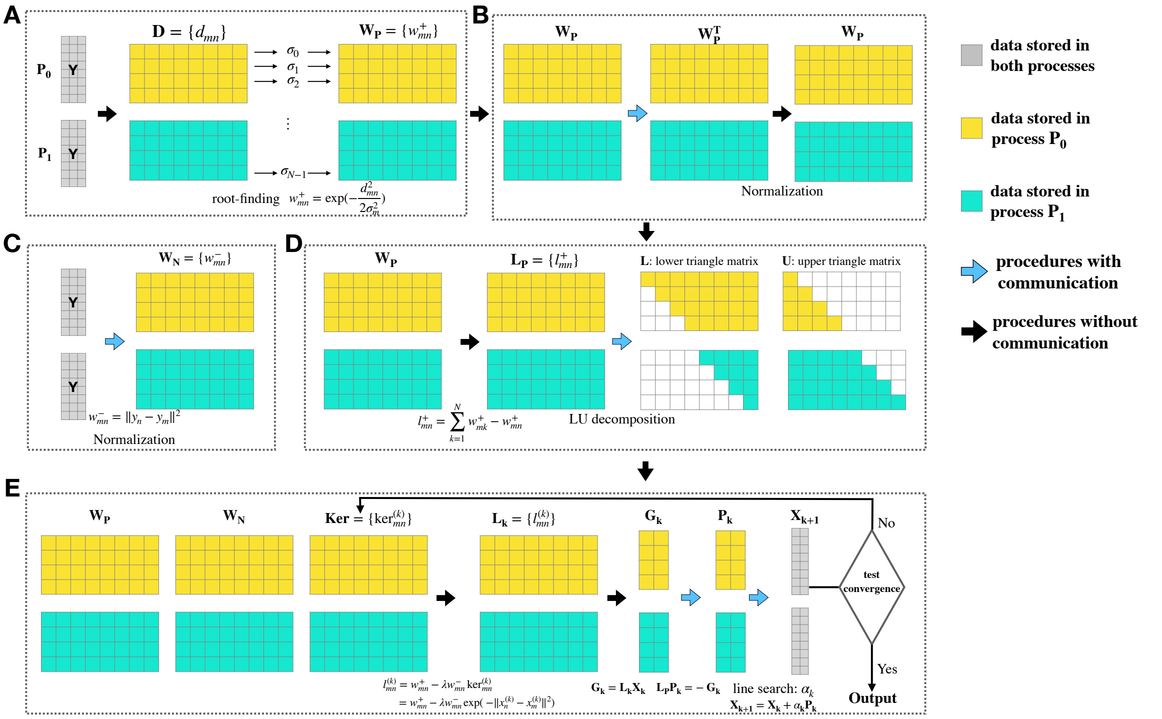

Figure 1. Overview of D-EE algorithm. The D-EE algorithm can be decomposed into five parts. Part A: the computation of matrixes $\mathbf{D}$ and $\mathbf{W_P}$. To obtain $\mathbf{W_P}$, the parameters $\{\sigma_n\}$ are determined by solving a series of root-finding problems. Part B: the symmetry and normalization of the matrix $\mathbf{W_P}$. Part C: the computation and normalization of $\mathbf{W_N}$. Part D: the computation of the Laplacian matrix $\mathbf{L_P}$ together with LU decomposition. Part E: the computation of $\mathbf{X_{k+1}}$ by solving an optimization problem with the classic Quasi-Newton methods iteratively. In the *k-th* iteration, $\mathbf{L_k}$ is computed based on $\mathbf{W_P}$, $\mathbf{W_N}$ and $\mathbf{X_k}$, which are used to obtain the gradient $\mathbf{G_k} = \mathbf{L_k X_k}$. The descent direction $\mathbf{P_k}$ is then determined by solving a linear system $\mathbf{L_P P_k} = -\mathbf{G_k}$. Finally, $\mathbf{X_{k+1}}$ is updated according to $\mathbf{X_{k+1}} = \mathbf{X_k} + \alpha_k \mathbf{P_k}$, where the step size α_k is calculated by a line search method.

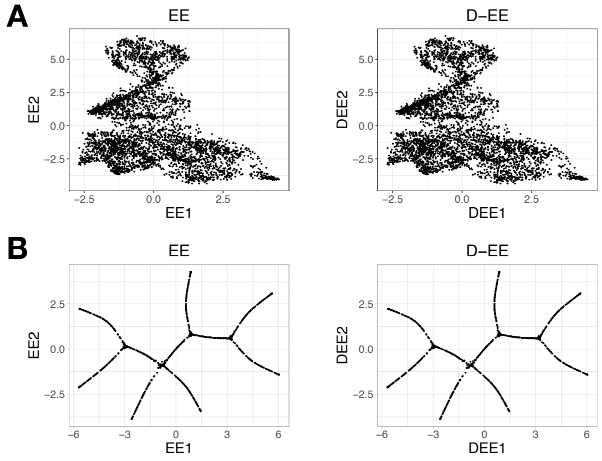

Figure 2. D-EE and EE achieve the same results on two datasets when using the same initial conditions. A: The 2-D mapping of HSPCs data obtained by the two algorithms. B: The 2-D mapping of PHATE data obtained by the two algorithms.

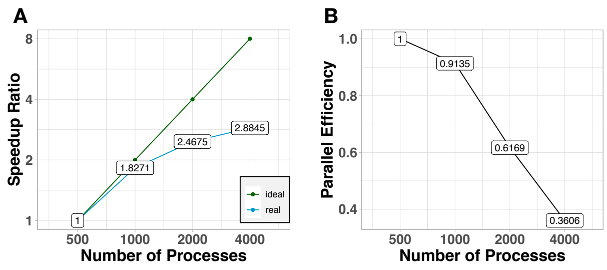

Figure 3. Strong scaling results and parallel efficiency of the D-EE algorithm on an LSSC-IV supercomputer. We apply D-EE on iPSCs dataset by using 500, 1,000, 2,000, and 4,000 processes, respectively. A: The strong speedup ratio increases with increase in the number of processes. B: The parallel efficiency decreases at an acceptable rate with the increase in number of processes.

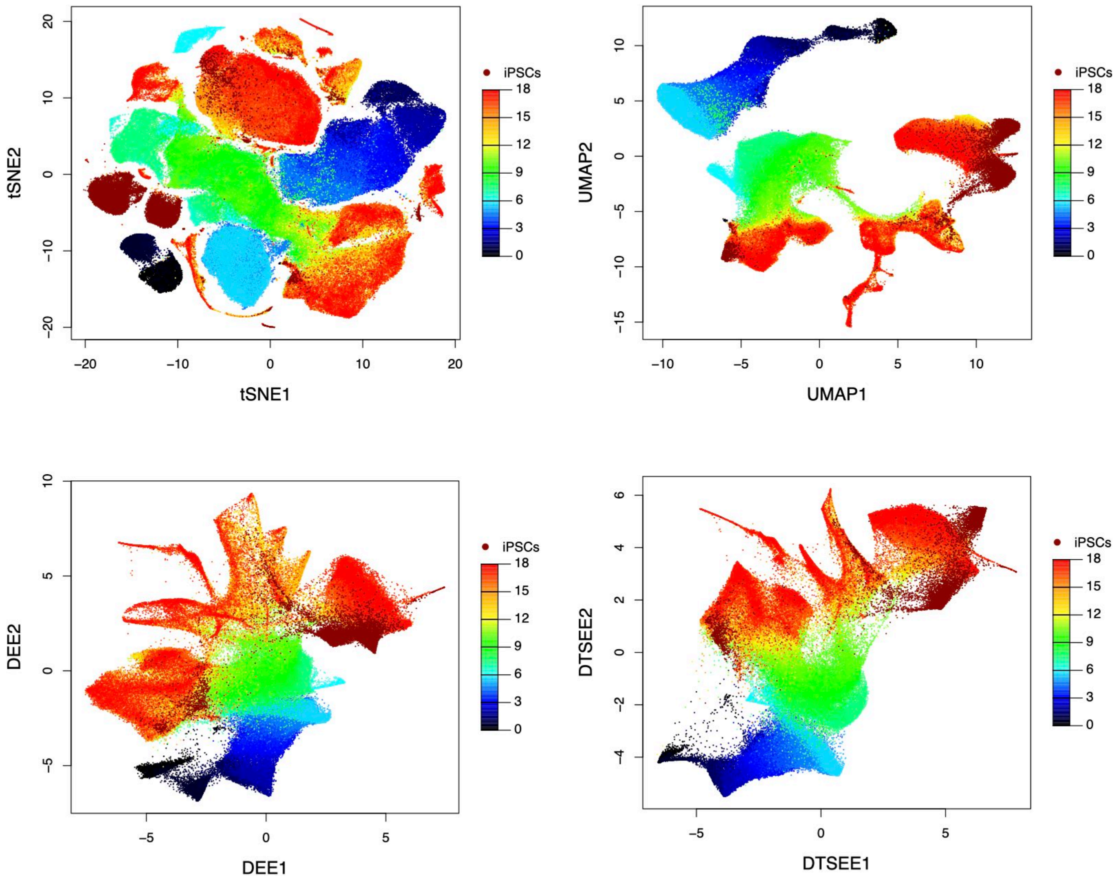

Figure 4. Cells are colored by time points in iPSCs dataset on the 2-dimensional space obtained by four dimensionality reduction methods of t-SNE, UMAP, D-EE and D-TSEE, respectively.

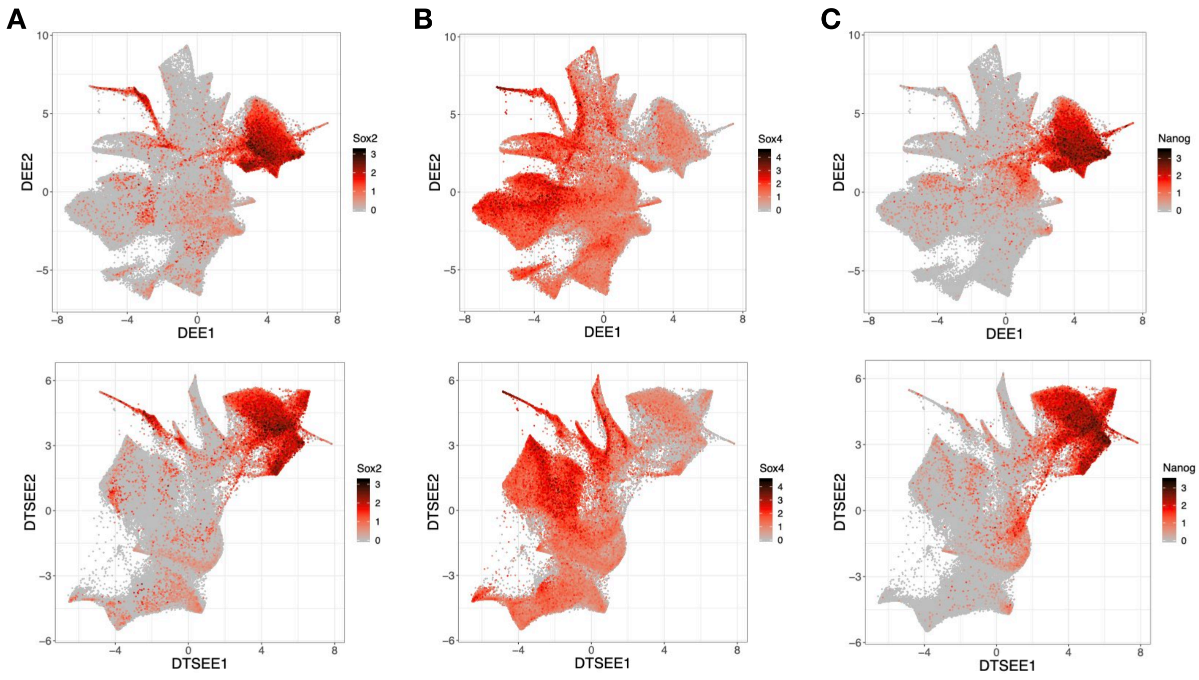

Figure 5. Cells are colored by gene expression of Sox2, Sox4, and Nanog in iPSCs dataset on the 2-dimensional embedding obtained by D-EE and D-TSEE, respectively.

Figure

Click here to access/download;Figure;2 ipsc_plot_time.pdf

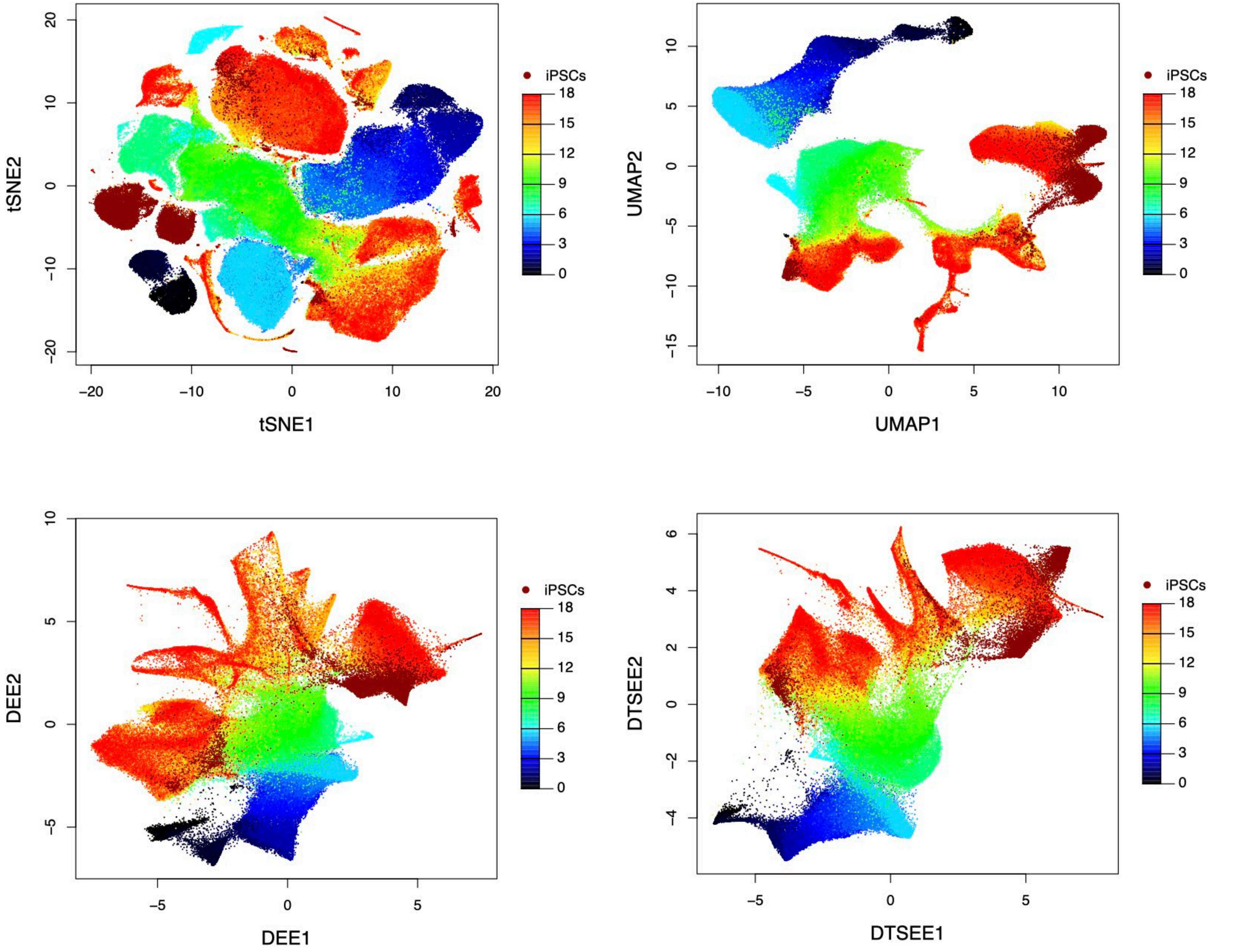

Figure

Click here to access/download;Figure;3 ipsc_gene_heatmap.pdf

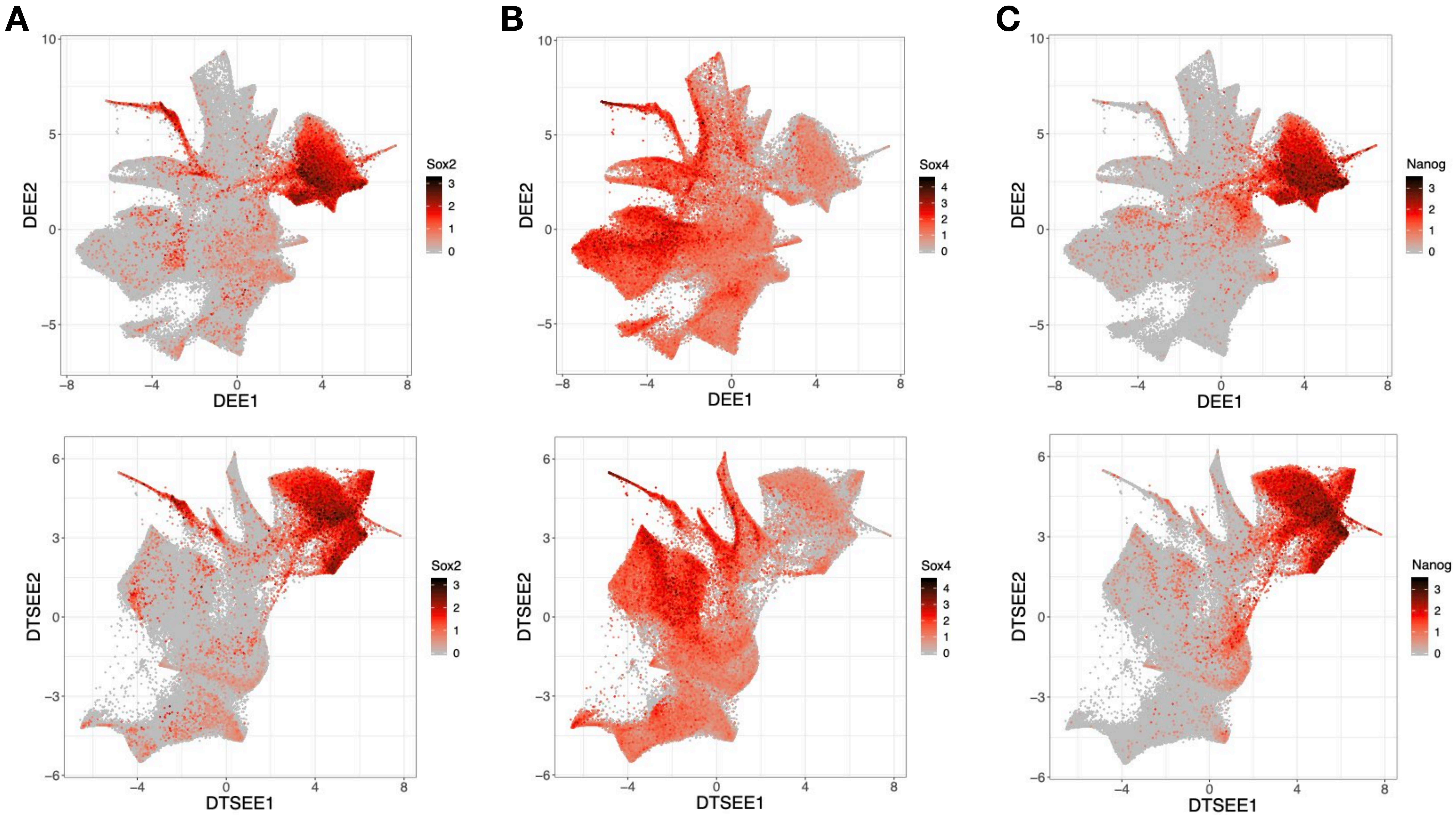

Figure
Click here to access/download;Figure;4 strong_scaling.pdf

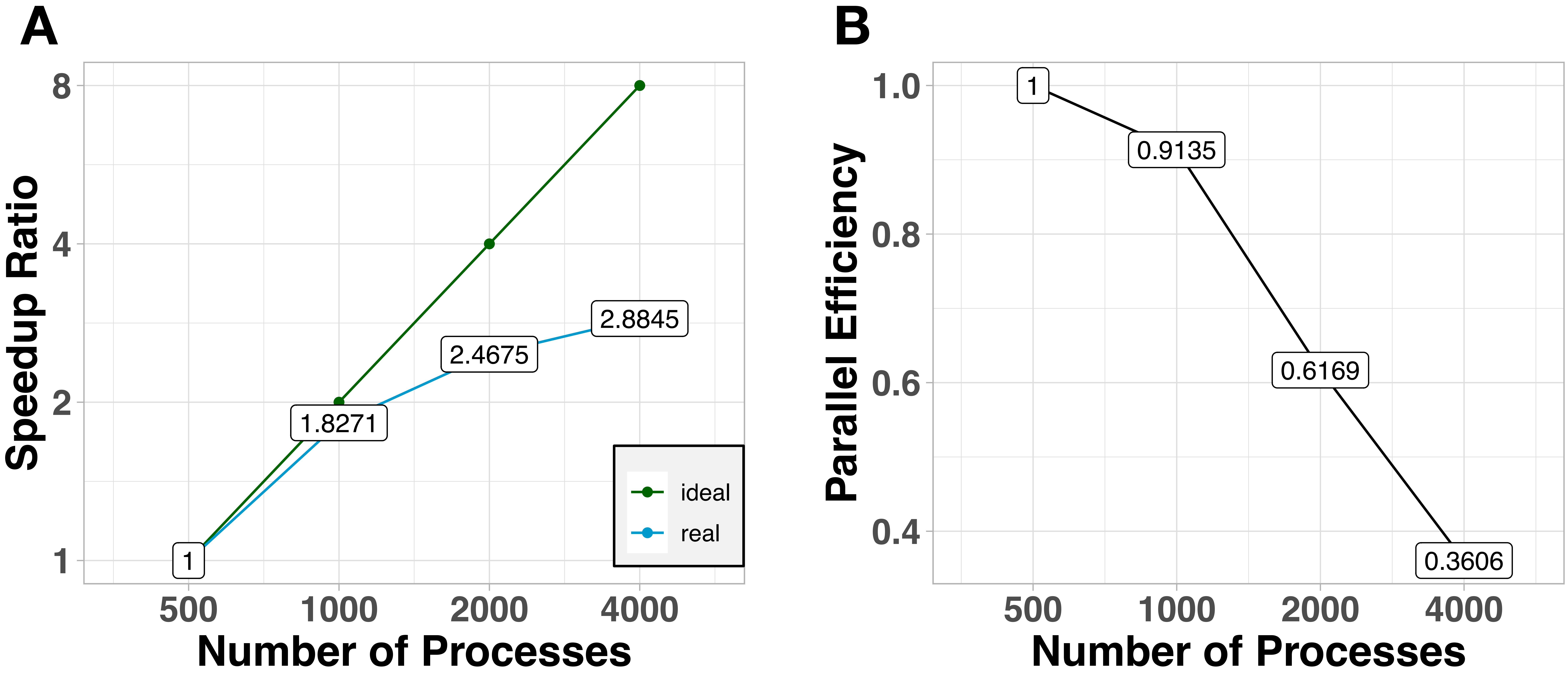

Figure
Click here to access/download;Figure;5 hpscs_phate_seqee_dee.pdf

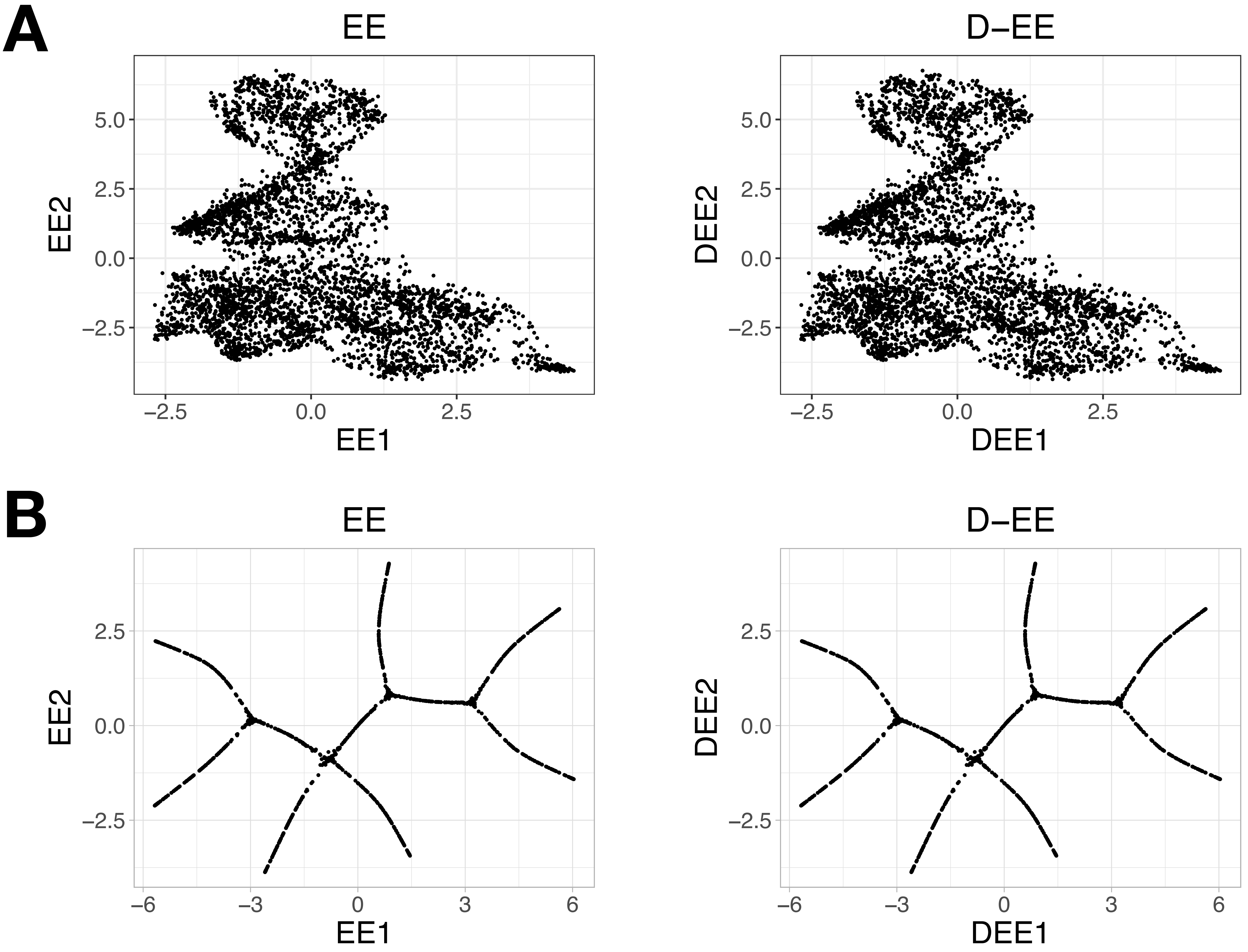

Figure
Click here to access/download;Figure;6 parallel-ee.pdf

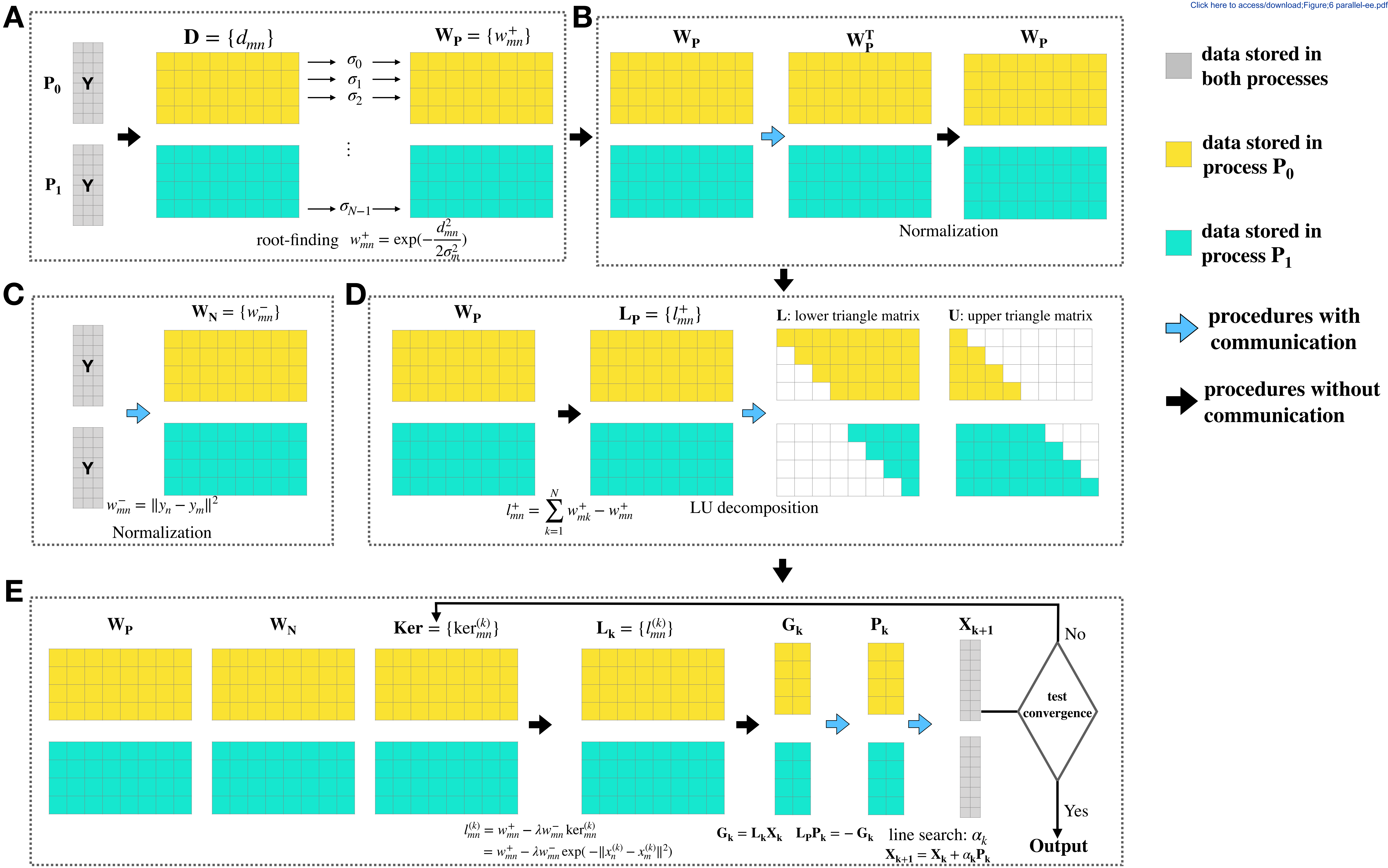

Cover letter

Click here to access/download
Supplementary Material
8 cover letter.docx
