# Supplementary material for: D-EE: Distributed software for visualizing intrinsic structure of large-scale single-cell data
Source: Gigascience. 2020 Nov 11;9(11):giaa126. doi: 10.1093/gigascience/giaa126 (PMC7657844; doi:10.1093/gigascience/giaa126)
Supplement: giaa126_GIGA-D-20-00236_Revision_1 [file giaa126_giga-d-20-00236_revision_1.pdf]

GigaScience

D-EE: a distributed software for visualizing intrinsic structure of large-scale single-cell data

--Manuscript Draft--

**Manuscript Number:**	GIGA-D-20-00236R1
**Full Title:**	D-EE: a distributed software for visualizing intrinsic structure of large-scale single-cell data
**Article Type:**	Technical Note
**Funding Information:**	National Key R&D Program of China (2018YFB0704304) — Dr. Lin Wan<br>NSFC (11871069) — Dr. Jizu Huang<br>NSFC (12071466) — Dr. Lin Wan
**Abstract:**	Background: Dimensionality reduction and visualization play vital roles in single-cell RNA sequencing (scRNA-seq) data analysis. While they have been extensively studied, state-of-the-art dimensionality reduction algorithms are often unable to preserve the global structures underlying data. Elastic Embedding (EE), a nonlinear dimensionality reduction method, has shown promise in revealing low-dimensional intrinsic local and global data structure. However, the current implementation of the EE algorithm lacks scalability to large-scale scRNA-seq data.<br><br>Results: We present a distributed optimization implementation of the EE algorithm, termed distributed Elastic Embedding (D-EE). D-EE reveals the low-dimensional intrinsic structures of data with accuracy equal to that of Elastic Embedding, and it is scalable to large-scale scRNA-seq data. It leverages distributed storage and distributed computation, achieving memory efficiency and high-performance computing simultaneously. In addition, an extended version of D-EE, termed distributed optimization implementation of time series Elastic Embedding (D-TSEE), enables the user to visualize large-scale time series scRNA-seq data by incorporating experimental temporal information. Results with a large-scale scRNA-seq data indicate D-TSEE can uncover oscillatory gene expression patterns by employing experimentally temporal information.<br><br>Conclusions: D-EE is a distributed dimensionality reduction and visualization tool. Its distributed storage and distributed computation technique allow us to efficiently analyze large-scale single-cell data at the cost of constant time speedup. The source code for D-EE algorithm based on C and MPI tailored to a High Performance Computing cluster is available at https://github.com/ShaokunAn/D-EE.
**Corresponding Author:**	Lin Wan<br>Academy of Mathematics and Systems Science, Chinese Academy of Sciences<br>Beijing, CHINA
**Corresponding Author Secondary Information:**	
**Corresponding Author's Institution:**	Academy of Mathematics and Systems Science, Chinese Academy of Sciences
**Corresponding Author's Secondary Institution:**	
**First Author:**	Shaokun An
**First Author Secondary Information:**	
**Order of Authors:**	Shaokun An<br>Jizu Huang<br>Lin Wan
**Order of Authors Secondary Information:**	

Powered by Editorial Manager® and ProduXion Manager® from Aries Systems Corporation

Response to Reviewers:

Response to reviewers:

Reply:

We would like to thank the reviewers for thoughtful comments and valuable suggestions on our manuscript. The manuscript has been fully revised by following the reviewers' comments and suggestions, making our results more solid and convincing. We hope the new version will meet the criteria of GigaScience. The point-by-point responses to the reviewers' comments and suggestions are as follows.

Reviewer #1:

Does the manuscript adhere to the journal's guidelines on minimum standards of reporting?
Yes
General comments: The authors propose a distributed modification to Elastic Embedding (EE) approaches that provide a multiprocessing speedup to visualization of scRNA-seq data. The manuscript is well written, is based on sound techniques, and describes the overall procedure well. There are some places in which the authors do overclaim or omit important information, so the manuscript would benefit from repositioning the text of the paper accordingly. Overall, I am supportive of publication of the manuscript.

Reply:

We thank the reviewer for summarizing our work and also the thoughtful comments. We give the point-to-point response below.

Comment:
1) Are the methods appropriate to the aims of the study, are they well described, and are necessary controls included?

The math and methodological description of the method is done clearly, which is very much appreciated. Providing GitHub code with instructions for reproducibility is also great.

Reply:

We thank the reviewer for the thoughtful comments.

Comment:
2) Are the conclusions adequately supported by the data shown?

There are some areas in which the manuscript makes claims that should be toned down. Or, if the authors wish to make these claims, then they would need additional analytic support.

The paper claims that EE "outperforms t-SNE and UMAP" in preserving global structures. Such a claim would need to be supported by a much more exhaustive consideration of different visualization parameters. In particular, for UMAP, I might suspect that with different parameters (e.g., a higher nearest neighbor parameter), the "global structure" would not be broken. Instead, the authors could just say something about how well their own method works without the relative comparison, and still provide single instances of t-SNE and UMAP for a visual comparison.

Reply:

Thanks for your thoughtful comments. In the revised manuscript, we followed your advice and toned down the claims as follows.

(1) We removed all the claims that "EE outperforms t-SNE and UMAP" in the previous version, and merely provided their visual comparisons on iPSCs data (see the second paragraph of subsection "D-EE and D-TSEE recover intrinsic low-dimensional structures of large-scale scRNA-seq data" on Page 5 and Figure 4).

(2) We followed your suggestion and tuned the parameters of t-SNE and UMAP. We found that their performances can be further improved, especially for t-SNE. A short paragraph was added in the revised manuscript as follows. "…The t-SNE is conducted by the FIt-SNE method [23] with a PCA initialization, and we choose the

Powered by Editorial Manager® and ProduXion Manager® from Aries Systems Corporation

learning rate as 1/12 of the sample size, according to [3] for better preservation of the global structures. UMAP is conducted by adjusting the number of nearest neighbors (NNs) to balance the preservation of local and global structures. We find that UMAP is not sensitive when choosing the number of NNs from 30 (defaults) to 500 (square root of the number of samples), and we thus set the number of NNs to be 100 in our study. Both t-SNE and UMAP are implemented by the Seurat software (version 3.2.1)..." (see the first paragraph of subsection "D-EE and D-TSEE recover intrinsic low-dimensional structures of large-scale scRNA-seq data" on Page 5.)

Comment:
The authors should also make crystal clear that the speedup is effectively only a constant time one, which may be limiting since implementations of t-SNE (e.g., Barnes-Hut t-SNE or flt-SNE) and UMAP can run in close-to-linear time, which is a big practical win for those methods. In contrast, O(N^2/P) is still large when N = 2 million (e.g., the dataset in Cao et al., Nature, 2019). There's also no speed comparison between distributed EE and, e.g., UMAP. The authors need to make this point clear.

Reply:
Thanks for pointing out this issue. We agree well with you that the implementation of D-EE can be limited due to the constant time speedup.
In the revised manuscript:
Firstly, we made crystal discussion of this issue as: "We demonstrate that D-EE and D-TSEE work efficiently on large-scale datasets at a super computer. However, the proposed distributed algorithm D-EE still has disadvantage due to the huge computational cost and storage with a relative large number of cells. Therefore, D-EE is limited to handle and analyze huge-scale datasets with the number of cells up to the order of millions [27]. In comparison, the state-of-the-art accelerated implementations of t-SNE (e.g., FIt-SNE) and UMAP are of the close-to-linear computational complexities, showing great efficiency on huge data analysis." (see the second paragraph of Conclusion on Page 5)
Secondly, we pointed out that we can further improve D-EE to handle huge-scale datasets as follows. "In the future study, to resolve the limitation of D-EE on huge-scale data computation, we can accelerate D-EE by adopting either the fast Fourier transform as used in FIt-SNE, or adopting the state-of-the-art neural network framework used by net-SNE [28]. On the other hand, since huge-scale single-cell dataset can be highly redundant, we can also select subset of informative samples using the advanced geometric sketching tool [29] prior to application of D-EE." (see the last paragraph of Conclusion on Page 5)
Thirdly, we explored the relationship of runtime consumptions versus the sample sizes, showed in Figure 3C, and described the results as "We further evaluate the performance of D-EE on computational times in our supercomputer. Three test cases with sample sizes being 10k, 50k, and 100k are run on 500, 1,000, and 2,000 processors, respectively. It is shown that when using the same number of processes, it is naturally that the computational time increases as total sample size increases (as shown in Figure 3C). " (see the first paragraph on Page 5).

Comment:
3) Please indicate the quality of language in the manuscript. Does it require a heavy editing for language and clarity?

Language quality is good. One minor nitpick is to avoid contractions, e.g., "it's," in formal writing.

Reply:
Thanks for your pointing out this. We have corrected these contractions in the revised manuscript.

Comment:
4) Are you able to assess all statistics in the manuscript, including the appropriateness of statistical tests used?

Yes

Powered by Editorial Manager® and ProduXion Manager® from Aries Systems Corporation

Reviewer #2:
The paper by An et al. presents a distributed implementation of the elastic embedding (EE) algorithm that allows to run EE on large datasets. The authors argue that EE (and its time-series version TSEE, previously developed by the same authors) is a good choice of an embedding algorithm for single-cell data, and so their implementation allows to actually use it in practice. Previously existing implementations did not scale beyond a sample size of a few thousand cells.

This is a solid work on implementing a parallelized/distributed version of EE. The EE/TSEE algorithms can indeed be an attractive choice of an embedding algorithm for single-cell data, so this paper makes a contribution that is relevant for the field. Importantly, this paper does *not* suggest any new algorithm, but simply presents an efficient implementation of an existing algorithm. If this is in scope of the "Technical note" section of GigaScience, then I can recommend acceptance.

Reply:
Thanks for your thoughtful comments. We followed the instructions of GigaScience and submitted our manuscript to “Technical note” as our target, since “Technical Notes should present an open-source software tool or an experimental or computational method, test or procedure for the analysis or handling of large-scale data.”

Comment:
The main issue I have is that it seems the authors used a supercomputer to run their analysis, and it is not clear to me if this implementation would allow to analyze the same dataset (n=250k) on a normal desktop computer. For competitor algorithms like t-SNE and UMAP this dataset size is not a problem even on a laptop. If D-EE can only deal with this dataset on a supercomputer, then it's clearly much much less efficient than competitors. The authors should clarify this point, see also below.

MAJOR COMMENTS

* Even though the main contribution of the paper is efficient EE implementation, the authors do not provide almost any benchmarking results. What I would like to see reported in the text:

a)What exact hardware was used for the experiments? On page 4 the authors say that they used up to 4000 CPU processes. What kind of computer allows to run 4000 processes? Was it a computing cluster? Please give exact details of the used hardware. Caption of Figure 3 says it was a supercomputer, but more details should be given in the text.

Reply:
Thanks for your thoughtful comments. In the revised manuscript, we provided detailed information of the computing cluster on which we tested and implemented our software in main text as follows: “… The numerical tests are carried out on the LSSC-IV supercomputer. The 400 computing nodes of LSSC-IV are comprised of two 18-core Intel Xeon Gold CPUs with 192 GB local memory, and are interconnected via a proprietary high performance network. ” (see the first paragraph of “D-EE achieves high strong scaling efficiency” on Page 4)

Comment:
b) What was the actual runtime on this supercomputer? At a minimum, report the physical time for iPSCs dataset in the text (e.g. 1 hour, or 20 hours, or 30 minutes). Even better would be a figure (perhaps another panel in Figure 3) that plots the time as a function of sample size. What is the largest sample size that can be feasibly analyzed on the supercomputer?

Reply:
Thank you for your suggestion.
In the revised manuscript:

Powered by Editorial Manager® and ProduXion Manager® from Aries Systems Corporation

Firstly, we tested our software extensively at various numbers of processes on iPSCs dataset and provided the detailed runtime information: “To test performance of parallel efficiency of D-EE on the large-scale dataset, we apply D-EE to the iPSCs dataset (~ 250k cells) using 500, 1,000, 2,000, and 4,000 processes, respectively, and for each setting of number of processes we run at least twice. The averaged computation times of D-EE for iPSCs dataset are 5.83 hours, 3.19 hours, 2.36 hours, and 2.02 hours, when the number of processes are 500, 1,000, 2,000, and 4,000, respectively...” (see subsection “D-EE achieves high strong scaling efficiency” on Page 4)

Secondly, we explored the relationship of runtime versus the sample sizes, displayed in Figure 3C, and described the results as “We further evaluate the performance of D-EE on computational times in our supercomputer. Three test cases with sample sizes being 10k, 50k, and 100k are run on 500, 1,000, and 2,000 processors, respectively. It is shown that when using the same number of processes, it is naturally that the computational time increases as total sample size increases (as shown in Figure 3C).” (see the first paragraph on Page 5)

Thirdly, we estimated the largest sample size that can be feasibly analyzed on the LSSC-IV supercomputer on which we implemented our software. Specifically, for a dataset with 10k samples implemented by using 8 processes, it consumed about 10 Gb memory. According to the memory of our supercomputer and the square relationship between the memory cost and the sample size N, we estimated the largest sample size that can be analyzed on our supercomputer is about 850k. We provided this information in the Instruction of D-EE at GitHub https://github.com/ShaokunAn/D-EE.

Comment:
c) Most importantly -- most users will not have access to a supercomputer (!!). The text should clearly state how large a dataset can be feasibly analyzed on a typical desktop computer (8 CPU threads, 16 Gb RAM) and perhaps on a typical powerful lab computing station (e.g. 40 CPU threads, 256 Gb RAM). Maybe include the runtimes using such hardware into the figure that I suggested above.

Reply:
Thanks for pointing out this problem. In the revised manuscript, we made it clear that a large computing cluster is recommended for the implementation of D-EE when analyzing large-scale datasets, and provided information about the largest sample size that can be feasibly analyzed on the two typical computing devices as follows: “In practice, for analysis of large-scale single-cell dataset, a workstation with multiple CPUs and large memory is recommended. Meanwhile, we also test D-EE on datasets at different sample sizes, and find that D-EE efficiently implements on a typical personal computer (e.g., 8 CPU threads, 16 Gb RAM) with up to a sample size of 12k, while on a conventional workstation (e.g., 40 CPU threads, 256 Gb RAM) with up to a sample size of 48k, respectively.” (see the second paragraph on Page 5)

For large sample size up to 250k as shown in our cases, D-EE has to be implemented on a super computer due to the large memory consumption. As mentioned in the revised manuscript, the improvement of D-EE is discussed as a future work: “In the future study, to resolve the limitation of D-EE on huge-scale data computation, we can accelerate D-EE by adopting either the fast Fourier transform as used in FIt-SNE, or adopting the state-of-the-art neural network framework used by net-SNE [28]. On the other hand, since huge-scale single-cell dataset can be highly redundant, we can also select subset of informative samples using the advanced geometric sketching tool [29] prior to application of D-EE.” (see the last paragraph of Conclusion section on Page 5)

Comment:
d) In case this implementation only works well on a supercomputer, and is not very helpful on a personal desktop computer, this should be stated very prominently in the abstract/intro/discussion/etc. Currently the abstract says that D-EE allows "to efficiently" analyze large RNAseq data. But if I must have a supercomputer for such an analysis, then it's not really very "efficient". The abstract should be clear about this. Note that t-SNE and UMAP can easily analyze n=250k on a desktop computer or a laptop. The authors have to discuss this in the Discussion.

Powered by Editorial Manager® and ProduXion Manager® from Aries Systems Corporation

Reply:
Thanks for your valuable suggestion. In the revised manuscript, we made it crystal clear in abstract that "…Its distributed storage and distributed computation technique allow us to efficiently analyze large-scale single-cell data at the cost of constant time speedup."
We also discussed the limitation of D-EE in revised manuscript as "We demonstrate that D-EE and D-TSEE work efficiently on large-scale datasets at a super computer. However, the proposed distributed algorithm D-EE still has disadvantage due to the huge computational cost and storage with a relative large number of cells. Therefore, D-EE is limited to handle and analyze huge-scale datasets with the number of cells up to the order of millions [27]. In comparison, the state-of-the-art accelerated implementations of t-SNE (e.g., FIt-SNE) and UMAP are of the close-to-linear computational complexities, showing great efficiency on huge data analysis." (see the second paragraph of Conclusion on Page 5).
Finally, in revised manuscript, we discussed potential improvement of D-EE for computation of huge datasets: "In the future study, to resolve the limitation of D-EE on huge-scale data computation, we can accelerate D-EE by adopting either the fast Fourier transform as used in FIt-SNE, or adopting the state-of-the-art neural network framework used by net-SNE [28]. On the other hand, since huge-scale single-cell dataset can be highly redundant, we can also select subset of informative samples using the advanced geometric sketching tool [29] prior to application of D-EE." (see the last paragraph of Conclusion section on Page 5)

Comment:
* The authors need to give exact details of how they ran t-SNE and UMAP for Figure 4 (page 4). Was it default parameters of some implementation? Name the implementation, give the version, say that it was default parameters. If you changed some parameters from the default ones, specify all of them.

In particular, for t-SNE I am worried that a random initialization was used for Figure 4. Random initialization is a bad idea, as stated e.g. in the cited Ref [8]. UMAP uses non-random init, so this is not a fair comparison. I suggest to use PCA initialization as explained in Ref [8] and also as is default in the latest versions of FIt-SNE and openTSNE.

Apart from that, t-SNE is sensitive to the learning rate (see https://www.nature.com/articles/s41467-019-13056-x), so it should be set appropriately, e.g. to n/12, which is also the default in FIt-SNE and openTSNE.

Reply:
Thank you for your valuable suggestion. In revised manuscript, we followed the instruction of the reference you provided, and tuned the parameters of both t-SNE and UMAP, and found that the performance of t-SNE can be largely improved. We added detailed implementation of t-SNE and UMAP as well as their version as follows: "The t-SNE is conducted by the FIt-SNE method [23] with a PCA initialization, and we choose the learning rate as 1/12 of the sample size, according to [3] for better preservation of the global structures. UMAP is conducted by adjusting the number of nearest neighbors (NNs) to balance the preservation of local and global structures. We find that UMAP is not sensitive when choosing the number of NNs from 30 (defaults) to 500 (square root of the number of samples), and we thus set the number of NNs to be 100 in our study. Both t-SNE and UMAP are implemented by the Seurat software (version 3.2.1). In our implementations, both D-EE and D-TSEE are used with their default parameters." (see the first paragraph of subsection "D-EE and D-TSEE recover intrinsic low-dimensional structures of large-scale scRNA-seq data" on Page 5)

Comment:
* page 1 -- You might want to consider citing https://www.nature.com/articles/s41467-019-13056-x after "widely used in the single-cell community".

Reply:
Thanks for pointing out this valuable reference. In our revised manuscript, we cited

the reference in 3 places:

(1) Introduction: "…For example, the celebrated t-distributed Stochastic Neighbor Embedding (t-SNE) algorithm [2] is widely used in the single-cell community [3]." (see Page 1)

(2) Methods: "…It is worth to note that, a newly proposed combinational perplexity has been applied to t-SNE [3], which greatly enhances the performance of t-SNE in preservation of global structures. The combinational perplexity can be also adopted by D-EE in future update." (see Page 2)

(3) Results: "…The t-SNE is conducted by the FIt-SNE method [23] with a PCA initialization, and we choose the learning rate as 1/12 of the sample size, according to [3] for better preservation of the global structures." (see Page 5)

Comment:
* page 2 -- "EE extends t-SNE", not sure this is a good way to put it. EE is not really an "extension" of t-SNE, is it? It's a different but very related algorithm. Consider revising.

Reply:

Thanks for your suggestion. In the revised manuscript, we modified the sentence as "…To achieve this goal, EE penalizes the placement of latent points in close proximity away from dissimilar data points in high-dimensional space…" (see Introduction on Page 2)

Comment:
•page 2, a bit later -- "the current implementations ... is" -> are

Reply:

Thanks for pointing this issue. We have corrected these typos in revised manuscript.

Comment:
•page 2, after the first formula: as far as I know, the original EE paper used one fixed value of sigma (kernel width) and not adaptive sigma_n as you do here. I suggest to mention this and explain/cite where the sigma_n comes from. Looks like it's from Ref [11] (last sentence in that paragraph). If so, write that you follow [11] in using adaptive sigma_n based on perplexity (?), and you use perplexity=X by default.

Reply:

Thanks for your valuable suggestion. In the revised manuscript, we provided a detailed description of parameters σ_n as well as the perplexity parameter (defaults as 20) as follows:

"…The σ_m in $w_{mn}^+$is a sample-specific scaling parameter. It is estimated adaptively by solving a sample-specific root-finding problem, such that the sample-specific distribution over its neighbors having a desired perplexity (see [12] for details). We set a default value of perplexity as 20 in this study." (see Methods on Page 2)

We also pointed out that, "…It is worth to note that, a newly proposed combinational perplexity has been applied to t-SNE [3], which greatly enhances the performance of t-SNE in preservation of global structures. The combinational perplexity can be also adopted by D-EE in future update." (see Methods on Page 2)

Comment:
•page 4, last sentence of "Data description": this is not enough detail. Logarithmic means log(x+1)? How exactly did you find variable features? How many? Please provide full description and cite some literature where you took the recommendations from. If you used a particular software to do it, say so and cite.

Reply:

In the revised manuscript, we described the detailed information on data pre-process as follows

"Firstly, we normalize the gene expression in each sample as follows: we divide each gene read count by the total read counts for each cell, and then multiply a scale

Powered by Editorial Manager® and ProduXion Manager® from Aries Systems Corporation

factor of 10^4 and plus one, followed by taking a logarithmic transformation. Secondly, we select the top 2,000 variable genes using the default “vst” method of Seurat package, i.e., variance-stabilizing transformation [21].” (see the second paragraph of Results on Page 4)

The package used to perform pre-process is specified as “The obtained single-cell read count data is pre-processed by Seurat package (version 3.2.1) [19, 20].” (see the second paragraph of Results on Page 4)

Comment:
•page 4: "lambda used is set to the default values" -- what values are default? How can the reader know that? Please specify exact values of all parameters.

Reply:
Thanks for pointing out this issue. In the revised manuscript, we added the default value of parameter lambda as: “In single-cell data analysis, with $\lambda = 10$, EE can achieve robust performance with high accuracy [5]. Therefore, we set the default value of λ as 10 for D-EE.” (see Methods on Page 2)

Comment:
page 4, description of Figure 2: Frobenius norm only makes sense if you used the same initialization. Please specify in the text that it was the same and also specify what the initialization was (random?).

Reply:
Thanks for your suggestion. In the revised manuscript, we provided the definition of Frobenius norm and illustrated same initialization was used when perform consistency experiments as:
“… We use the same initialization generated from Gaussian distribution as that in the original EE Matlab code… “ (see Results on Page 4)
“…To measure the consistency quantitatively, we calculate the relative error which is defined by
$\text{relative error} = \frac{\|A-B\|_F}{\|A\|_F}$, where $\|\cdot\|_F$ is the Frobenius norm of matrix, A and B represent the output of EE and D-EE, respectively. Frobenius norm of a matrix $A \in R^{m\times n}$ is defined as $\|A\|_F=\sqrt{\sum_{i=1}^m \sum_{j=1}^n |a_{ij}|^2}$.” (see Results on Page 4)

Comment:
•page 5, "oscillatory patterns" -- I am not sure I see the oscillatory patterns in the figure. I suggest the authors highlight it in the figure somehow.

Reply:
Thank you for pointing out this. In revised manuscript, we added labels and marks on Figure 5 to better illustrate the oscillating pattern.

**Additional Information:**	
**Question**	**Response**
Are you submitting this manuscript to a special series or article collection?	No
**Experimental design and statistics**<br><br>Full details of the experimental design and statistical methods used should be given in the Methods section, as detailed in our Minimum Standards Reporting Checklist. Information essential to interpreting the data presented should be made available in the figure legends.	Yes

Powered by Editorial Manager® and ProduXion Manager® from Aries Systems Corporation

Have you included all the information requested in your manuscript?	
**Resources**<br><br>A description of all resources used, including antibodies, cell lines, animals and software tools, with enough information to allow them to be uniquely identified, should be included in the Methods section. Authors are strongly encouraged to cite Research Resource Identifiers (RRIDs) for antibodies, model organisms and tools, where possible.<br><br>Have you included the information requested as detailed in our Minimum Standards Reporting Checklist?	Yes
**Availability of data and materials**<br><br>All datasets and code on which the conclusions of the paper rely must be either included in your submission or deposited in publicly available repositories (where available and ethically appropriate), referencing such data using a unique identifier in the references and in the “Availability of Data and Materials” section of your manuscript.<br><br>Have you have met the above requirement as detailed in our Minimum Standards Reporting Checklist?	Yes

LaTeX - Main Document (TeX file)

Click here to access/download;LaTeX - Main Document (TeX file);main.tex

Placeholder for OUP logo oup.pdf

Placeholder for journal logo gigascience-logo.pdf

GigaScience, 2020, 1–8

doi: xx.xxxx/xxxx
Manuscript in Preparation
Technical Note

TECHNICAL NOTE

D-EE: a distributed software for visualizing intrinsic structure of large-scale single-cell data

Shaokun An[1,2], Jizu Huang[1,2,*] and Lin Wan[1,2,*]

[1]NCMIS, LSEC, LSC, Academy of Mathematics and Systems Science, Chinese Academy of Sciences, Beijing, 100190, China and [2]School of Mathematical Sciences, University of Chinese Academy of Sciences, Beijing, 100049, China

[*]huangjz@lsec.cc.ac.cn; lwan@amss.ac.cn

Abstract

Background: Dimensionality reduction and visualization play vital roles in single-cell RNA sequencing (scRNA-seq) data analysis. While they have been extensively studied, state-of-the-art dimensionality reduction algorithms are often unable to preserve the global structures underlying data. Elastic Embedding (EE), a nonlinear dimensionality reduction method, has shown promise in revealing low-dimensional intrinsic local and global data structure. However, the current implementation of the EE algorithm lacks scalability to large-scale scRNA-seq data.
Results: We present a distributed optimization implementation of the EE algorithm, termed distributed Elastic Embedding (D-EE). D-EE reveals the low-dimensional intrinsic structures of data with accuracy equal to that of Elastic Embedding, and it is scalable to large-scale scRNA-seq data. It leverages distributed storage and distributed computation, achieving memory efficiency and high-performance computing simultaneously. In addition, an extended version of D-EE, termed distributed optimization implementation of time series Elastic Embedding (D-TSEE), enables the user to visualize large-scale time series scRNA-seq data by incorporating experimental temporal information. Results with a large-scale scRNA-seq data indicate D-TSEE can uncover oscillatory gene expression patterns by employing experimentally temporal information.
Conclusions: D-EE is a distributed dimensionality reduction and visualization tool. Its distributed storage and distributed computation technique allow us to efficiently analyze large-scale single-cell data at the cost of constant time speedup. The source code for D-EE algorithm based on C and MPI tailored to a High Performance Computing cluster is available at https://github.com/ShaokunAn/D-EE.

Key words: dimensionality reduction; distributed storage; distributed computation; large-scale data; single-cell sequencing.

Background

The advent of single-cell sequencing provides high-dimensional profiles of cellular states at single-cell resolutions (e.g., single-cell RNA sequencing (scRNA-seq) of transcriptomes), offering the opportunity to unveil intrinsic biological processes and mechanisms. Dimensionality reduction and visualization methods have been extensively studied, as they play vital roles in revealing the intrinsic structures underlying scRNA-seq high-dimensional data [?]. Nonetheless, it is still challenging for these state-of-the-art methods of dimensionality reduction and visualization to preserve both local and global structures of data in low-dimensional space. For example, the celebrated t-distributed Stochastic Neighbor Embedding (t-SNE) algorithm [?] is widely used in the single-cell community [?]. It emphasizes the preservation of local structures, but it often distorts global structures [? ? ?]. As a solution, the Uniform Manifold Approximation and Projection (UMAP) algorithm [?] was developed, with the aim to preserve global structures, drawing increasing attention in single-cell data analysis community [?]. However, a recent study showed that UMAP does not improve upon t-SNE in this

Compiled on: September 29, 2020.
Draft manuscript prepared by the author.

regard when using the same initialization [?], making the validity of UMAP debatable.

In contrast, Elastic Embedding (EE), a nonlinear dimensionality reduction method, attempts to preserve both local and global structures underlying the data [?]. To achieve this goal, EE penalizes the placement of latent points in close proximity away from dissimilar data points in high-dimensional space, thus resolving the difficulty of global structure preservation (see [?], or Methods for details). EE has attracted increasing interest among statistical researchers [?]. It has also shown remarkable performance on visualizing the intrinsic structures of scRNA-seq data [? ? ?]. However, the current implementations of the EE algorithm are not scalable to sample size N (e.g., number of cells). Thus, it cannot be used for large-scale scRNA-seq datasets. For example, the storage of the attractive and the repulsive weight matrixes of the EE algorithm is $\mathcal{O}(N^2)$.

Therefore, we present a distributed optimization implementation of EE, termed D-EE. D-EE not only reveals the low-dimensional intrinsic structures of data with the same accuracy as EE, but also is scalable to large-scale scRNA-seq data. It leverages distributed storage and distributed computation, achieving memory efficiency and high-performance computing simultaneously (Figure 1). In addition, a distributed optimization implementation of the time series EE (TSEE) algorithm [?], termed D-TSEE, is also provided for visualizing large-scale time series scRNA-seq data. In this study, we demonstrate the power of D-EE and D-TSEE on both simulated and real data. Both D-EE and D-TSEE (1) achieve the same accuracy as EE and TSEE, respectively; (2) gain high strong scaling performance on large-scale dataset.

Methods

Elastic Embedding algorithm

EE was proposed by [?]. It optimizes an energy function containing the attractive and repulsive terms.

Given N samples $\mathbf{Y} = \{y_1, y_2, \ldots, y_N\}$, where $y_i \in \mathbb{R}^D$ represents its high-dimensional coordinates, the goal of EE is to map the data from high-dimensional space onto a low-dimensional representation $\mathbf{X} = \{x_1, x_2, \ldots, x_N\}$ with $x_i \in \mathbb{R}^d$ and $d \ll D$ by minimizing an energy function

$$E(\mathbf{X}, \lambda) = \sum_{m,n=1}^{N} w^{+}_{nm}\|x_n - x_m\|^2 + \lambda \sum_{m,n=1}^{N} w^{-}_{nm}\exp(-\|x_n - x_m\|^2),$$

where $w^{+}_{nm} = \exp(-\frac{1}{2}\|y_n - y_m\|^2/\sigma_n^2)$ and $w^{-}_{nm} = \|y_n - y_m\|^2$. The first term acts as an *attractive* force to preserve local distances, while the second term acts as a *repulsive* force to preserve global structures or to separate latent points. The parameter $\lambda \in \mathbb{R}^+$ trades off the two terms, and a larger value implies preservation of global structures is more important. In single-cell data analysis, with $\lambda = 10$, EE can achieve robust performance with high accuracy [?]. Therefore, we set the default value of λ as 10 for D-EE.

The σ_m in w^{+}_{mn} is a sample-specific scaling parameter. It is estimated adaptively by solving a sample-specific root-finding problem, such that the sample-specific distribution over its neighbors has a desired perplexity (see [?] for details). We set a default value of perplexity as 20 in this study. It is worth to note that, a newly proposed combinational perplexity has been applied to t-SNE [?], which greatly enhances the performance of t-SNE in preservation of global structures. The combinational perplexity can be also adopted by D-EE in future update.

An extension of EE, TSEE [?], was recently proposed to handle the dimensionality reduction problems of time series scRNA-seq data. It works by minimizing

$$E(\mathbf{X}, \lambda) = \sum_{m,n=1}^{N} w^{+}_{nm}\|x_n - x_m\|^2 + \lambda \sum_{m,n=1}^{N} (w^{-}_{nm} + \beta t_{nm})\exp(-\|x_n - x_m\|^2),$$

where t_{nm} represents the dissimilarity of time of pairwise points, and β trades off the weights between dissimilarities of time stages and expression space.

Numerical optimization of EE

Since the optimization solution of TSEE is basically the same as that of EE, we only give the numerical solution of EE. First, we denote $\mathbf{W_P} = \{w^{+}_{nm}\}$ and $\mathbf{W_N} = \{w^{-}_{nm}\}$. Owing to the existence of parameters $\{\sigma_n\}$, $\mathbf{W_P}$ is not a symmetrical matrix but we make it to be symmetric by taking $\mathbf{W_P} := \mathbf{W_P} + \mathbf{W_P}^T$. Next, the diagonal elements of $\mathbf{W_P}$ and $\mathbf{W_N}$ are set to zero. Finally, each element is normalized by dividing the sum of all elements in the matrix.

To solve the optimization problem, the classic Quasi-Newton methods update $\mathbf{X_{k+1}}$ according to $\mathbf{X_{k+1}} = \mathbf{X_k} + \alpha_\mathbf{k}\mathbf{P_k}$ in the k-th iteration, where $\alpha_\mathbf{k}$ is the step length determined by a line search procedure, and $\mathbf{P_k}$ is the search direction obtained by solving a Jacobian system $\mathbf{B_k}\mathbf{P_k} = -\mathbf{G_k}$. In this equation, $\mathbf{B_k}$ is positive-definite to guarantee the decrease of objective function. $\mathbf{G_k} = \mathbf{L_k}\mathbf{X_k}$ is the gradient of the objective function in the k-*th* iteration, where $\mathbf{L_k}$ is the Laplacian of $\mathbf{W_k} = \{w^{(k)}_{mn}\}$ with $w^{(k)}_{mn} = w^{+}_{mn} - \lambda w^{-}_{mn}\exp(-\|x^{(k)}_n - x^{(k)}_m\|^2)$. These procedures are repeated until a certain termination criterion is satisfied. During the iteration, $\mathbf{B_k}$ generally needs to be updated in each iteration as well.

When optimizing the EE-like optimization problems, a technique termed Partial-Hessian optimization strategies has been proposed to employ partial information of Hessian $\mathbf{L_P}$ [?], which is the Laplacian of $\mathbf{W_P}$ and is invariant in each iteration. This invariance makes it possible to utilize some precondition approaches, e.g., LU decomposition, to improve calculation efficiency. The effectiveness of the determined direction, called Spectral Direction, has been validated experimentally in previous work [?].

D-EE algorithm

We provide a distributed optimization implementation of EE, termed D-EE. The overview of the newly proposed D-EE algorithm is given in Figure 1. During whole optimization implementation, multiple processes are employed for computation and storage of data. In Figure 1, two processes, $\mathcal{P}_0$ and $\mathcal{P}_1$, are taken as an example. To achieve high performance in computing and memory efficiency simultaneously, our proposed distributed algorithm divides data ($\mathbf{W_P}$, $\mathbf{W_N}$, and $\mathbf{G_k}$) by rows for the multiple processes assigned. To avoid frequent communication, the whole original high-dimensional data $\mathbf{Y}$ is read and stored in each process, and the low-dimensional embedding $\mathbf{X}$ is established in each process as well since the storage consumed by $\mathbf{Y}$ and $\mathbf{X}$ is much less when compared to other $N \times N$ matrixes used during computation. It is worth to note that, since most of the computation of each row in one matrix generally merely depends on the same row of other matrixes (see the approximated computational complexity of D-EE in the following section for details), the partition procedure we design in the

D-EE algorithm is an almost optimal partition in parallel computing as a result of the optimal leverage of computation and communication. On the one hand, the total computational cost of the D-EE algorithm is almost the same as that of the centralized algorithm of EE. On the other hand, most procedures in the D-EE algorithm are communication-free, as shown in Figure 1 by black arrow. Even though some procedures still exist with communication, as shown in Figure 1 by blue arrow, the communication volume is in a much lower order than the cost of computation.

Computation of matrixes $\mathbf{D}, \mathbf{W_P}, \mathbf{W_N}$

As mentioned before, the matrixes $\mathbf{W_P}, \mathbf{W_N}$ depend on the high-dimensional data $\mathbf{Y}$ and $\{\sigma_n\}_{n=1}^N$. Since each σ_n is obtained by solving a root-finding problem from the n-th row of the distance matrix $\mathbf{D}$, each matrix is equally, or almost equally, partitioned into multiple nonoverlapping parts by rows and stored in multiple processes, as shown in Figure 1A. Let us denote $\mathbf{D} = [\mathbf{D}^1, \cdots, \mathbf{D}^P]$, where sub-matrix $\mathbf{D}^i$ with size of $M_i \times N$ is stored in the i-th process, and P is the number of processes we used. The $[\cdots]$ represents a column vector. Similar notations are used for the other $N \times N$ matrixes. It is clear that each row of matrixes $\mathbf{D}, \mathbf{W_P}, \mathbf{W_N}$ depends on all original high-dimensional data $\mathbf{Y}$. Therefore, we load a copy of $\mathbf{Y}$ into each process to avoid frequent communication.

In the centralized implementation of EE, the parameters $\sigma_n, n = 1, \ldots, N$, are calculated by iteratively solving a sequence of root-finding problems. The iteration method for the root-finding problems is improved by reordering the computation of $\{\sigma_n\}_{n=1}^N$ according to the distances of all samples ($\mathbf{Y}$), which is also the complete distance matrix $\mathbf{D}$ [?]. Then the reordered root-finding problems are sequentially solved by taking the solution of the previous one as the initial value of the next. Since the parameters are distributed in different processes, it is clear that the sequential root-finding approach cannot be parallelized without modifications. In the D-EE algorithm, we calculate $\{\sigma_n\}_{n=1}^N$ in the following parallel way. First, we decompose $\{\sigma_n\}_{n=1}^N$ into P subsets as $\Sigma_i = \{\sigma_n\}_{n=\mathcal{M}_i+1}^{\mathcal{M}_{i+1}}$ with $i = 0, \cdots, P-1$. The elements in the i-th subset Σ_i are computed and stored in the i-th process. Similar to the centralized algorithm of EE, we then reorder Σ_i according to the distance matrix $\mathbf{D}^i$ and iteratively solve the corresponding root-finding problems within the i-th process. According to the distributions of the initial data $\mathbf{Y}$ and the matrixes established before, we conclude that the D-EE algorithm calculates $\{\sigma_n\}_{n=1}^N$ in parallel, which is communication-free. The efficiency of the root-finding approach is also guaranteed by the local order. Since we only change the order and initial guesses of the root-finding problems, the solutions of the root-finding problems, as obtained from D-EE, are almost the same as those from EE. With the whole original high-dimensional data $\mathbf{Y}$ and the subset Σ_i, we can compute the following submatrixes $\mathbf{W_P}^i$, $\mathbf{W_N}^i$ in the i-th process. Thus, we give a parallel and communication-free approach to compute matrixes $\mathbf{D}$, $\mathbf{W_P}$, $\mathbf{W_N}$.

Normalization of $\mathbf{W_P}$ and $\mathbf{W_N}$

After computing matrixes $\mathbf{W_P}$, $\mathbf{W_N}$, each process sets the diagonal elements belonging to it as 0 in parallel. Then, we set $\mathbf{W_P} := \mathbf{W_P} + \mathbf{W_P}^T$ such that $\mathbf{W_P}$ becomes a symmetric matrix. Let us denote $\mathbf{W_P}^T := \hat{\mathbf{W}}_\mathbf{P} = [\hat{\mathbf{W}}_\mathbf{P}^1, \cdots, \hat{\mathbf{W}}_\mathbf{P}^P]$, where submatrix $\hat{\mathbf{W}}_\mathbf{P}^i$ has the size of $M_i \times N$. In the i-th process, we first obtain the elements of the submatrix $\hat{\mathbf{W}}_\mathbf{P}^i$ from the other $P-1$ processes by communication and then compute $\mathbf{W_P}^i := \mathbf{W_P}^i + \hat{\mathbf{W}}_\mathbf{P}^i$. Here point-to-point communication happens, and the communication volume for each process is $\mathcal{O}(N^2/P)$.

To normalize the matrixes $\mathbf{W_P}$, $\mathbf{W_N}$, each element should be divided by the sum of all elements in the matrix. The sum of all elements in matrix $\mathbf{W_P}$ is parallel computed as follows. First, each process calculates the sum of all elements in the submatrix $\mathbf{W_P}^i$ independently. We denote the sum of all elements in the submatrix $\mathbf{W_P}$ and $\mathbf{W_P}^i$ as $\mathcal{S}$ and $\mathcal{S}^i$, respectively. Then we compute the sum of all elements in matrix $\mathbf{W_P}$ by $\mathcal{S} = \sum_{i=1}^P \mathcal{S}_i$ through an MPI_Allgather action. Here all-to-all communication happens, and the communication volume for each process is $\mathcal{O}(P)$. Then, we normalize matrix $\mathbf{W_P}$ in each process by taking $\mathbf{W_P}^i = \mathbf{W_P}^i/\mathcal{S}$ in parallel without communication. The normalization of matrix $\mathbf{W_N}$ is done in a similar way.

Computation of low-dimensional embedding X

After normalizing $\mathbf{W_P}$, its Laplacian $\mathbf{L_P}$, which is needed for the subsequent determination of descent direction, is computed in parallel as follows. In the i-th process, we calculate the elements of submatrix $\mathbf{L_P}^i$ by using $l_{mn}^+ = \sum_{k=1}^N w_{mk}^+ - w_{mn}^+$, where l_{mn}^+ and w_{mn}^+ are the elements of matrixes $\mathbf{L_P}$ and $\mathbf{W_P}$, respectively. Since the two matrixes are partitioned by row in the same way, the computation of $\mathbf{L_P}$ is also communication-free.

The low-dimensional embedding $\mathbf{X}$ is obtained by solving the optimization problem with the Partial-Hessian optimization strategy. During the Quasi-Newton procedures, a dense linear system $\mathbf{L_P}\mathbf{P_k} = -\mathbf{G_k}$ must be solved in parallel. In the D-EE algorithm, we perform LU decomposition on $\mathbf{L_P}$. Considering that $\mathbf{L_P}$ is positive semi-definite, but not positive definite, a small value μ is added to the diagonal of $\mathbf{L_P}$ in practice. During the following sections, we still use $\mathbf{L_P}$ to denote the adjusted matrix. LU decomposition on $\mathbf{L_P} = \mathcal{L}\mathcal{U}$ is done with PETSc, which provides uniform and efficient access to all linear system solvers in the package, including parallel and sequential, direct and iterative [? ? ?]. Here, $\mathcal{L}$ and $\mathcal{U}$ are the corresponding lower and upper triangle matrixes, respectively. With the decomposition of LU, the dense linear system $\mathbf{L_P}\mathbf{P_k} = -\mathbf{G_k}$ is replaced by two sublinear systems $\mathcal{L}\hat{\mathbf{P}}_\mathbf{k} = -\mathbf{G_k}$ and $\mathcal{U}\mathbf{P_k} = \hat{\mathbf{P}}_\mathbf{k}$, which can be solved by the backward substitution method.

As shown in Figure 1D, the partitions of $\mathbf{L_P}$, $\mathcal{L}$, and $\mathcal{U}$ are the same as $\mathbf{W_P}$. Let us denote $\mathbf{P_k} = [\mathbf{P_k}^1, \cdots, \mathbf{P_k}^P]$, where submatrix $\mathbf{P_k}^i$ with size of $M_i \times d$ is stored in the i-th process, and a similar partition is performed on $\mathbf{G_k}$. Based on the partitions of $\mathbf{L_P}$, $\mathcal{L}$, $\mathcal{U}$, $\mathbf{P_k}$, and $\mathbf{G_k}$, the computational complexities per process of LU decomposition and backward substitution are $\mathcal{O}(N^3/P)$ and $\mathcal{O}(N^2/P)$, with corresponding communication volumes of $\mathcal{O}(N^2/P)$ and $\mathcal{O}(N/P)$, respectively. According to the analysis, LU decomposition is only done in the first iteration of the Quasi-Newton method, and matrixes $\mathcal{L}$ and $\mathcal{U}$ are stored and reused during the whole Quasi-Newton procedure.

The gradient $\mathbf{G_k}$ in the right-hand side of the linear system $\mathbf{L_P}\mathbf{P_k} = -\mathbf{G_k}$ is calculated by $\mathbf{G_k} = \mathbf{L_k}\mathbf{X_k}$, where the $N \times N$ matrix $\mathbf{L_k}$ depends on matrixes $\mathbf{W_P}$, $\mathbf{W_N}$, and $\mathbf{Ker}$. Here the elements of matrix $\mathbf{Ker}$ are defined as $\ker_{mn} = \exp(-\|x_m - x_n\|^2)$, and the elements of matrix $\mathbf{L_k}$ are defined as $l_{mn}^{(k)} = w_{mn}^+ - \lambda w_{mn}^- \ker_{mn}^{(k)}$. As shown in Figure 1E, the partitions of matrixes $\mathbf{L_k}$ and $\mathbf{Ker}$ are the same as those of $\mathbf{W_P}$. In D-EE, we store all elements of $\mathbf{X_k}$ in each process, which is the same as the original high-dimensional data $\mathbf{Y}$. Thus, we can parallel compute matrixes $\mathbf{L_k}$ and $\mathbf{Ker}$ in the same way with the matrix $\mathbf{W_N}$, which means the procedure is also communication-free.

After solving the linear system, we obtain the search direction $\mathbf{P_k}$. Then, we update $\mathbf{X_{k+1}}$ according to $\mathbf{X_{k+1}} = \mathbf{X_k} + \alpha_k\mathbf{P_k}$, where α_k is determined by a line search approach. As men-

tioned before, the low-dimensional embedding $\mathbf{X_k}$ is stored sequentially, but $\mathbf{P_k}$ is distributed stored. Thus, we first compute the elements of submatrix $\mathbf{P_{k+1}}^i$ in the i-th process and then gather all elements of $\mathbf{P_k}$ in each process by the all-gather function in MPI. Here all-to-all communication happens, and the order of communication volume for each process is $\mathcal{O}(Nd)$. In line search steps, we need to calculate the energy function $E(\mathbf{X}, \lambda)$ several times, which is computed in parallel according to the following formula

$$E(\mathbf{X}, \lambda) = \sum_{i=1}^{P} \left(\sum_{m=\mathcal{M}_i+1}^{\mathcal{M}_{i+1}} \sum_{n=1}^{N} \left\{ w^{+}_{mn} \|x_n - x_m\|^2 + \lambda w^{-}_{mn} \exp(-\|x_n - x_m\|^2) \right\} \right).$$

The summation included in the parentheses is calculated in each process simultaneously and then gathered by the MPI all-gather function. Here all-to-all communication happens, and the communication volume for each process is $\mathcal{O}(P)$.

Results

Data Description

We test the accuracy and scalability of D-EE on three datasets. The first simulated dataset [?], named PHATE data for convenience, consists of 1,440 samples and 60 features. It is a complex tree structure which simulates a cellular developmental process, namely, progressions, branch or split in progressions and end state of progression, composed of ten branches in total. We first perform principal component analysis (PCA) on the original data, reserving a 1,440 samples $\times$ 7 features matrix.

The second dataset characterizes process of mouse hematopoietic stem and progenitor cells (HSPCs) bifurcating to myeloid and erythroid precursors [?], consisting of 4,423 samples. The obtained single-cell read count data is pre-processed by Seurat package (version 3.2.1) [? ?]. Firstly, we normalize the gene expression in each sample as follows: we divide each gene read count by the total read counts for each cell, and then multiply a scale factor of 10^4 and plus one, followed by taking a logarithmic transformation. Secondly, we select the top 2,000 variable genes using the default "vst" method of Seurat package, i.e., variance-stabilizing transformation [?]. Finally, we conduct PCA on the processed data, and select the top 50 largest principal components, resulting a 4,423 samples $\times$ 50 features matrix as input of EE and D-EE.

The third data is a large-scale time series scRNA-seq dataset containing ~250k cells [?]. The data characterizes reprogramming of fibroblasts to induced pluripotent stem cells (iPSCs), which was collected at half-day intervals across 18 days, resulting in 39 time points. Since the final time point of the iPSCs status was not annotated temporally, we therefore set the final point as 20-th day as the input to D-TSEE. We pre-process this data with Seurat package as well. Same as the pre-process of HSPCs data, we first filter cells and genes to include cells where at least 200 features are detected and to include genes detected in at least 50 cells, obtaining 259,081 cells and 19,427 genes. After that, we perform logarithmic transformation, select variable features and perform PCA as described in HSPCs dataset, obtaining a 259,081 samples $\times$ 50 features matrix as input of dimensionality reduction methods.

D-EE achieves high strong scaling efficiency

We evaluate D-EE using both simulated and real scRNA-seq datasets. The numerical tests are carried out on the LSSC-IV supercomputer. The 400 computing nodes of LSSC-IV are comprised of two 18-core Intel Xeon Gold CPUs with 192 GB local memory, and are interconnected via a proprietary high performance network. First, we employ PHATE data and HSPCs data to test the consistency between D-EE and EE results. We employ 36 processes in both D-EE algorithms. The low dimensions are set to 2 for the convenience of visualization for both datasets, and the parameter λ used is set to the default value 10. We use the same initialization generated from Gaussian distribution as that in the original EE Matlab code.

D-EE achieves consistent 2-dimensional embedding as that of EE (Figure 2). To measure the consistency quantitatively, we calculate the relative error which is defined by

$$\text{relative error} = \frac{\|A - B\|_F}{\|A\|_F},$$

where $\|\cdot\|_F$ is the Frobenius norm of matrix, A and B represent the output of EE and D-EE, respectively. Frobenius norm of a matrix $A \in \mathbb{R}^{m \times n}$ is defined as

$$\|A\|_F = \sqrt{\sum_{i=1}^{m} \sum_{j=1}^{n} |a_{ij}|^2}.$$

Their relative errors of D-EE in HSPCs dataset and PHATE dataset are 2.42×10^{-6} and 1.60×10^{-6}, respectively, thus further validating the consistency of results by D-EE and EE.

To test performance of parallel efficiency of D-EE on the large-scale dataset, we apply D-EE to the iPSCs dataset (~ 250k cells) using 500, 1,000, 2,000, and 4,000 processes, respectively, and for each setting of number of processes we run at least twice. The averaged computation times of D-EE for iPSCs dataset are 5.83 hours, 3.19 hours, 2.36 hours, and 2.02 hours, when the numbers of processes are 500, 1,000, 2,000, and 4,000, respectively. Next, we evaluate the parallel performance of D-EE based on two widely used indexes, the strong scaling speedup ratio and the parallel efficiency. The strong speedup ratio is defined as

$$S = \frac{T_s}{T_p},$$

where T_s and T_p are the time of computation by using single process and p processes, respectively. The parallel efficiency is defined as

$$E = \frac{S}{p} = \frac{T_s}{pT_p}.$$

The ideal strong speedup should be p, and the corresponding parallel efficiency should be 1 when p processes are used. However, it is impossible to run the data with 250k samples on a single process owing to limited memory and low efficiency of EE algorithm. Thus, we take the time of computation of 500 processes as T_s with $s = 500$, and the parallel efficiency is then reformulated as

$$E = \frac{sT_s}{pT_p}.$$

When adopting the speedup ratio and parallel efficiency as the indexes for scaling, a strong scaling performance at remarkable speedup is observed when increasing CPUs from 500 to 4,000 processes (Figure 3A, B).

We further evaluate the performance of D-EE on computational times in our supercomputer. Three test cases with sample sizes being 10k, 50k, and 100k are run on 500, 1,000, and 2,000 processors, respectively. It is shown that when using the same number of processes, it is naturally that the computational time increases as total sample size increases (as shown in Figure 3C).

In practice, for analysis of large-scale single-cell dataset, a workstation with multiple CPUs and large memory is recommended. Meanwhile, we also test D-EE on datasets at different sample sizes, and find that D-EE efficiently implements on a typical personal computer (e.g., 8 CPU threads, 16 Gb RAM) with up to a sample size of 12k, while on a conventional workstation (e.g., 40 CPU threads, 256 Gb RAM) with up to a sample size of 48k, respectively.

D-EE and D-TSEE recover intrinsic low-dimensional structures of large-scale scRNA-seq data

We illustrate the application of D-EE and D-TSEE on the large-scale iPSCs dataset. We visualize the iPSCs data on the 2-dimensional space using t-SNE, UMAP, D-EE and D-TSEE, respectively (Figure 4). The same pre-processed single-cell data is used for all four methods. The t-SNE is conducted by the FIt-SNE method [?] with a PCA initialization, and we choose the learning rate as 1/12 of the sample size, according to [?] for better preservation of the global structures. UMAP is conducted by adjusting the number of nearest neighbors (NNs) to balance the preservation of local and global structures. We find that UMAP is not sensitive when choosing the number of NNs from 30 (defaults) to 500 (square root of the number of samples), and we thus set the number of NNs to be 100 in our study. Both t-SNE and UMAP are implemented by the Seurat software (version 3.2.1). In our implementations, both D-EE and D-TSEE are used with their default parameters.

We color the cells based on time stages on their low-dimensional embeddings obtained by the four dimensionality reduction results (Figure 4). We find that t-SNE preserves the time lineage structure of data in an "S" shape with small gaps (Figure 4, upper-left panel); UMAP also shows a time lineage structure, but with a large gap occurring between time stages 5.5 and 6 (Figure 4, upper-right panel). In contrast, both D-EE and D-TSEE preserve continuous time lineage structures in low-dimensional space (Figure 4, lower panels).

We further explore the gene expression patterns of Sox2, Sox4 and Nanog on both D-EE and D-TSEE embeddings (Figure 5). These genes are key regulators during stem cell differentiation and reprogramming process [? ? ?]. Previous study has shown evidence that these genes may oscillate during cell development progression [? ?]. These genes display oscillatory gene expression patterns in the early stage of iPSCs on the D-TSEE view (Figure 5), providing useful information and clues for downstream analysis.

Conclusion

In this work, we develop a novel tool, D-EE, for visualizing large-scale scRNA-seq data. D-EE implements the distributed storage and distributed computing techniques to a powerful nonlinear dimensionality reduction method, Elastic Embedding. The optimal distributed computational strategies implemented by D-EE allow it to achieve not only the strong scalability on large-scale dataset, but also the exact optimization solution as original EE by fully utilizing the whole data. Numerical experiments validate the correctness and parallel efficiency of D-EE. Considering the emergence of time series scRNA-seq data, our D-TSEE tool allows us efficiently to perform dimensionality reduction on large-scale single-cell data by employing experimentally temporal information. Besides, when incorporating temporal information if it is available, D-TSEE can reveal dynamic gene expression patterns, providing insights for subsequent analysis of molecular mechanisms and dynamic transition progression.

We demonstrate that D-EE and D-TSEE work efficiently on large-scale datasets at a super computer. However, the proposed distributed algorithm D-EE still has disadvantage due to the huge computational cost and storage with a relative large number of cells. Therefore, D-EE is limited to handle and analyze huge-scale datasets with the number of cells up to the order of millions [?]. In comparison, the state-of-the-art accelerated implementations of t-SNE (e.g., FIt-SNE) and UMAP are of the close-to-linear computational complexities, showing great efficiency on huge data analysis.

In the future study, to resolve the limitation of D-EE on huge-scale data computation, we can accelerate D-EE by adopting either the fast Fourier transform as used in FIt-SNE, or adopting the state-of-the-art neural network framework used by net-SNE [?]. On the other hand, since huge-scale single-cell dataset can be highly redundant, we can also select subset of informative samples using the advanced geometric sketching tool [?] prior to application of D-EE.

Availability of source code and requirements

Lists the following:

- Project name: D-EE
- Project home page: https://github.com/ShaokunAn/D-EE
- Operating system(s): Linux
- Programming language: C, R
- Other requirements: Multi-core processor, implementation of MPI library (i.e., OpenMPI or IntelMPI) installed on each node of the cluster, a reasonably fast interconnecting infrastructure, PETSc 3.11.4 or higher
- License: GNU General Public License
- biotools: d-ee

Availability of supporting data and materials

The PHATE data supporting the results of this article is available in the Github repository [?]. The iPSCs data is available in NCBI repository with number GSE 122662 [?]. The HPSCs data is available in the NCBI repository with accession number GSE72857 and the dataset used in our study is downloaded from their Github https://github.com/ManuSetty/wishbone.

Declarations

List of abbreviations

D-EE: distributed optimization implementation of Elastic Embedding; D-TSEE: distributed optimization implementation of time series Elastic Embedding; EE: Elastic Embedding; scRNA-seq: single-cell RNA sequencing; PCA: Principal Component Analysis; TSEE: time series Elastic Embedding; t-SNE: t-distributed Stochastic Neighbor Embedding; UMAP: Uniform Manifold Approximation and Projection; NNs: nearest neighbors

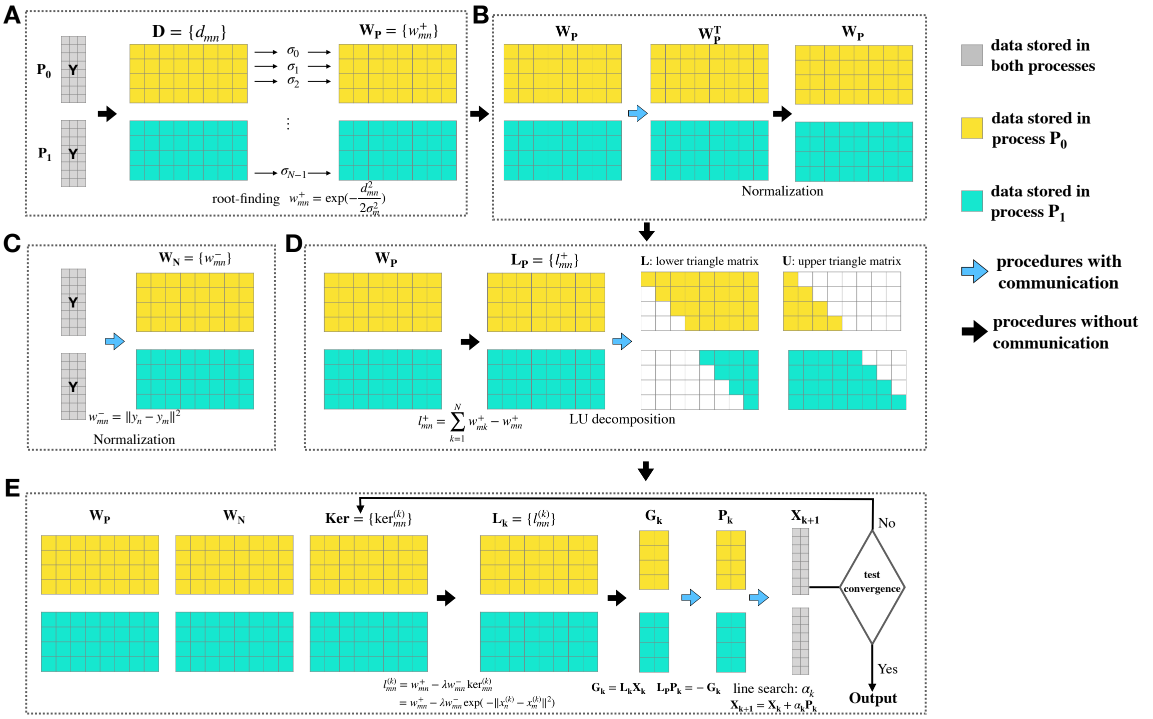

Figure 1. Overview of D-EE algorithm. The D-EE algorithm can be decomposed into five parts. Part A: the computation of matrixes **D** and $\mathbf{W_P}$. To obtain $\mathbf{W_P}$, the parameters $\{\sigma_n\}$ are determined by solving a series of root-finding problems. Part B: the symmetry and normalization of the matrix $\mathbf{W_P}$. Part C: the computation and normalization of $\mathbf{W_N}$. Part D: the computation of the Laplacian matrix $\mathbf{L_P}$ together with LU decomposition. Part E: the computation of $\mathbf{X_{k+1}}$ by solving an optimization problem with the classic Quasi-Newton methods iteratively. In the *k-th* iteration, $\mathbf{L_k}$ is computed based on $\mathbf{W_P}$, $\mathbf{W_N}$ and $\mathbf{X_k}$, which are used to obtain the gradient $\mathbf{G_k} = \mathbf{L_k X_k}$. The descent direction $\mathbf{P_k}$ is then determined by solving a linear system $\mathbf{L_P P_k} = -\mathbf{G_k}$. Finally, $\mathbf{X_{k+1}}$ is updated according to $\mathbf{X_{k+1}} = \mathbf{X_k} + \alpha_k \mathbf{P_k}$, where the step size α_k is calculated by a line search method.

Consent for publication

Not applicable.

Competing Interests

The authors declare that they have no competing interests.

Funding

This work is supported by the National Key R&D Program of China under Grant 2018YFB0704304, NSFC grants (Nos. 11871069, 12071466), NCMIS of CAS, LSEC of CAS, LSC of CAS, and the Youth Innovation Promotion Association of CAS.

Author's Contributions

Conceptualization and Methodology: S.A., L.W. Software: S.A., J.H. Supervision: S.A., L.W., J.H. Funding Acquisition: L.W., J.H. Writing – Original Draft Preparation: S.A. Writing – Review & Editing: all authors.

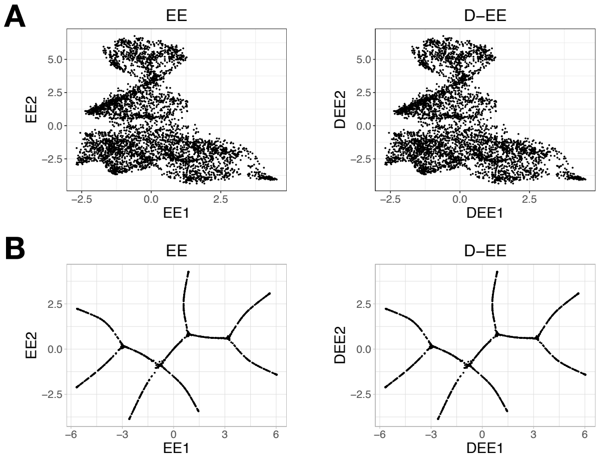

Figure 2. D-EE and EE achieve the same results on two datasets when using the same initializations. A: The 2-D mapping of HSPCs data obtained by the two algorithms. B: The 2-D mapping of PHATE data obtained by the two algorithms.

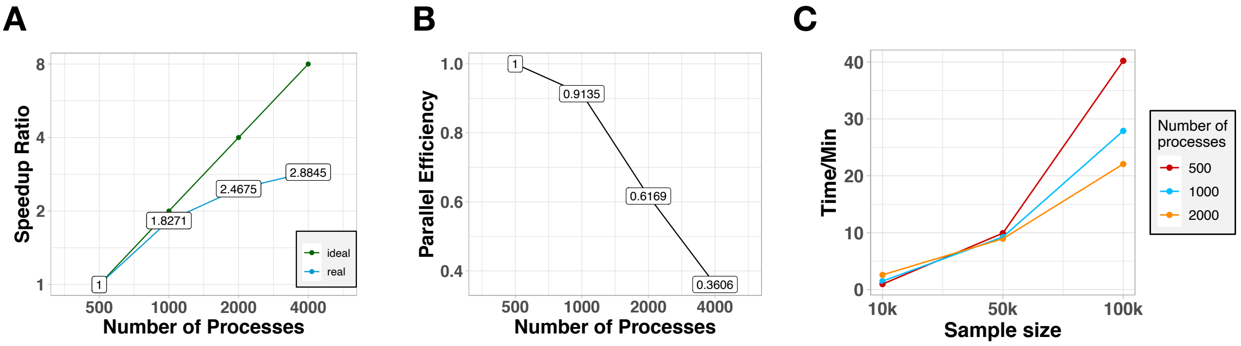

Figure 3. Strong scaling results and parallel efficiency of the D-EE algorithm on an LSSC-IV supercomputer. We apply D-EE on iPSCs dataset by using 500, 1,000, 2,000, and 4,000 processes, respectively. A: The strong speedup ratio increases with increase in the number of processes.The green line represents the ideal speedup ratio and the blue line represents the speedup ratio obtained by D-EE. B: The parallel efficiency decreases at an acceptable rate with the increase in number of processes. C: Computational times consumed for 10k, 50k, 100k samples under 500, 1000 and 2000 processes, respectively.

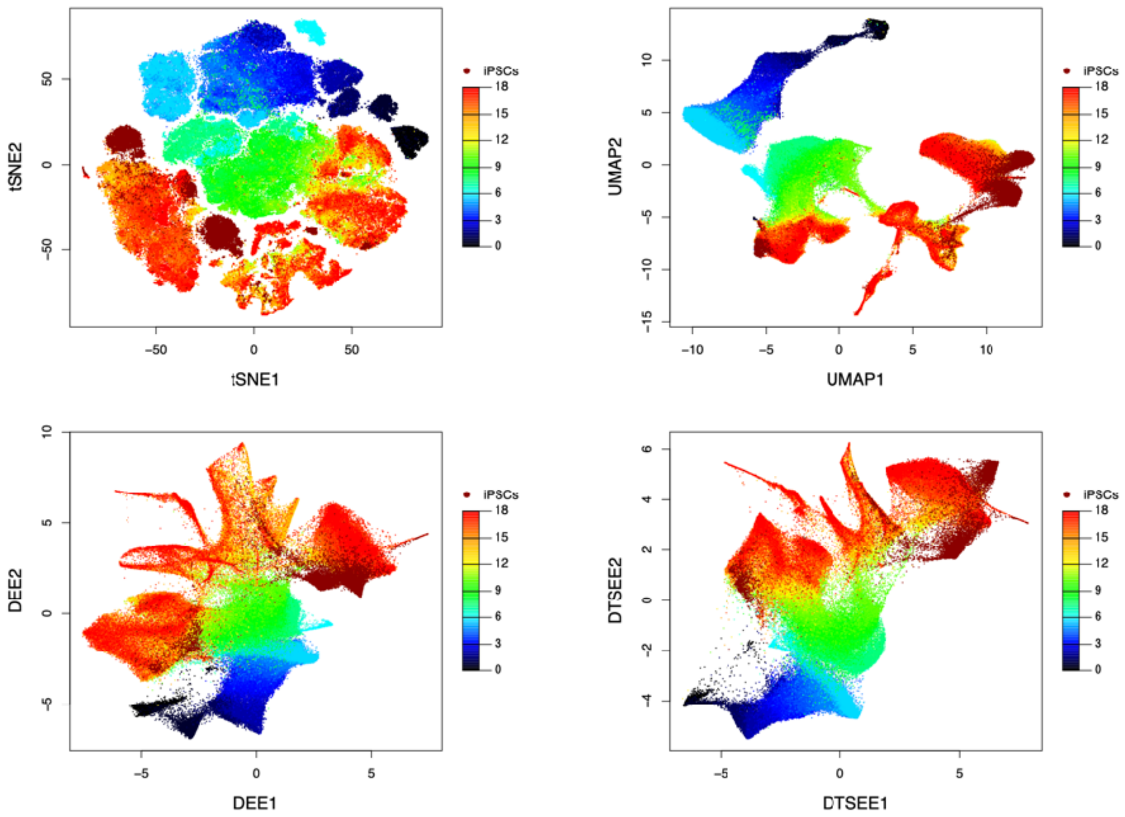

Figure 4. Cells are colored by time stages in iPSCs dataset on the 2-dimensional space obtained by four dimensionality reduction methods, i.e., t-SNE, UMAP, D-EE and D-TSEE.

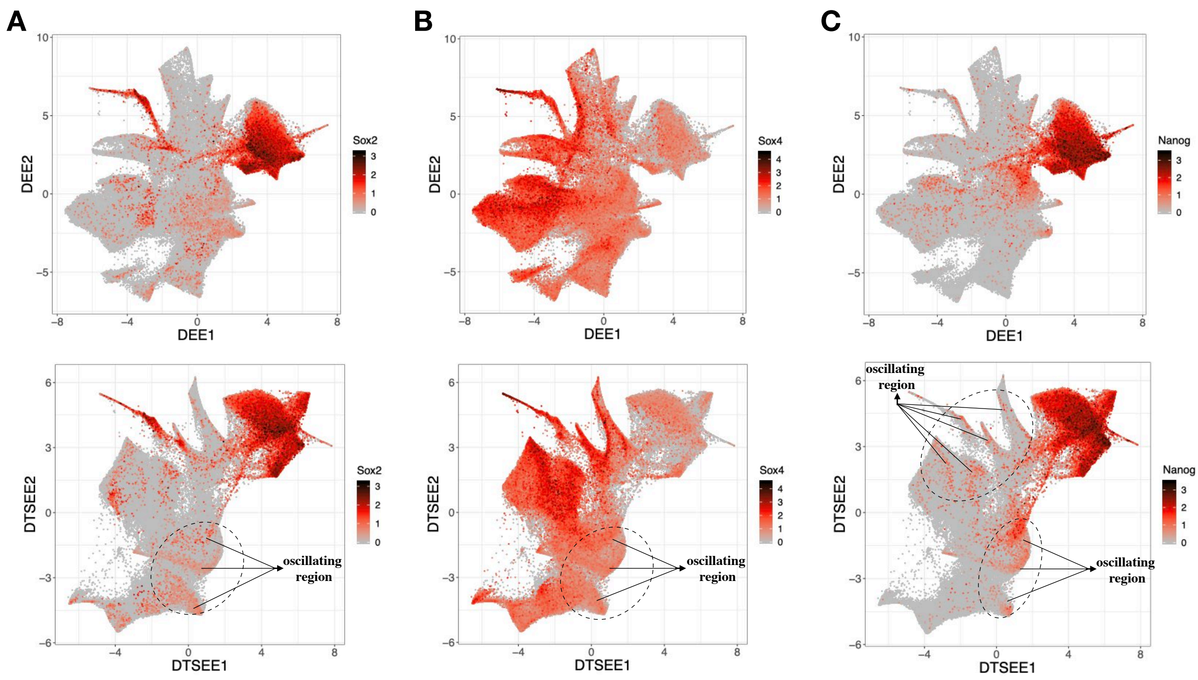

Figure 5. Cells are colored by gene expression of Sox2, Sox4, and Nanog in iPSCs dataset on the 2-dimensional embeddings obtained by D-EE and D-TSEE, respectively.

Manuscript
Click here to access/download;Manuscript;main.pdf

GigaScience, 2020, 1–9

doi: xx.xxxx/xxxx
Manuscript in Preparation
Technical Note

TECHNICAL NOTE

D-EE: a distributed software for visualizing intrinsic structure of large-scale single-cell data

Shaokun An[1,2], Jizu Huang[1,2,*] and Lin Wan[1,2,*]

[1]NCMIS, LSEC, LSC, Academy of Mathematics and Systems Science, Chinese Academy of Sciences, Beijing, 100190, China and [2]School of Mathematical Sciences, University of Chinese Academy of Sciences, Beijing, 100049, China

[*]huangjz@lsec.cc.ac.cn; lwan@amss.ac.cn

Abstract

Background: Dimensionality reduction and visualization play vital roles in single-cell RNA sequencing (scRNA-seq) data analysis. While they have been extensively studied, state-of-the-art dimensionality reduction algorithms are often unable to preserve the global structures underlying data. Elastic Embedding (EE), a nonlinear dimensionality reduction method, has shown promise in revealing low-dimensional intrinsic local and global data structure. However, the current implementation of the EE algorithm lacks scalability to large-scale scRNA-seq data.
Results: We present a distributed optimization implementation of the EE algorithm, termed distributed Elastic Embedding (D-EE). D-EE reveals the low-dimensional intrinsic structures of data with accuracy equal to that of Elastic Embedding, and it is scalable to large-scale scRNA-seq data. It leverages distributed storage and distributed computation, achieving memory efficiency and high-performance computing simultaneously. In addition, an extended version of D-EE, termed distributed optimization implementation of time series Elastic Embedding (D-TSEE), enables the user to visualize large-scale time series scRNA-seq data by incorporating experimental temporal information. Results with a large-scale scRNA-seq data indicate D-TSEE can uncover oscillatory gene expression patterns by employing experimentally temporal information.
Conclusions: D-EE is a distributed dimensionality reduction and visualization tool. Its distributed storage and distributed computation technique allow us to efficiently analyze large-scale single-cell data at the cost of constant time speedup. The source code for D-EE algorithm based on C and MPI tailored to a High Performance Computing cluster is available at https://github.com/ShaokunAn/D-EE.

Key words: dimensionality reduction; distributed storage; distributed computation; large-scale data; single-cell sequencing.

Background

The advent of single-cell sequencing provides high-dimensional profiles of cellular states at single-cell resolutions (e.g., single-cell RNA sequencing (scRNA-seq) of transcriptomes), offering the opportunity to unveil intrinsic biological processes and mechanisms. Dimensionality reduction and visualization methods have been extensively studied, as they play vital roles in revealing the intrinsic structures underlying scRNA-seq high-dimensional data [1]. Nonetheless, it is still challenging for these state-of-the-art methods of dimensionality reduction and visualization to preserve both local and global structures of data in low-dimensional space. For example, the celebrated t-distributed Stochastic Neighbor Embedding (t-SNE) algorithm [2] is widely used in the single-cell community [3]. It emphasizes the preservation of local structures, but it often distorts global structures [4, 5, 6]. As a solution, the Uniform Manifold Approximation and Projection (UMAP) algorithm [7] was developed, with the aim to preserve global structures, drawing increasing attention in single-cell data analysis community [8]. However, a recent study showed that UMAP does not improve upon t-SNE in this regard when

Compiled on: September 29, 2020.
Draft manuscript prepared by the author.

using the same initialization [9], making the validity of UMAP debatable.

In contrast, Elastic Embedding (EE), a nonlinear dimensionality reduction method, attempts to preserve both local and global structures underlying the data [4]. To achieve this goal, EE penalizes the placement of latent points in close proximity away from dissimilar data points in high-dimensional space, thus resolving the difficulty of global structure preservation (see [4], or Methods for details). EE has attracted increasing interest among statistical researchers [10]. It has also shown remarkable performance on visualizing the intrinsic structures of scRNA-seq data [1, 5, 11]. However, the current implementations of the EE algorithm are not scalable to sample size N (e.g., number of cells). Thus, it cannot be used for large-scale scRNA-seq datasets. For example, the storage of the attractive and the repulsive weight matrixes of the EE algorithm is $\mathcal{O}(N^2)$.

Therefore, we present a distributed optimization implementation of EE, termed D-EE. D-EE not only reveals the low-dimensional intrinsic structures of data with the same accuracy as EE, but also is scalable to large-scale scRNA-seq data. It leverages distributed storage and distributed computation, achieving memory efficiency and high-performance computing simultaneously (Figure 1). In addition, a distributed optimization implementation of the time series EE (TSEE) algorithm [11], termed D-TSEE, is also provided for visualizing large-scale time series scRNA-seq data. In this study, we demonstrate the power of D-EE and D-TSEE on both simulated and real data. Both D-EE and D-TSEE (1) achieve the same accuracy as EE and TSEE, respectively; (2) gain high strong scaling performance on large-scale dataset.

Methods

Elastic Embedding algorithm

EE was proposed by [4]. It optimizes an energy function containing the attractive and repulsive terms.

Given N samples $\mathbf{Y} = \{y_1, y_2, \ldots, y_N\}$, where $y_i \in \mathbb{R}^D$ represents its high-dimensional coordinates, the goal of EE is to map the data from high-dimensional space onto a low-dimensional representation $\mathbf{X} = \{x_1, x_2, \ldots, x_N\}$ with $x_i \in \mathbb{R}^d$ and $d \ll D$ by minimizing an energy function

$$E(\mathbf{X}, \lambda) = \sum_{m,n=1}^{N} w^{+}_{nm}\|x_n - x_m\|^2 + \lambda \sum_{m,n=1}^{N} w^{-}_{nm}\exp(-\|x_n - x_m\|^2),$$

where $w^{+}_{nm} = \exp(-\frac{1}{2}\|y_n - y_m\|^2/\sigma_n^2)$ and $w^{-}_{nm} = \|y_n - y_m\|^2$. The first term acts as an *attractive* force to preserve local distances, while the second term acts as a *repulsive* force to preserve global structures or to separate latent points. The parameter $\lambda \in \mathbb{R}^{+}$ trades off the two terms, and a larger value implies preservation of global structures is more important. In single-cell data analysis, with $\lambda = 10$, EE can achieve robust performance with high accuracy [5]. Therefore, we set the default value of λ as 10 for D-EE.

The σ_m in w^{+}_{mn} is a sample-specific scaling parameter. It is estimated adaptively by solving a sample-specific root-finding problem, such that the sample-specific distribution over its neighbors has a desired perplexity (see [12] for details). We set a default value of perplexity as 20 in this study. It is worth to note that, a newly proposed combinational perplexity has been applied to t-SNE [3], which greatly enhances the performance of t-SNE in preservation of global structures. The combinational perplexity can be also adopted by D-EE in future update.

An extension of EE, TSEE [11], was recently proposed to handle the dimensionality reduction problems of time series scRNA-seq data. It works by minimizing

$$E(\mathbf{X}, \lambda) = \sum_{m,n=1}^{N} w^{+}_{nm}\|x_n - x_m\|^2 + \lambda \sum_{m,n=1}^{N} (w^{-}_{nm} + \beta t_{nm})\exp(-\|x_n - x_m\|^2),$$

where t_{nm} represents the dissimilarity of time of pairwise points, and β trades off the weights between dissimilarities of time stages and expression space.

Numerical optimization of EE

Since the optimization solution of TSEE is basically the same as that of EE, we only give the numerical solution of EE. First, we denote $\mathbf{W_P} = \{w^{+}_{nm}\}$ and $\mathbf{W_N} = \{w^{-}_{nm}\}$. Owing to the existence of parameters $\{\sigma_n\}$, $\mathbf{W_P}$ is not a symmetrical matrix but we make it to be symmetric by taking $\mathbf{W_P} := \mathbf{W_P} + \mathbf{W_P}^T$. Next, the diagonal elements of $\mathbf{W_P}$ and $\mathbf{W_N}$ are set to zero. Finally, each element is normalized by dividing the sum of all elements in the matrix.

To solve the optimization problem, the classic Quasi-Newton methods update $\mathbf{X_{k+1}}$ according to $\mathbf{X_{k+1}} = \mathbf{X_k} + \alpha_\mathbf{k}\mathbf{P_k}$ in the k-th iteration, where $\alpha_\mathbf{k}$ is the step length determined by a line search procedure, and $\mathbf{P_k}$ is the search direction obtained by solving a Jacobian system $\mathbf{B_k}\mathbf{P_k} = -\mathbf{G_k}$. In this equation, $\mathbf{B_k}$ is positive-definite to guarantee the decrease of objective function. $\mathbf{G_k} = \mathbf{L_k}\mathbf{X_k}$ is the gradient of the objective function in the k-*th* iteration, where $\mathbf{L_k}$ is the Laplacian of $\mathbf{W_k} = \{w^{(k)}_{mn}\}$ with $w^{(k)}_{mn} = w^{+}_{mn} - \lambda w^{-}_{mn}\exp(-\|x^{(k)}_n - x^{(k)}_m\|^2)$. These procedures are repeated until a certain termination criterion is satisfied. During the iteration, $\mathbf{B_k}$ generally needs to be updated in each iteration as well.

When optimizing the EE-like optimization problems, a technique termed Partial-Hessian optimization strategies has been proposed to employ partial information of Hessian $\mathbf{L_P}$ [13], which is the Laplacian of $\mathbf{W_P}$ and is invariant in each iteration. This invariance makes it possible to utilize some precondition approaches, e.g., LU decomposition, to improve calculation efficiency. The effectiveness of the determined direction, called Spectral Direction, has been validated experimentally in previous work [13].

D-EE algorithm

We provide a distributed optimization implementation of EE, termed D-EE. The overview of the newly proposed D-EE algorithm is given in Figure 1. During whole optimization implementation, multiple processes are employed for computation and storage of data. In Figure 1, two processes, $\mathcal{P}_0$ and $\mathcal{P}_1$, are taken as an example. To achieve high performance in computing and memory efficiency simultaneously, our proposed distributed algorithm divides data ($\mathbf{W_P}$, $\mathbf{W_N}$, and $\mathbf{G_k}$) by rows for the multiple processes assigned. To avoid frequent communication, the whole original high-dimensional data $\mathbf{Y}$ is read and stored in each process, and the low-dimensional embedding $\mathbf{X}$ is established in each process as well since the storage consumed by $\mathbf{Y}$ and $\mathbf{X}$ is much less when compared to other $N \times N$ matrixes used during computation. It is worth to note that, since most of the computation of each row in one matrix generally merely depends on the same row of other matrixes (see the approximated computational complexity of D-EE in the following section for details), the partition procedure we design in the D-EE algorithm is an almost optimal partition in parallel com-

puting as a result of the optimal leverage of computation and communication. On the one hand, the total computational cost of the D-EE algorithm is almost the same as that of the centralized algorithm of EE. On the other hand, most procedures in the D-EE algorithm are communication-free, as shown in Figure 1 by black arrow. Even though some procedures still exist with communication, as shown in Figure 1 by blue arrow, the communication volume is in a much lower order than the cost of computation.

Computation of matrixes $\mathbf{D}, \mathbf{W_P}, \mathbf{W_N}$

As mentioned before, the matrixes $\mathbf{W_P}, \mathbf{W_N}$ depend on the high-dimensional data $\mathbf{Y}$ and $\{\sigma_n\}_{n=1}^N$. Since each σ_n is obtained by solving a root-finding problem from the n-th row of the distance matrix $\mathbf{D}$, each matrix is equally, or almost equally, partitioned into multiple nonoverlapping parts by rows and stored in multiple processes, as shown in Figure 1A. Let us denote $\mathbf{D} = [\mathbf{D}^1, \cdots, \mathbf{D}^P]$, where sub-matrix $\mathbf{D}^i$ with size of $M_i \times N$ is stored in the i-th process, and P is the number of processes we used. The $[\cdots]$ represents a column vector. Similar notations are used for the other $N \times N$ matrixes. It is clear that each row of matrixes $\mathbf{D}, \mathbf{W_P}, \mathbf{W_N}$ depends on all original high-dimensional data $\mathbf{Y}$. Therefore, we load a copy of $\mathbf{Y}$ into each process to avoid frequent communication.

In the centralized implementation of EE, the parameters $\sigma_n, n = 1, \ldots, N$, are calculated by iteratively solving a sequence of root-finding problems. The iteration method for the root-finding problems is improved by reordering the computation of $\{\sigma_n\}_{n=1}^N$ according to the distances of all samples ($\mathbf{Y}$), which is also the complete distance matrix $\mathbf{D}$ [12]. Then the reordered root-finding problems are sequentially solved by taking the solution of the previous one as the initial value of the next. Since the parameters are distributed in different processes, it is clear that the sequential root-finding approach cannot be parallelized without modifications. In the D-EE algorithm, we calculate $\{\sigma_n\}_{n=1}^N$ in the following parallel way. First, we decompose $\{\sigma_n\}_{n=1}^N$ into P subsets as $\Sigma_i = \{\sigma_n\}_{n=\mathcal{M}_i+1}^{\mathcal{M}_{i+1}}$ with $i = 0, \cdots, P-1$. The elements in the i-th subset Σ_i are computed and stored in the i-th process. Similar to the centralized algorithm of EE, we then reorder Σ_i according to the distance matrix $\mathbf{D}^i$ and iteratively solve the corresponding root-finding problems within the i-th process. According to the distributions of the initial data $\mathbf{Y}$ and the matrixes established before, we conclude that the D-EE algorithm calculates $\{\sigma_n\}_{n=1}^N$ in parallel, which is communication-free. The efficiency of the root-finding approach is also guaranteed by the local order. Since we only change the order and initial guesses of the root-finding problems, the solutions of the root-finding problems, as obtained from D-EE, are almost the same as those from EE. With the whole original high-dimensional data $\mathbf{Y}$ and the subset Σ_i, we can compute the following submatrixes ${\mathbf{W_P}}^i$, ${\mathbf{W_N}}^i$ in the i-th process. Thus, we give a parallel and communication-free approach to compute matrixes $\mathbf{D}$, $\mathbf{W_P}$, $\mathbf{W_N}$.

Normalization of $\mathbf{W_P}$ and $\mathbf{W_N}$

After computing matrixes $\mathbf{W_P}$, $\mathbf{W_N}$, each process sets the diagonal elements belonging to it as 0 in parallel. Then, we set $\mathbf{W_P} := \mathbf{W_P} + {\mathbf{W_P}}^T$ such that $\mathbf{W_P}$ becomes a symmetric matrix. Let us denote ${\mathbf{W_P}}^T := \hat{\mathbf{W}}_\mathbf{P} = [\hat{\mathbf{W}}_\mathbf{P}^1, \cdots, \hat{\mathbf{W}}_\mathbf{P}^P]$, where submatrix $\hat{\mathbf{W}}_\mathbf{P}^i$ has the size of $M_i \times N$. In the i-th process, we first obtain the elements of the submatrix $\hat{\mathbf{W}}_\mathbf{P}^i$ from the other $P-1$ processes by communication and then compute ${\mathbf{W_P}}^i := {\mathbf{W_P}}^i + \hat{\mathbf{W}}_\mathbf{P}^i$. Here point-to-point communication happens, and the communication volume for each process is $\mathcal{O}(N^2/P)$.

To normalize the matrixes $\mathbf{W_P}$, $\mathbf{W_N}$, each element should be divided by the sum of all elements in the matrix. The sum of all elements in matrix $\mathbf{W_P}$ is parallel computed as follows. First, each process calculates the sum of all elements in the submatrix ${\mathbf{W_P}}^i$ independently. We denote the sum of all elements in the submatrix $\mathbf{W_P}$ and ${\mathbf{W_P}}^i$ as $\mathcal{S}$ and $\mathcal{S}^i$, respectively. Then we compute the sum of all elements in matrix $\mathbf{W_P}$ by $\mathcal{S} = \sum_{i=1}^P \mathcal{S}_i$ through an MPI_Allgather action. Here all-to-all communication happens, and the communication volume for each process is $\mathcal{O}(P)$. Then, we normalize matrix $\mathbf{W_P}$ in each process by taking ${\mathbf{W_P}}^i = {\mathbf{W_P}}^i/\mathcal{S}$ in parallel without communication. The normalization of matrix $\mathbf{W_N}$ is done in a similar way.

Computation of low-dimensional embedding X

After normalizing $\mathbf{W_P}$, its Laplacian $\mathbf{L_P}$, which is needed for the subsequent determination of descent direction, is computed in parallel as follows. In the i-th process, we calculate the elements of submatrix ${\mathbf{L_P}}^i$ by using $l^+_{mn} = \sum_{k=1}^N w^+_{mk} - w^+_{mn}$, where l^+_{mn} and w^+_{mn} are the elements of matrixes $\mathbf{L_P}$ and $\mathbf{W_P}$, respectively. Since the two matrixes are partitioned by row in the same way, the computation of $\mathbf{L_P}$ is also communication-free.

The low-dimensional embedding $\mathbf{X}$ is obtained by solving the optimization problem with the Partial-Hessian optimization strategy. During the Quasi-Newton procedures, a dense linear system $\mathbf{L_P}\mathbf{P_k} = -\mathbf{G_k}$ must be solved in parallel. In the D-EE algorithm, we perform LU decomposition on $\mathbf{L_P}$. Considering that $\mathbf{L_P}$ is positive semi-definite, but not positive definite, a small value μ is added to the diagonal of $\mathbf{L_P}$ in practice. During the following sections, we still use $\mathbf{L_P}$ to denote the adjusted matrix. LU decomposition on $\mathbf{L_P} = \mathcal{L}\mathcal{U}$ is done with PETSc, which provides uniform and efficient access to all linear system solvers in the package, including parallel and sequential, direct and iterative [14, 15, 16]. Here, $\mathcal{L}$ and $\mathcal{U}$ are the corresponding lower and upper triangle matrixes, respectively. With the decomposition of LU, the dense linear system $\mathbf{L_P}\mathbf{P_k} = -\mathbf{G_k}$ is replaced by two sublinear systems $\mathcal{L}\hat{\mathbf{P}}_\mathbf{k} = -\mathbf{G_k}$ and $\mathcal{U}\mathbf{P_k} = \hat{\mathbf{P}}_\mathbf{k}$, which can be solved by the backward substitution method.

As shown in Figure 1D, the partitions of $\mathbf{L_P}$, $\mathcal{L}$, and $\mathcal{U}$ are the same as $\mathbf{W_P}$. Let us denote $\mathbf{P_k} = [{\mathbf{P_k}}^1, \cdots, {\mathbf{P_k}}^P]$, where submatrix ${\mathbf{P_k}}^i$ with size of $M_i \times d$ is stored in the i-th process, and a similar partition is performed on $\mathbf{G_k}$. Based on the partitions of $\mathbf{L_P}$, $\mathcal{L}$, $\mathcal{U}$, $\mathbf{P_k}$, and $\mathbf{G_k}$, the computational complexities per process of LU decomposition and backward substitution are $\mathcal{O}(N^3/P)$ and $\mathcal{O}(N^2/P)$, with corresponding communication volumes of $\mathcal{O}(N^2/P)$ and $\mathcal{O}(N/P)$, respectively. According to the analysis, LU decomposition is only done in the first iteration of the Quasi-Newton method, and matrixes $\mathcal{L}$ and $\mathcal{U}$ are stored and reused during the whole Quasi-Newton procedure.

The gradient $\mathbf{G_k}$ in the right-hand side of the linear system $\mathbf{L_P}\mathbf{P_k} = -\mathbf{G_k}$ is calculated by $\mathbf{G_k} = \mathbf{L_k}\mathbf{X_k}$, where the $N \times N$ matrix $\mathbf{L_k}$ depends on matrixes $\mathbf{W_P}$, $\mathbf{W_N}$, and $\mathbf{Ker}$. Here the elements of matrix $\mathbf{Ker}$ are defined as $\mathrm{ker}_{mn} = \exp(-\|x_m - x_n\|^2)$, and the elements of matrix $\mathbf{L_k}$ are defined as $l^{(k)}_{mn} = w^+_{mn} - \lambda w^-_{mn} \mathrm{ker}^{(k)}_{mn}$. As shown in Figure 1E, the partitions of matrixes $\mathbf{L_k}$ and $\mathbf{Ker}$ are the same as those of $\mathbf{W_P}$. In D-EE, we store all elements of $\mathbf{X_k}$ in each process, which is the same as the original high-dimensional data $\mathbf{Y}$. Thus, we can parallel compute matrixes $\mathbf{L_k}$ and $\mathbf{Ker}$ in the same way with the matrix $\mathbf{W_N}$, which means the procedure is also communication-free.

After solving the linear system, we obtain the search direction $\mathbf{P_k}$. Then, we update $\mathbf{X_{k+1}}$ according to $\mathbf{X_{k+1}} = \mathbf{X_k} + \alpha_k\mathbf{P_k}$, where α_k is determined by a line search approach. As men-

tioned before, the low-dimensional embedding $\mathbf{X_k}$ is stored sequentially, but $\mathbf{P_k}$ is distributed stored. Thus, we first compute the elements of submatrix $\mathbf{P_{k+1}}^i$ in the i-th process and then gather all elements of $\mathbf{P_k}$ in each process by the all-gather function in MPI. Here all-to-all communication happens, and the order of communication volume for each process is $\mathcal{O}(Nd)$. In line search steps, we need to calculate the energy function $E(\mathbf{X}, \lambda)$ several times, which is computed in parallel according to the following formula

$$E(\mathbf{X}, \lambda) = \sum_{i=1}^{P} \left(\sum_{m=\mathcal{M}_i+1}^{\mathcal{M}_{i+1}} \sum_{n=1}^{N} \left\{ w_{mn}^{+} \|x_n - x_m\|^2 + \lambda w_{mn}^{-} \exp(-\|x_n - x_m\|^2) \right\} \right).$$

The summation included in the parentheses is calculated in each process simultaneously and then gathered by the MPI all-gather function. Here all-to-all communication happens, and the communication volume for each process is $\mathcal{O}(P)$.

Results

Data Description

We test the accuracy and scalability of D-EE on three datasets. The first simulated dataset [17], named PHATE data for convenience, consists of 1,440 samples and 60 features. It is a complex tree structure which simulates a cellular developmental process, namely, progressions, branch or split in progressions and end state of progression, composed of ten branches in total. We first perform principal component analysis (PCA) on the original data, reserving a 1,440 samples $\times$ 7 features matrix.

The second dataset characterizes process of mouse hematopoietic stem and progenitor cells (HSPCs) bifurcating to myeloid and erythroid precursors [18], consisting of 4,423 samples. The obtained single-cell read count data is pre-processed by Seurat package (version 3.2.1) [19, 20]. Firstly, we normalize the gene expression in each sample as follows: we divide each gene read count by the total read counts for each cell, and then multiply a scale factor of 10^4 and plus one, followed by taking a logarithmic transformation. Secondly, we select the top 2,000 variable genes using the default "vst" method of Seurat package, i.e., variance-stabilizing transformation [21]. Finally, we conduct PCA on the processed data, and select the top 50 largest principal components, resulting a 4,423 samples $\times$ 50 features matrix as input of EE and D-EE.

The third data is a large-scale time series scRNA-seq dataset containing ~250k cells [22]. The data characterizes reprogramming of fibroblasts to induced pluripotent stem cells (iPSCs), which was collected at half-day intervals across 18 days, resulting in 39 time points. Since the final time point of the iPSCs status was not annotated temporally, we therefore set the final point as 20-th day as the input to D-TSEE. We pre-process this data with Seurat package as well. Same as the pre-process of HSPCs data, we first filter cells and genes to include cells where at least 200 features are detected and to include genes detected in at least 50 cells, obtaining 259,081 cells and 19,427 genes. After that, we perform logarithmic transformation, select variable features and perform PCA as described in HSPCs dataset, obtaining a 259,081 samples $\times$ 50 features matrix as input of dimensionality reduction methods.

D-EE achieves high strong scaling efficiency

We evaluate D-EE using both simulated and real scRNA-seq datasets. The numerical tests are carried out on the LSSC-IV supercomputer. The 400 computing nodes of LSSC-IV are comprised of two 18-core Intel Xeon Gold CPUs with 192 GB local memory, and are interconnected via a proprietary high performance network. First, we employ PHATE data and HSPCs data to test the consistency between D-EE and EE results. We employ 36 processes in both D-EE algorithms. The low dimensions are set to 2 for the convenience of visualization for both datasets, and the parameter λ used is set to the default value 10. We use the same initialization generated from Gaussian distribution as that in the original EE Matlab code.

D-EE achieves consistent 2-dimensional embedding as that of EE (Figure 2). To measure the consistency quantitatively, we calculate the relative error which is defined by

$$\text{relative error} = \frac{\|A - B\|_F}{\|A\|_F},$$

where $\|\cdot\|_F$ is the Frobenius norm of matrix, A and B represent the output of EE and D-EE, respectively. Frobenius norm of a matrix $A \in \mathbb{R}^{m\times n}$ is defined as

$$\|A\|_F = \sqrt{\sum_{i=1}^{m}\sum_{j=1}^{n} |a_{ij}|^2}.$$

Their relative errors of D-EE in HSPCs dataset and PHATE dataset are 2.42×10^{-6} and 1.60×10^{-6}, respectively, thus further validating the consistency of results by D-EE and EE.

To test performance of parallel efficiency of D-EE on the large-scale dataset, we apply D-EE to the iPSCs dataset (~ 250k cells) using 500, 1,000, 2,000, and 4,000 processes, respectively, and for each setting of number of processes we run at least twice. The averaged computation times of D-EE for iPSCs dataset are 5.83 hours, 3.19 hours, 2.36 hours, and 2.02 hours, when the numbers of processes are 500, 1,000, 2,000, and 4,000, respectively. Next, we evaluate the parallel performance of D-EE based on two widely used indexes, the strong scaling speedup ratio and the parallel efficiency. The strong speedup ratio is defined as

$$S = \frac{T_s}{T_p},$$

where T_s and T_p are the time of computation by using single process and p processes, respectively. The parallel efficiency is defined as

$$E = \frac{S}{p} = \frac{T_s}{pT_p}.$$

The ideal strong speedup should be p, and the corresponding parallel efficiency should be 1 when p processes are used. However, it is impossible to run the data with 250k samples on a single process owing to limited memory and low efficiency of EE algorithm. Thus, we take the time of computation of 500 processes as T_s with $s = 500$, and the parallel efficiency is then reformulated as

$$E = \frac{sT_s}{pT_p}.$$

When adopting the speedup ratio and parallel efficiency as the indexes for scaling, a strong scaling performance at remarkable speedup is observed when increasing CPUs from 500 to 4,000 processes (Figure 3A, B).

We further evaluate the performance of D-EE on computational times in our supercomputer. Three test cases with sample sizes being 10k, 50k, and 100k are run on 500, 1,000, and 2,000 processors, respectively. It is shown that when using the same number of processes, it is naturally that the computational time increases as total sample size increases (as shown in Figure 3C).

In practice, for analysis of large-scale single-cell dataset, a workstation with multiple CPUs and large memory is recommended. Meanwhile, we also test D-EE on datasets at different sample sizes, and find that D-EE efficiently implements on a typical personal computer (e.g., 8 CPU threads, 16 Gb RAM) with up to a sample size of 12k, while on a conventional workstation (e.g., 40 CPU threads, 256 Gb RAM) with up to a sample size of 48k, respectively.

D-EE and D-TSEE recover intrinsic low-dimensional structures of large-scale scRNA-seq data

We illustrate the application of D-EE and D-TSEE on the large-scale iPSCs dataset. We visualize the iPSCs data on the 2-dimensional space using t-SNE, UMAP, D-EE and D-TSEE, respectively (Figure 4). The same pre-processed single-cell data is used for all four methods. The t-SNE is conducted by the FIt-SNE method [23] with a PCA initialization, and we choose the learning rate as 1/12 of the sample size, according to [3] for better preservation of the global structures. UMAP is conducted by adjusting the number of nearest neighbors (NNs) to balance the preservation of local and global structures. We find that UMAP is not sensitive when choosing the number of NNs from 30 (defaults) to 500 (square root of the number of samples), and we thus set the number of NNs to be 100 in our study. Both t-SNE and UMAP are implemented by the Seurat software (version 3.2.1). In our implementations, both D-EE and D-TSEE are used with their default parameters.

We color the cells based on time stages on their low-dimensional embeddings obtained by the four dimensionality reduction results (Figure 4). We find that t-SNE preserves the time lineage structure of data in an “S” shape with small gaps (Figure 4, upper-left panel); UMAP also shows a time lineage structure, but with a large gap occurring between time stages 5.5 and 6 (Figure 4, upper-right panel). In contrast, both D-EE and D-TSEE preserve continuous time lineage structures in low-dimensional space (Figure 4, lower panels).

We further explore the gene expression patterns of Sox2, Sox4 and Nanog on both D-EE and D-TSEE embeddings (Figure 5). These genes are key regulators during stem cell differentiation and reprogramming process [24, 25, 26]. Previous study has shown evidence that these genes may oscillate during cell development progression [11, 26]. These genes display oscillatory gene expression patterns in the early stage of iPSCs on the D-TSEE view (Figure 5), providing useful information and clues for downstream analysis.

Conclusion

In this work, we develop a novel tool, D-EE, for visualizing large-scale scRNA-seq data. D-EE implements the distributed storage and distributed computing techniques to a powerful nonlinear dimensionality reduction method, Elastic Embedding. The optimal distributed computational strategies implemented by D-EE allow it to achieve not only the strong scalability on large-scale dataset, but also the exact optimization solution as original EE by fully utilizing the whole data. Numerical experiments validate the correctness and parallel efficiency of D-EE. Considering the emergence of time series scRNA-seq data, our D-TSEE tool allows us efficiently to perform dimensionality reduction on large-scale single-cell data by employing experimentally temporal information. Besides, when incorporating temporal information if it is available, D-TSEE can reveal dynamic gene expression patterns, providing insights for subsequent analysis of molecular mechanisms and dynamic transition progression.

We demonstrate that D-EE and D-TSEE work efficiently on large-scale datasets at a super computer. However, the proposed distributed algorithm D-EE still has disadvantage due to the huge computational cost and storage with a relative large number of cells. Therefore, D-EE is limited to handle and analyze huge-scale datasets with the number of cells up to the order of millions [27]. In comparison, the state-of-the-art accelerated implementations of t-SNE (e.g., FIt-SNE) and UMAP are of the close-to-linear computational complexities, showing great efficiency on huge data analysis.

In the future study, to resolve the limitation of D-EE on huge-scale data computation, we can accelerate D-EE by adopting either the fast Fourier transform as used in FIt-SNE, or adopting the state-of-the-art neural network framework used by net-SNE [28]. On the other hand, since huge-scale single-cell dataset can be highly redundant, we can also select subset of informative samples using the advanced geometric sketching tool [29] prior to application of D-EE.

Availability of source code and requirements

Lists the following:

- Project name: D-EE
- Project home page: `https://github.com/ShaokunAn/D-EE`
- Operating system(s): Linux
- Programming language: C, R
- Other requirements: Multi-core processor, implementation of MPI library (i.e., OpenMPI or IntelMPI) installed on each node of the cluster, a reasonably fast interconnecting infrastructure, PETSc 3.11.4 or higher
- License: GNU General Public License
- biotools: d-ee

Availability of supporting data and materials

The PHATE data supporting the results of this article is available in the Github repository [17]. The iPSCs data is available in NCBI repository with number GSE 122662 [22]. The HPSCs data is available in the NCBI repository with accession number GSE72857 and the dataset used in our study is downloaded from their Github `https://github.com/ManuSetty/wishbone`.

Declarations

List of abbreviations

D-EE: distributed optimization implementation of Elastic Embedding; D-TSEE: distributed optimization implementation of time series Elastic Embedding; EE: Elastic Embedding; scRNA-seq: single-cell RNA sequencing; PCA: Principal Component Analysis; TSEE: time series Elastic Embedding; t-SNE: t-distributed Stochastic Neighbor Embedding; UMAP: Uniform Manifold Approximation and Projection; NNs: nearest neighbors

Consent for publication

Not applicable.

Competing Interests

The authors declare that they have no competing interests.

Funding

This work is supported by the National Key R&D Program of China under Grant 2018YFB0704304, NSFC grants (Nos. 11871069, 12071466), NCMIS of CAS, LSEC of CAS, LSC of CAS, and the Youth Innovation Promotion Association of CAS.

Author's Contributions

Conceptualization and Methodology: S.A., L.W. Software: S.A., J.H. Supervision: S.A., L.W., J.H. Funding Acquisition: L.W., J.H. Writing - Original Draft Preparation: S.A. Writing - Review & Editing: all authors.

References

1. Hie B, Peters J, Nyquist SK, Shalek AK, Berger B, Bryson BD. Computational Methods for Single-Cell RNA Sequencing. Annual Review of Biomedical Data Science 2020 2020/08/03;3(1):339–364. https://doi.org/10.1146/annurev-biodatasci-012220-100601.
2. van der Maaten LJP, Hinton GE. Visualizing High-Dimensional Data Using t-SNE. Journal of Machine Learning Research 2008;9:2579–2625.
3. Kobak D, Berens P. The art of using t-SNE for single-cell transcriptomics. Nature Communications 2019;10(1):5416. https://doi.org/10.1038/s41467-019-13056-x.
4. Carreira-Perpiñán MÁ. The Elastic Embedding Algorithm for Dimensionality Reduction. In: Proceedings of the 27th International Conference on Machine Learning (ICML-10), June 21–24, 2010, Haifa, Israel; 2010. p. 167–174. http://www.icml2010.org/papers/123.pdf.
5. Chen Z, An S, Bai X, Gong F, Ma L, Wan L. DensityPath: an algorithm to visualize and reconstruct cell state-transition path on density landscape for single-cell RNA sequencing data. Bioinformatics 2019 4;35(15):2593–2601.
6. Nguyen LH, Holmes S. Ten quick tips for effective dimensionality reduction. PLOS Computational Biology 2019 06;15(6):1–19. https://doi.org/10.1371/journal.pcbi.1006907.
7. McInnes L, Healy J, Melville J. UMAP: Uniform Manifold Approximation and Projection for Dimension Reduction. arXiv e-prints 2018 Feb;p. arXiv:1802.03426.
8. Becht E, McInnes L, Healy J, Dutertre CA, Kwok IWH, Ng LG, et al. Dimensionality reduction for visualizing single-cell data using UMAP. Nature Biotechnology 2019;37(1):38–44. https://doi.org/10.1038/nbt.4314.
9. Kobak D, Linderman GC. UMAP does not preserve global structure any better than t-SNE when using the same initialization. bioRxiv 2019;https://www.biorxiv.org/content/early/2019/12/19/2019.12.19.877522.
10. Wasserman L. Topological Data Analysis. Annual Review of Statistics and Its Application 2018;5(1):501–532. https://doi.org/10.1146/annurev-statistics-031017-100045.
11. An S, Ma L, Wan L. TSEE: an elastic embedding method to visualize the dynamic gene expression patterns of time series single-cell RNA sequencing data. BMC Genomics 2019;20(2):224. https://doi.org/10.1186/s12864-019-5477-8.
12. Vladymyrov M, Carreira-Perpinan M. Entropic Affinities: Properties and Efficient Numerical Computation. In: Dasgupta S, McAllester D, editors. Proceedings of the 30th International Conference on Machine Learning, vol. 28 of Proceedings of Machine Learning Research Atlanta, Georgia, USA: PMLR; 2013. p. 477–485. http://proceedings.mlr.press/v28/vladymyrov13.html.
13. Vladymyrov M, Carreira-Perpinan M. Partial-Hessian Strategies for Fast Learning of Nonlinear Embeddings. arXiv e-prints 2012 Jun;p. arXiv:1206.4646.
14. Balay S, Abhyankar S, Adams MF, Brown J, Brune P, Buschelman K, et al., PETSc Web page; 2019. https://www.mcs.anl.gov/petsc. https://www.mcs.anl.gov/petsc.
15. Balay S, Abhyankar S, Adams MF, Brown J, Brune P, Buschelman K, et al. PETSc Users Manual. Argonne National Laboratory; 2019.
16. Balay S, Gropp WD, McInnes LC, Smith BF. Efficient Management of Parallelism in Object Oriented Numerical Software Libraries. In: Arge E, Bruaset AM, Langtangen HP, editors. Modern Software Tools in Scientific Computing Birkhäuser Press; 1997. p. 163–202.
17. Moon KR, van Dijk D, Wang Z, Gigante S, Burkhardt DB, Chen WS, et al. Visualizing structure and transitions in high-dimensional biological data. Nature Biotechnology 2019;37(12):1482–1492. https://doi.org/10.1038/s41587-019-0336-3.
18. Setty M, Tadmor MD, Reich-Zeliger S, Angel O, Salame TM, Kathail P, et al. Wishbone identifies bifurcating developmental trajectories from single-cell data. Nature Biotechnology 2016;34(6):637–645. https://doi.org/10.1038/nbt.3569.
19. Butler A, Hoffman P, Smibert P, Papalexi E, Satija R. Integrating single-cell transcriptomic data across different conditions, technologies, and species. Nature Biotechnology 2018;36(5):411–420. https://doi.org/10.1038/nbt.4096.
20. Stuart T, Butler A, Hoffman P, Hafemeister C, Papalexi E, Mauck I William M, et al. Comprehensive Integration of Single-Cell Data. Cell 2019 2020/08/03;177(7):1888–1902.e21. https://doi.org/10.1016/j.cell.2019.05.031.
21. Hafemeister C, Satija R. Normalization and variance stabilization of single-cell RNA-seq data using regularized negative binomial regression. Genome Biology 2019;20(1):296. https://doi.org/10.1186/s13059-019-1874-1.
22. Schiebinger G, Shu J, Tabaka M, Cleary B, Subramanian V, Solomon A, et al. Optimal-Transport Analysis of Single-Cell Gene Expression Identifies Developmental Trajectories in Reprogramming. Cell 2019 2020/02/05;176(4):928–943.e22. https://doi.org/10.1016/j.cell.2019.01.006.
23. Linderman GC, Rachh M, Hoskins JG, Steinerberger S, Kluger Y. Fast interpolation-based t-SNE for improved visualization of single-cell RNA-seq data. Nature Methods 2019;16(3):243–245. https://doi.org/10.1038/s41592-018-0308-4.
24. Seo E, Basu-Roy U, Gunaratne PH, Coarfa C, Lim DS, Basilico C, et al. SOX2 Regulates YAP1 to Maintain Stemness and Determine Cell Fate in the Osteo-Adipo Lineage. Cell Reports 2013;3(6):2075–2087. http://www.sciencedirect.com/science/article/pii/S2211124713002465.
25. Hanieh H, Ahmed EA, Vishnubalaji R, Alajez NM. SOX4: Epigenetic regulation and role in tumorigenesis. Seminars in Cancer Biology 2019;http://www.sciencedirect.com/science/article/pii/S1044579X18301809.
26. Yu P, Nie Q, Tang C, Zhang L. Nanog induced intermediate state in regulating stem cell differentiation and reprogramming. BMC Systems Biology 2018;12(1):22. https:

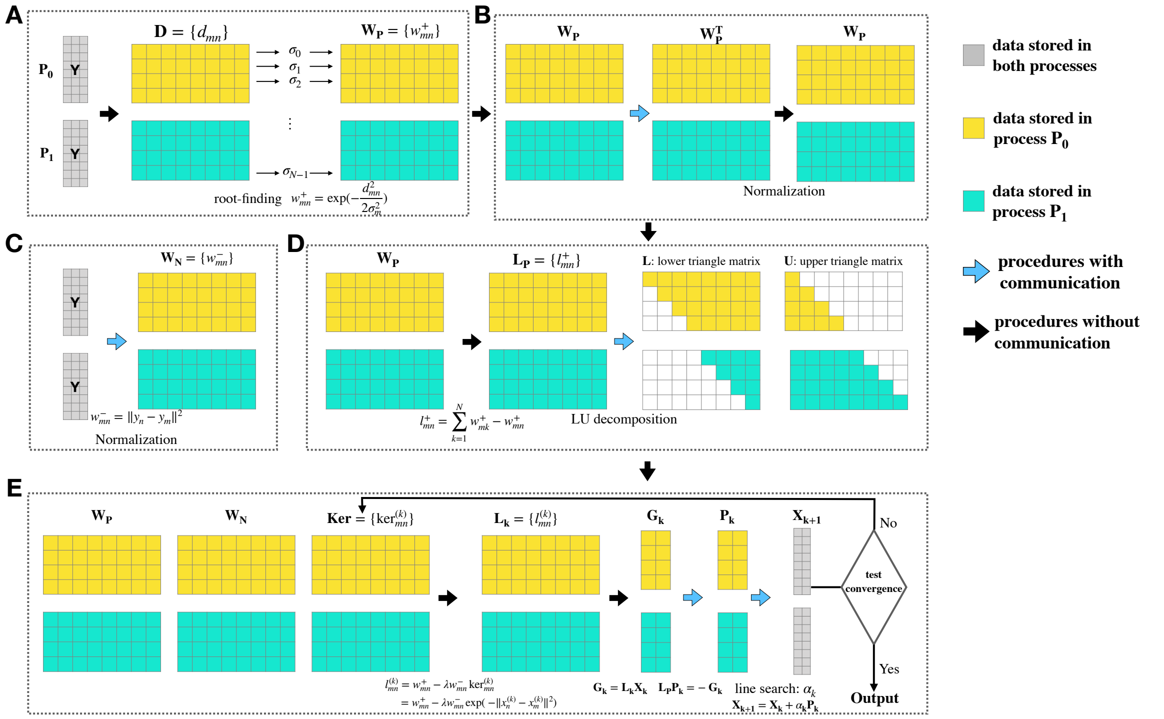

Figure 1. Overview of D-EE algorithm. The D-EE algorithm can be decomposed into five parts. Part A: the computation of matrixes $\mathbf{D}$ and $\mathbf{W_P}$. To obtain $\mathbf{W_P}$, the parameters $\{\sigma_n\}$ are determined by solving a series of root-finding problems. Part B: the symmetry and normalization of the matrix $\mathbf{W_P}$. Part C: the computation and normalization of $\mathbf{W_N}$. Part D: the computation of the Laplacian matrix $\mathbf{L_P}$ together with LU decomposition. Part E: the computation of $\mathbf{X_{k+1}}$ by solving an optimization problem with the classic Quasi-Newton methods iteratively. In the *k-th* iteration, $\mathbf{L_k}$ is computed based on $\mathbf{W_P}$, $\mathbf{W_N}$ and $\mathbf{X_k}$, which are used to obtain the gradient $\mathbf{G_k} = \mathbf{L_k X_k}$. The descent direction $\mathbf{P_k}$ is then determined by solving a linear system $\mathbf{L_P P_k} = -\mathbf{G_k}$. Finally, $\mathbf{X_{k+1}}$ is updated according to $\mathbf{X_{k+1}} = \mathbf{X_k} + \alpha_k \mathbf{P_k}$, where the step size α_k is calculated by a line search method.

//doi.org/10.1186/s12918-018-0552-3.

27. Cao J, Spielmann M, Qiu X, Huang X, Ibrahim DM, Hill AJ, et al. The single-cell transcriptional landscape of mammalian organogenesis. Nature 2019;566(7745):496–502. https://doi.org/10.1038/s41586-019-0969-x.
28. Cho H, Berger B, Peng J. Generalizable and Scalable Visualization of Single-Cell Data Using Neural Networks. Cell Systems 2018 2020/02/05;7(2):185–191.e4. https://doi.org/10.1016/j.cels.2018.05.017.
29. Hie B, Cho H, DeMeo B, Bryson B, Berger B. Geometric Sketching Compactly Summarizes the Single-Cell Transcriptomic Landscape. Cell Systems 2019 2020/09/24;8(6):483–493.e7. https://doi.org/10.1016/j.cels.2019.05.003.

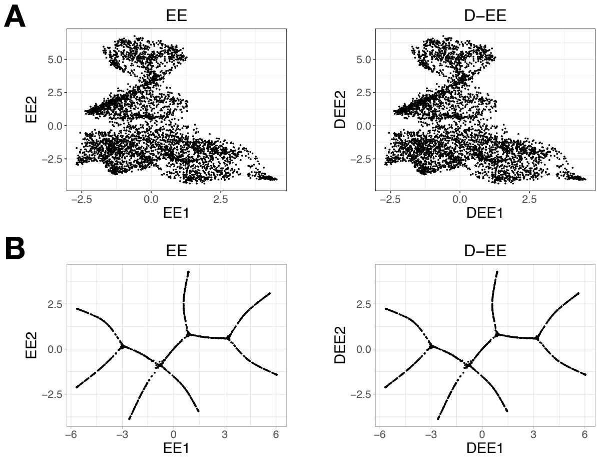

Figure 2. D-EE and EE achieve the same results on two datasets when using the same initializations. A: The 2-D mapping of HSPCs data obtained by the two algorithms. B: The 2-D mapping of PHATE data obtained by the two algorithms.

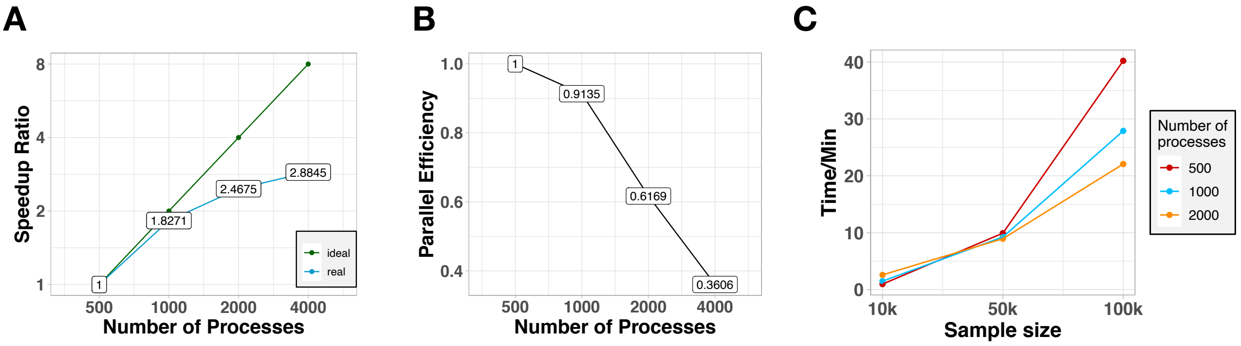

Figure 3. Strong scaling results and parallel efficiency of the D-EE algorithm on an LSSC-IV supercomputer. We apply D-EE on iPSCs dataset by using 500, 1,000, 2,000, and 4,000 processes, respectively. A: The strong speedup ratio increases with increase in the number of processes.The green line represents the ideal speedup ratio and the blue line represents the speedup ratio obtained by D-EE. B: The parallel efficiency decreases at an acceptable rate with the increase in number of processes. C: Computational times consumed for 10k, 50k, 100k samples under 500, 1000 and 2000 processes, respectively.

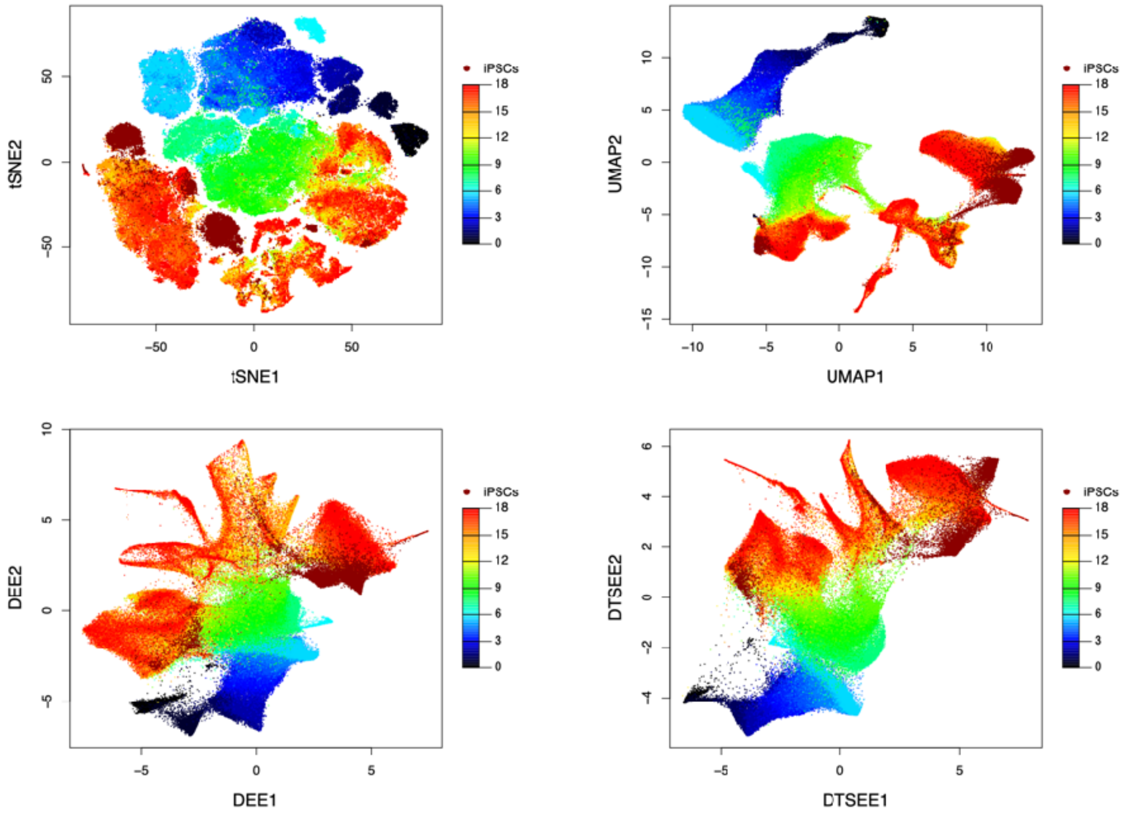

Figure 4. Cells are colored by time stages in iPSCs dataset on the 2-dimensional space obtained by four dimensionality reduction methods, i.e., t-SNE, UMAP, D-EE and D-TSEE.

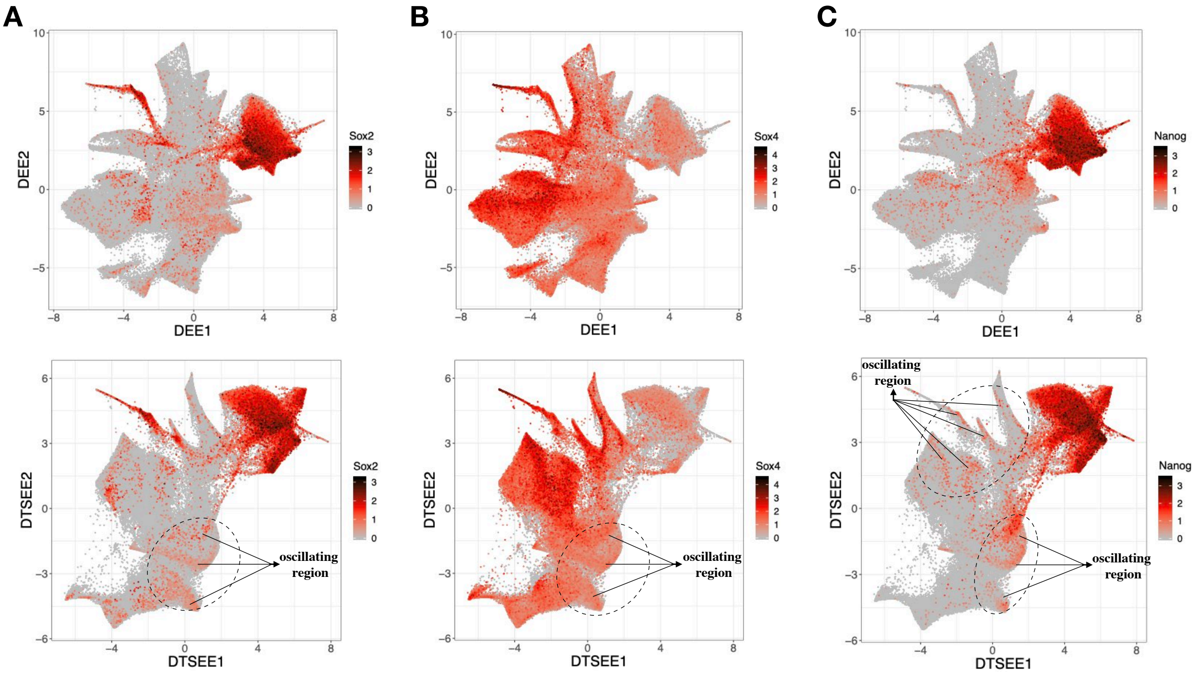

Figure 5. Cells are colored by gene expression of Sox2, Sox4, and Nanog in iPSCs dataset on the 2-dimensional embeddings obtained by D-EE and D-TSEE, respectively.

Personal Cover Click here to access/download;Personal Cover;Cover Letter.docx

Lin Wan, Ph.D., Jizu Huang, Ph.D., Shaokun An

Academy of Mathematics and Systems Science

Chinese Academy of Sciences

No. 55, Zhongguancun East Rd.,

Beijing, 101900, P. R. China

Tel: (8610) 8254-1620

Email: lwan@amss.ac.cn huangjz@lsec.cc.ac.cn anshaokun@amss.ac.cn

GigaScience

Oxford University Press

September 29, 2020

Dear Editor,

We are pleased to submit our revised manuscript "D-EE: software for visualizing low-dimensional intrinsic structures of large-scale single-cell RNA-sequencing data" for consideration for *GigaScience*.

Thank you for your valuable suggestion, we registered our proposed software with both bio.tools and SciCrunch.org databases, and provided the biotools ID identifiers in our revised manuscript.

We are also very grateful to the referees' insightful comments and suggestions. They are very helpful for us to improve the research and to revise the manuscript. The manuscript has been fully revised by following the reviewers' comments and suggestions, making our results more solid and convincing. We summarize the major changes as follows:

1) As suggested by Reviewer #1 and #2, in the revised manuscript, we tuned the parameters of t-SNE and UMAP and provided detailed implementation information of t-SNE and UMAP. We found that their performances can be further improved, especially for t-SNE (see the first paragraph of subsection "D-EE and D-TSEE recover intrinsic low-dimensional structures of large-scale scRNA-seq data" on Page 5). We also toned down our claims and removed the conclusion that "EE outperforms t-SNE and UMAP".

2) As suggested by Reviewer #1 and #2, in the revised manuscript, we extensively tested our proposed software on running time and shown the new results in Figure 3C. We also made crystal discussion on the constant time speedup of our method at the second paragraph of Conclusion on Page 5.

3) As suggested by Reviewer #1 and #2, in the revised manuscript, we provided more information on the hardware requirements of our proposed method at subsection “D-EE achieves high strong scaling efficiency” at Pages 4-5.

4) We revised several parts in Introduction, Methods, Conclusion, to make them clearer.

5) Besides, we revised the figures, and made many minor changes to improve the presentation according to the reviewers’ comments and suggestions.

We prepared the point-to-point response letters to each reviewer in a separate file. We wish that these revisions were satisfactory. If there is any problem with the manuscript or files, please let us know.

Yours sincerely,

Jizu Huang, Lin Wan

Academy of Mathematics and Systems Science & Beijing Institute of Genomics
Chinese Academy of Sciences
