# Supplementary material for: D-EE: Distributed software for visualizing intrinsic structure of large-scale single-cell data
Source: Gigascience. 2020 Nov 11;9(11):giaa126. doi: 10.1093/gigascience/giaa126 (PMC7657844; doi:10.1093/gigascience/giaa126)
Supplement: giaa126_Response_to_Reviewer_Comments_Original_Submission [file giaa126_response_to_reviewer_comments_original_submission.pdf]

Author's Response To Reviewer Comments

Close

Response to reviewers:

Reply:
We would like to thank the reviewers for thoughtful comments and valuable suggestions on our manuscript. The manuscript has been fully revised by following the reviewers' comments and suggestions, making our results more solid and convincing. We hope the new version will meet the criteria of GigaScience. The point-by-point responses to the reviewers' comments and suggestions are as follows.

Reviewer #1:

Does the manuscript adhere to the journal's guidelines on minimum standards of reporting?
Yes
General comments: The authors propose a distributed modification to Elastic Embedding (EE) approaches that provide a multiprocessing speedup to visualization of scRNA-seq data. The manuscript is well written, is based on sound techniques, and describes the overall procedure well. There are some places in which the authors do overclaim or omit important information, so the manuscript would benefit from repositioning the text of the paper accordingly. Overall, I am supportive of publication of the manuscript.

Reply:
We thank the reviewer for summarizing our work and also the thoughtful comments. We give the point-to-point response below.

Comment:
1) Are the methods appropriate to the aims of the study, are they well described, and are necessary controls included?

The math and methodological description of the method is done clearly, which is very much appreciated. Providing GitHub code with instructions for reproducibility is also great.

Reply:
We thank the reviewer for the thoughtful comments.

Comment:
2) Are the conclusions adequately supported by the data shown?

There are some areas in which the manuscript makes claims that should be toned down. Or, if the authors wish to make these claims, then they would need additional analytic support.

The paper claims that EE "outperforms t-SNE and UMAP" in preserving global structures. Such a claim would need to be supported by a much more exhaustive consideration of different visualization parameters. In particular, for UMAP, I might suspect that with different parameters (e.g., a higher nearest neighbor parameter), the "global structure" would not be broken. Instead, the authors could just say something about how well their own method works without the relative comparison, and still provide single instances of t-SNE and UMAP for a visual comparison.

Reply:
Thanks for your thoughtful comments. In the revised manuscript, we followed your advice and toned down the claims as follows.
(1) We removed all the claims that "EE outperforms t-SNE and UMAP" in the previous version, and merely provided their visual comparisons on iPSCs data (see the second paragraph of subsection "D-EE

and D-TSEE recover intrinsic low-dimensional structures of large-scale scRNA-seq data" on Page 5 and Figure 4).
(2) We followed your suggestion and tuned the parameters of t-SNE and UMAP. We found that their performances can be further improved, especially for t-SNE. A short paragraph was added in the revised manuscript as follows. "…The t-SNE is conducted by the FIt-SNE method [23] with a PCA initialization, and we choose the learning rate as 1/12 of the sample size, according to [3] for better preservation of the global structures. UMAP is conducted by adjusting the number of nearest neighbors (NNs) to balance the preservation of local and global structures. We find that UMAP is not sensitive when choosing the number of NNs from 30 (defaults) to 500 (square root of the number of samples), and we thus set the number of NNs to be 100 in our study. Both t-SNE and UMAP are implemented by the Seurat software (version 3.2.1)..." (see the first paragraph of subsection "D-EE and D-TSEE recover intrinsic low-dimensional structures of large-scale scRNA-seq data" on Page 5.)

Comment:
The authors should also make crystal clear that the speedup is effectively only a constant time one, which may be limiting since implementations of t-SNE (e.g., Barnes-Hut t-SNE or flt-SNE) and UMAP can run in close-to-linear time, which is a big practical win for those methods. In contrast, O(N^2/P) is still large when N = 2 million (e.g., the dataset in Cao et al., Nature, 2019). There's also no speed comparison between distributed EE and, e.g., UMAP. The authors need to make this point clear.

Reply:
Thanks for pointing out this issue. We agree well with you that the implementation of D-EE can be limited due to the constant time speedup.
In the revised manuscript:
Firstly, we made crystal discussion of this issue as: "We demonstrate that D-EE and D-TSEE work efficiently on large-scale datasets at a super computer. However, the proposed distributed algorithm D-EE still has disadvantage due to the huge computational cost and storage with a relative large number of cells. Therefore, D-EE is limited to handle and analyze huge-scale datasets with the number of cells up to the order of millions [27]. In comparison, the state-of-the-art accelerated implementations of t-SNE (e.g., FIt-SNE) and UMAP are of the close-to-linear computational complexities, showing great efficiency on huge data analysis." (see the second paragraph of Conclusion on Page 5)
Secondly, we pointed out that we can further improve D-EE to handle huge-scale datasets as follows. "In the future study, to resolve the limitation of D-EE on huge-scale data computation, we can accelerate D-EE by adopting either the fast Fourier transform as used in FIt-SNE, or adopting the state-of-the-art neural network framework used by net-SNE [28]. On the other hand, since huge-scale single-cell dataset can be highly redundant, we can also select subset of informative samples using the advanced geometric sketching tool [29] prior to application of D-EE." (see the last paragraph of Conclusion on Page 5)
Thirdly, we explored the relationship of runtime consumptions versus the sample sizes, showed in Figure 3C, and described the results as "We further evaluate the performance of D-EE on computational times in our supercomputer. Three test cases with sample sizes being 10k, 50k, and 100k are run on 500, 1,000, and 2,000 processors, respectively. It is shown that when using the same number of processes, it is naturally that the computational time increases as total sample size increases (as shown in Figure 3C). " (see the first paragraph on Page 5).

Comment:
3) Please indicate the quality of language in the manuscript. Does it require a heavy editing for language and clarity?

Language quality is good. One minor nitpick is to avoid contractions, e.g., "it's," in formal writing.

Reply:
Thanks for your pointing out this. We have corrected these contractions in the revised manuscript.

Comment:
4) Are you able to assess all statistics in the manuscript, including the appropriateness of statistical tests used?

Yes

Reviewer #2:
The paper by An et al. presents a distributed implementation of the elastic embedding (EE) algorithm that allows to run EE on large datasets. The authors argue that EE (and its time-series version TSEE, previously developed by the same authors) is a good choice of an embedding algorithm for single-cell data, and so their implementation allows to actually use it in practice. Previously existing implementations did not scale beyond a sample size of a few thousand cells.

This is a solid work on implementing a parallelized/distributed version of EE. The EE/TSEE algorithms can indeed be an attractive choice of an embedding algorithm for single-cell data, so this paper makes a contribution that is relevant for the field. Importantly, this paper does *not* suggest any new algorithm, but simply presents an efficient implementation of an existing algorithm. If this is in scope of the "Technical note" section of GigaScience, then I can recommend acceptance.

Reply:
Thanks for your thoughtful comments. We followed the instructions of GigaScience and submitted our manuscript to “Technical note” as our target, since “Technical Notes should present an open-source software tool or an experimental or computational method, test or procedure for the analysis or handling of large-scale data.”

Comment:
The main issue I have is that it seems the authors used a supercomputer to run their analysis, and it is not clear to me if this implementation would allow to analyze the same dataset (n=250k) on a normal desktop computer. For competitor algorithms like t-SNE and UMAP this dataset size is not a problem even on a laptop. If D-EE can only deal with this dataset on a supercomputer, then it's clearly much much less efficient than competitors. The authors should clarify this point, see also below.

MAJOR COMMENTS

* Even though the main contribution of the paper is efficient EE implementation, the authors do not provide almost any benchmarking results. What I would like to see reported in the text:

a) What exact hardware was used for the experiments? On page 4 the authors say that they used up to 4000 CPU processes. What kind of computer allows to run 4000 processes? Was it a computing cluster? Please give exact details of the used hardware. Caption of Figure 3 says it was a supercomputer, but more details should be given in the text.

Reply:
Thanks for your thoughtful comments. In the revised manuscript, we provided detailed information of the computing cluster on which we tested and implemented our software in main text as follows: “… The numerical tests are carried out on the LSSC-IV supercomputer. The 400 computing nodes of LSSC-IV are comprised of two 18-core Intel Xeon Gold CPUs with 192 GB local memory, and are interconnected via a proprietary high performance network. ” (see the first paragraph of “D-EE achieves high strong scaling efficiency” on Page 4)

Comment:
b) What was the actual runtime on this supercomputer? At a minimum, report the physical time for iPSCs dataset in the text (e.g. 1 hour, or 20 hours, or 30 minutes). Even better would be a figure (perhaps another panel in Figure 3) that plots the time as a function of sample size. What is the largest sample size that can be feasibly analyzed on the supercomputer?

Reply:
Thank you for your suggestion.
In the revised manuscript:
Firstly, we tested our software extensively at various numbers of processes on iPSCs dataset and provided the detailed runtime information: “To test performance of parallel efficiency of D-EE on the large-scale dataset, we apply D-EE to the iPSCs dataset (~ 250k cells) using 500, 1,000, 2,000, and 4,000 processes, respectively, and for each setting of number of processes we run at least twice. The averaged computation times of D-EE for iPSCs dataset are 5.83 hours, 3.19 hours, 2.36 hours, and 2.02 hours, when the number of processes are 500, 1,000, 2,000, and 4,000, respectively...” (see subsection

“D-EE achieves high strong scaling efficiency” on Page 4)
Secondly, we explored the relationship of runtime versus the sample sizes, displayed in Figure 3C, and described the results as “We further evaluate the performance of D-EE on computational times in our supercomputer. Three test cases with sample sizes being 10k, 50k, and 100k are run on 500, 1,000, and 2,000 processors, respectively. It is shown that when using the same number of processes, it is naturally that the computational time increases as total sample size increases (as shown in Figure 3C).” (see the first paragraph on Page 5)
Thirdly, we estimated the largest sample size that can be feasibly analyzed on the LSSC-IV supercomputer on which we implemented our software. Specifically, for a dataset with 10k samples implemented by using 8 processes, it consumed about 10 Gb memory. According to the memory of our supercomputer and the square relationship between the memory cost and the sample size N, we estimated the largest sample size that can be analyzed on our supercomputer is about 850k. We provided this information in the Instruction of D-EE at GitHub https://github.com/ShaokunAn/D-EE.

Comment:
c) Most importantly -- most users will not have access to a supercomputer (!!). The text should clearly state how large a dataset can be feasibly analyzed on a typical desktop computer (8 CPU threads, 16 Gb RAM) and perhaps on a typical powerful lab computing station (e.g. 40 CPU threads, 256 Gb RAM). Maybe include the runtimes using such hardware into the figure that I suggested above.

Reply:
Thanks for pointing out this problem. In the revised manuscript, we made it clear that a large computing cluster is recommended for the implementation of D-EE when analyzing large-scale datasets, and provided information about the largest sample size that can be feasibly analyzed on the two typical computing devices as follows: “In practice, for analysis of large-scale single-cell dataset, a workstation with multiple CPUs and large memory is recommended. Meanwhile, we also test D-EE on datasets at different sample sizes, and find that D-EE efficiently implements on a typical personal computer (e.g., 8 CPU threads, 16 Gb RAM) with up to a sample size of 12k, while on a conventional workstation (e.g., 40 CPU threads, 256 Gb RAM) with up to a sample size of 48k, respectively.” (see the second paragraph on Page 5)
For large sample size up to 250k as shown in our cases, D-EE has to be implemented on a super computer due to the large memory consumption. As mentioned in the revised manuscript, the improvement of D-EE is discussed as a future work: “In the future study, to resolve the limitation of D-EE on huge-scale data computation, we can accelerate D-EE by adopting either the fast Fourier transform as used in FIt-SNE, or adopting the state-of-the-art neural network framework used by net-SNE [28]. On the other hand, since huge-scale single-cell dataset can be highly redundant, we can also select subset of informative samples using the advanced geometric sketching tool [29] prior to application of D-EE.” (see the last paragraph of Conclusion section on Page 5)

Comment:
d) In case this implementation only works well on a supercomputer, and is not very helpful on a personal desktop computer, this should be stated very prominently in the abstract/intro/discussion/etc. Currently the abstract says that D-EE allows "to efficiently" analyze large RNAseq data. But if I must have a supercomputer for such an analysis, then it's not really very "efficient". The abstract should be clear about this. Note that t-SNE and UMAP can easily analyze n=250k on a desktop computer or a laptop. The authors have to discuss this in the Discussion.

Reply:
Thanks for your valuable suggestion. In the revised manuscript, we made it crystal clear in abstract that “…Its distributed storage and distributed computation technique allow us to efficiently analyze large-scale single-cell data at the cost of constant time speedup.”
We also discussed the limitation of D-EE in revised manuscript as “We demonstrate that D-EE and D-TSEE work efficiently on large-scale datasets at a super computer. However, the proposed distributed algorithm D-EE still has disadvantage due to the huge computational cost and storage with a relative large number of cells. Therefore, D-EE is limited to handle and analyze huge-scale datasets with the number of cells up to the order of millions [27]. In comparison, the state-of-the-art accelerated implementations of t-SNE (e.g., FIt-SNE) and UMAP are of the close-to-linear computational complexities, showing great efficiency on huge data analysis.” (see the second paragraph of Conclusion on Page 5).
Finally, in revised manuscript, we discussed potential improvement of D-EE for computation of huge

datasets: “In the future study, to resolve the limitation of D-EE on huge-scale data computation, we can accelerate D-EE by adopting either the fast Fourier transform as used in FIt-SNE, or adopting the state-of-the-art neural network framework used by net-SNE [28]. On the other hand, since huge-scale single-cell dataset can be highly redundant, we can also select subset of informative samples using the advanced geometric sketching tool [29] prior to application of D-EE.” (see the last paragraph of Conclusion section on Page 5)

Comment:
* The authors need to give exact details of how they ran t-SNE and UMAP for Figure 4 (page 4). Was it default parameters of some implementation? Name the implementation, give the version, say that it was default parameters. If you changed some parameters from the default ones, specify all of them.

In particular, for t-SNE I am worried that a random initialization was used for Figure 4. Random initialization is a bad idea, as stated e.g. in the cited Ref [8]. UMAP uses non-random init, so this is not a fair comparison. I suggest to use PCA initialization as explained in Ref [8] and also as is default in the latest versions of FIt-SNE and openTSNE.

Apart from that, t-SNE is sensitive to the learning rate (see https://www.nature.com/articles/s41467-019-13056-x), so it should be set appropriately, e.g. to n/12, which is also the default in FIt-SNE and openTSNE.

Reply:
Thank you for your valuable suggestion. In revised manuscript, we followed the instruction of the reference you provided, and tuned the parameters of both t-SNE and UMAP, and found that the performance of t-SNE can be largely improved. We added detailed implementation of t-SNE and UMAP as well as their version as follows: “The t-SNE is conducted by the FIt-SNE method [23] with a PCA initialization, and we choose the learning rate as 1/12 of the sample size, according to [3] for better preservation of the global structures. UMAP is conducted by adjusting the number of nearest neighbors (NNs) to balance the preservation of local and global structures. We find that UMAP is not sensitive when choosing the number of NNs from 30 (defaults) to 500 (square root of the number of samples), and we thus set the number of NNs to be 100 in our study. Both t-SNE and UMAP are implemented by the Seurat software (version 3.2.1). In our implementations, both D-EE and D-TSEE are used with their default parameters.” (see the first paragraph of subsection “D-EE and D-TSEE recover intrinsic low-dimensional structures of large-scale scRNA-seq data” on Page 5)

Comment:
* page 1 -- You might want to consider citing https://www.nature.com/articles/s41467-019-13056-x after "widely used in the single-cell community".

Reply:
Thanks for pointing out this valuable reference. In our revised manuscript, we cited the reference in 3 places:
(1) Introduction: “…For example, the celebrated t-distributed Stochastic Neighbor Embedding (t-SNE) algorithm [2] is widely used in the single-cell community [3].” (see Page 1)
(2) Methods: “…It is worth to note that, a newly proposed combinational perplexity has been applied to t-SNE [3], which greatly enhances the performance of t-SNE in preservation of global structures. The combinational perplexity can be also adopted by D-EE in future update.” (see Page 2)
(3) Results: “…The t-SNE is conducted by the FIt-SNE method [23] with a PCA initialization, and we choose the learning rate as 1/12 of the sample size, according to [3] for better preservation of the global structures.” (see Page 5)

Comment:
* page 2 -- "EE extends t-SNE", not sure this is a good way to put it. EE is not really an "extension" of t-SNE, is it? It's a different but very related algorithm. Consider revising.

Reply:
Thanks for your suggestion. In the revised manuscript, we modified the sentence as “…To achieve this goal, EE penalizes the placement of latent points in close proximity away from dissimilar data points in high-dimensional space…” (see Introduction on Page 2)

Comment:
• page 2, a bit later -- "the current implementations ... is" -> are

Reply:
Thanks for pointing this issue. We have corrected these typos in revised manuscript.

Comment:
• page 2, after the first formula: as far as I know, the original EE paper used one fixed value of sigma (kernel width) and not adaptive sigma_n as you do here. I suggest to mention this and explain/cite where the sigma_n comes from. Looks like it's from Ref [11] (last sentence in that paragraph). If so, write that you follow [11] in using adaptive sigma_n based on perplexity (?), and you use perplexity=X by default.

Reply:
Thanks for your valuable suggestion. In the revised manuscript, we provided a detailed description of parameters σ_n as well as the perplexity parameter (defaults as 20) as follows:
"…The σ_m in w_mn^+is a sample-specific scaling parameter. It is estimated adaptively by solving a sample-specific root-finding problem, such that the sample-specific distribution over its neighbors having a desired perplexity (see [12] for details). We set a default value of perplexity as 20 in this study." (see Methods on Page 2)
We also pointed out that, "…It is worth to note that, a newly proposed combinational perplexity has been applied to t-SNE [3], which greatly enhances the performance of t-SNE in preservation of global structures. The combinational perplexity can be also adopted by D-EE in future update." (see Methods on Page 2)

Comment:
• page 4, last sentence of "Data description": this is not enough detail. Logarithmic means log(x+1)? How exactly did you find variable features? How many? Please provide full description and cite some literature where you took the recommendations from. If you used a particular software to do it, say so and cite.

Reply:
In the revised manuscript, we described the detailed information on data pre-process as follows
"Firstly, we normalize the gene expression in each sample as follows: we divide each gene read count by the total read counts for each cell, and then multiply a scale factor of 〖10〗^4 and plus one, followed by taking a logarithmic transformation. Secondly, we select the top 2,000 variable genes using the default "vst" method of Seurat package, i.e., variance-stabilizing transformation [21]." (see the second paragraph of Results on Page 4)
The package used to perform pre-process is specified as "The obtained single-cell read count data is pre-processed by Seurat package (version 3.2.1) [19, 20]." (see the second paragraph of Results on Page 4)

Comment:
• page 4: "lambda used is set to the default values" -- what values are default? How can the reader know that? Please specify exact values of all parameters.

Reply:
Thanks for pointing out this issue. In the revised manuscript, we added the default value of parameter lambda as: "In single-cell data analysis, with λ = 10, EE can achieve robust performance with high accuracy [5]. Therefore, we set the default value of λ as 10 for D-EE." (see Methods on Page 2)

Comment:
page 4, description of Figure 2: Frobenius norm only makes sense if you used the same initialization. Please specify in the text that it was the same and also specify what the initialization was (random?).

Reply:
Thanks for your suggestion. In the revised manuscript, we provided the definition of Frobenius norm and

illustrated same initialization was used when perform consistency experiments as:
"… We use the same initialization generated from Gaussian distribution as that in the original EE Matlab code… " (see Results on Page 4)
"…To measure the consistency quantitatively, we calculate the relative error which is defined by relative error $=(\|A-B\|_F)/\|A\|_F$, where $\|\cdot\|_F$ is the Frobenius norm of matrix, A and B represent the output of EE and D-EE, respectively. Frobenius norm of a matrix $A \in R^{(m\times n)}$ is defined as $\|A\|_F=\sqrt{(\sum_{(i=1)}^m \sum_{(j=1)}^n |a_{ij}|^2)}$." (see Results on Page 4)

Comment:
• page 5, "oscillatory patterns" -- I am not sure I see the oscillatory patterns in the figure. I suggest the authors highlight it in the figure somehow.

Reply:
Thank you for pointing out this. In revised manuscript, we added labels and marks on Figure 5 to better illustrate the oscillating pattern.

Close
