# Supplementary material for: D-EE: Distributed software for visualizing intrinsic structure of large-scale single-cell data
Source: Gigascience. 2020 Nov 11;9(11):giaa126. doi: 10.1093/gigascience/giaa126 (PMC7657844; doi:10.1093/gigascience/giaa126)
Supplement: giaa126_Response_to_Reviewer_Comments_Revision_1 [file giaa126_response_to_reviewer_comments_revision_1.pdf]

Author's Response To Reviewer Comments

Close

1) Please include the citation of your new archival GigaDB dataset in the bibliography, including the doi. the citation is:

[xx] An S, Huang J, Wan L Supporting data for "D-EE: a distributed software for visualizing intrinsic structure of large-scale single-cell data" GigaScience Database. 2020. http://dx.doi.org/10.5524/100815

Please cite this dataset form the "availability" section, writing something along the lines: "An archival copy of the code is available via the GigaScience database, GigaDB[xx]".

Reply:
We added our GigaDB dataset in bibliography and cited it in "Availability of supporting data and materials" as suggested in revised manuscript.

2) Please also add the Scicrunch RRID to the "availability" section.

Reply:
We added our RRID number in the "Availability of source code and requirements" section in revised manuscript.

3) Please add ORCIDs of all authors to the title page, if available.

Reply:
We added the ORCIDs of corresponding author L Wan in revised manuscript.

4) In the "availability of supporting data" section, you mention the wishbone github site. Please include this in the bibliography and cite it by number, analogous to the GigaDB example above.
Reply:
We added the GitHub repository in bibliography and cited it in "Availability of supporting data and materials" as suggested in revised manuscript: "The HSPCs data is available in the NCBI repository with accession number GSE72857 and the dataset used in our study is downloaded from their GitHub repository [30]"

Close
