# Supplementary material for: D-EE: Distributed software for visualizing intrinsic structure of large-scale single-cell data
Source: Gigascience. 2020 Nov 11;9(11):giaa126. doi: 10.1093/gigascience/giaa126 (PMC7657844; doi:10.1093/gigascience/giaa126)
Supplement: giaa126_Reviewer_1_Report_Original_Submission — Brian Hie -- 8/18/2020 Reviewed [file giaa126_reviewer_1_report_original_submission.pdf]

Reviewer Report

Title: D-EE: a distributed software for visualizing intrinsic structure of large-scale single-cell data

Version: Original Submission **Date:** 8/18/2020

Reviewer name: Brian Hie

Reviewer Comments to Author:

Does the manuscript adhere to the journal's guidelines on minimum standards of reporting?
Yes
General comments: The authors propose a distributed modification to Elastic Embedding (EE) approaches that provide a multiprocessing speedup to visualization of scRNA-seq data. The manuscript is well written, is based on sound techniques, and describes the overall procedure well. There are some places in which the authors do overclaim or omit important information, so the manuscript would benefit from repositioning the text of the paper accordingly. Overall, I am supportive of publication of the manuscript.
1) Are the methods appropriate to the aims of the study, are they well described, and are necessary controls included?
The math and methodological description of the method is done clearly, which is very much appreciated. Providing GitHub code with instructions for reproducibility is also great.
2) Are the conclusions adequately supported by the data shown?
There are some areas in which the manuscript makes claims that should be toned down. Or, if the authors wish to make these claims, then they would need additional analytic support.
The paper claims that EE "outperforms t-SNE and UMAP" in preserving global structures. Such a claim would need to be supported by a much more exhaustive consideration of different visualization parameters. In particular, for UMAP, I might suspect that with different parameters (e.g., a higher nearest neighbor parameter), the "global structure" would not be broken. Instead, the authors could just say something about how well their own method works without the relative comparison, and still provide single instances of t-SNE and UMAP for a visual comparison.
The authors should also make crystal clear that the speedup is effectively only a constant time one, which may be limiting since implementations of t-SNE (e.g., Barnes-Hut t-SNE or flt-SNE) and UMAP can run in close-to-linear time, which is a big practical win for those methods. In contrast, O(N^2/P) is still large when N = 2 million (e.g., the dataset in Cao et al., Nature, 2019). There's also no speed comparison between distributed EE and, e.g., UMAP. The authors need to make this point clear.
3) Please indicate the quality of language in the manuscript. Does it require a heavy editing for language and clarity?
Language quality is good. One minor nitpick is to avoid contractions, e.g., "it's," in formal writing.
4) Are you able to assess all statistics in the manuscript, including the appropriateness of statistical tests used?
Yes

Level of Interest

Please indicate how interesting you found the manuscript: Choose an item.

Quality of Written English

Please indicate the quality of language in the manuscript: Choose an item.

Declaration of Competing Interests

Please complete a declaration of competing interests, considering the following questions:

- Have you in the past five years received reimbursements, fees, funding, or salary from an organisation that may in any way gain or lose financially from the publication of this manuscript, either now or in the future?
- Do you hold any stocks or shares in an organisation that may in any way gain or lose financially from the publication of this manuscript, either now or in the future?
- Do you hold or are you currently applying for any patents relating to the content of the manuscript?
- Have you received reimbursements, fees, funding, or salary from an organization that holds or has applied for patents relating to the content of the manuscript?
- Do you have any other financial competing interests?
- Do you have any non-financial competing interests in relation to this paper?

If you can answer no to all of the above, write 'I declare that I have no competing interests' below. If your reply is yes to any, please give details below.

I declare that I have no competing interests.

I agree to the open peer review policy of the journal. I understand that my name will be included on my report to the authors and, if the manuscript is accepted for publication, my named report including any attachments I upload will be posted on the website along with the authors' responses. I agree for my report to be made available under an Open Access Creative Commons CC-BY license (http://creativecommons.org/licenses/by/4.0/). I understand that any comments which I do not wish to be included in my named report can be included as confidential comments to the editors, which will not be published.

Choose an item.

To further support our reviewers, we have joined with Publons, where you can gain additional credit to further highlight your hard work (see: https://publons.com/journal/530/gigascience). On publication of this paper, your review will be automatically added to Publons, you can then choose whether or not to claim your Publons credit. I understand this statement.

Yes Choose an item.
