# Supplementary material for: D-EE: Distributed software for visualizing intrinsic structure of large-scale single-cell data
Source: Gigascience. 2020 Nov 11;9(11):giaa126. doi: 10.1093/gigascience/giaa126 (PMC7657844; doi:10.1093/gigascience/giaa126)
Supplement: giaa126_Reviewer_2_Report_Original_Submission — Dmitry Kobak -- 9/15/2020 Reviewed [file giaa126_reviewer_2_report_original_submission.pdf]

Reviewer Report

Title: D-EE: a distributed software for visualizing intrinsic structure of large-scale single-cell data

Version: Original Submission **Date:** 9/15/2020

Reviewer name: Dmitry Kobak

Reviewer Comments to Author:

The paper by An et al. presents a distributed implementation of the elastic embedding (EE) algorithm that allows to run EE on large datasets. The authors argue that EE (and its time-series version TSEE, previously developed by the same authors) is a good choice of an embedding algorithm for single-cell data, and so their implementation allows to actually use it in practice. Previously existing implementations did not scale beyond a sample size of a few thousand cells.

This is a solid work on implementing a parallelized/distributed version of EE. The EE/TSEE algorithms can indeed be an attractive choice of an embedding algorithm for single-cell data, so this paper makes a contribution that is relevant for the field. Importantly, this paper does *not* suggest any new algorithm, but simply presents an efficient implementation of an existing algorithm. If this is in scope of the "Technical note" section of GigaScience, then I can recommend acceptance.

The main issue I have is that it seems the authors used a supercomputer to run their analysis, and it is not clear to me if this implementation would allow to analyze the same dataset (n=250k) on a normal desktop computer. For competitor algorithms like t-SNE and UMAP this dataset size is not a problem even on a laptop. If D-EE can only deal with this dataset on a supercomputer, then it's clearly much much less efficient than competitors. The authors should clarify this point, see also below.

I apologize for taking a long time to write this review (I was away in August).

MAJOR COMMENTS

* Even though the main contribution of the paper is efficient EE implementation, the authors do not provide almost any benchmarking results. What I would like to see reported in the text:

a) What exact hardware was used for the experiments? On page 4 the authors say that they used up to 4000 CPU processes. What kind of computer allows to run 4000 processes? Was it a computing cluster? Please give exact details of the used hardware. Caption of Figure 3 says it was a supercomputer, but more details should be given in the text.

b) What was the actual runtime on this supercomputer? At a minimum, report the physical time for iPSCs dataset in the text (e.g. 1 hour, or 20 hours, or 30 minutes). Even better would be a figure (perhaps another panel in Figure 3) that plots the time as a function of sample size. What is the largest sample size that can be feasibly analyzed on the supercomputer?

c) Most importantly -- most users will not have access to a supercomputer (!!). The text should clearly state how large a dataset can be feasibly analyzed on a typical desktop computer (8 CPU threads, 16 Gb RAM) and perhaps on a typical powerful lab computing station (e.g. 40 CPU threads, 256 Gb RAM). Maybe include the runtimes using such hardware into the figure that I suggested above.

d) In case this implementation only works well on a supercomputer, and is not very helpful on a personal desktop computer, this should be stated very prominently in the abstract/intro/discussion/etc.

Currently the abstract says that D-EE allows "to efficiently" analyze large RNAseq data. But if I must have a supercomputer for such an analysis, then it's not really very "efficient". The abstract should be clear about this. Note that t-SNE and UMAP can easily analyze n=250k on a desktop computer or a laptop. The authors have to discuss this in the Discussion.

* The authors need to give exact details of how they ran t-SNE and UMAP for Figure 4 (page 4). Was it default parameters of some implementation? Name the implementation, give the version, say that it was default parameters. If you changed some parameters from the default ones, specify all of them.

In particular, for t-SNE I am worried that a random initialization was used for Figure 4. Random initialization is a bad idea, as stated e.g. in the cited Ref [8]. UMAP uses non-random init, so this is not a fair comparison. I suggest to use PCA initialization as explained in Ref [8] and also as is default in the latest versions of FIt-SNE and openTSNE.

Apart from that, t-SNE is sensitive to the learning rate (see https://www.nature.com/articles/s41467-019-13056-x), so it should be set appropriately, e.g. to n/12, which is also the default in FIt-SNE and openTSNE.

MINOR COMMENTS

* page 1 -- You might want to consider citing https://www.nature.com/articles/s41467-019-13056-x after "widely used in the single-cell community".
* page 2 -- "EE extends t-SNE", not sure this is a good way to put it. EE is not really an "extension" of t-SNE, is it? It's a different but very related algorithm. Consider revising.
* page 2, a bit later -- "the current implementations ... is" -> are
* page 2, after the first formula: as far as I know, the original EE paper used one fixed value of sigma (kernel width) and not adaptive sigma_n as you do here. I suggest to mention this and explain/cite where the sigma_n comes from. Looks like it's from Ref [11] (last sentence in that paragraph). If so, write that you follow [11] in using adaptive sigma_n based on perplexity (?), and you use perplexity=X by default.
* page 4, last sentence of "Data description": this is not enough detail. Logarithmic means log(x+1)? How exactly did you find variable features? How many? Please provide full description and cite some literature where you took the recomendations from. If you used a particular software to do it, say so and cite.
* page 4: "lambda used is set to the default values" -- what values are default? How can the reader know that? Please specify exact values of all parameters.
* page 4, description of Figure 2: Frobenius norm only makes sense if you used the same initialization. Please specify in the text that it was the same and also specify what the initialization was (random?).
* page 5, "oscillatory patterns" -- I am not sure I see the oscillatory patterns in the figure. I suggest the authors highlight it in the figure somehow.

Level of Interest

Please indicate how interesting you found the manuscript: Choose an item.

Quality of Written English

Please indicate the quality of language in the manuscript: Choose an item.

Declaration of Competing Interests

Please complete a declaration of competing interests, considering the following questions:

- Have you in the past five years received reimbursements, fees, funding, or salary from an organisation that may in any way gain or lose financially from the publication of this manuscript, either now or in the future?
- Do you hold any stocks or shares in an organisation that may in any way gain or lose financially from the publication of this manuscript, either now or in the future?
- Do you hold or are you currently applying for any patents relating to the content of the manuscript?
- Have you received reimbursements, fees, funding, or salary from an organization that holds or has applied for patents relating to the content of the manuscript?
- Do you have any other financial competing interests?
- Do you have any non-financial competing interests in relation to this paper?

If you can answer no to all of the above, write 'I declare that I have no competing interests' below. If your reply is yes to any, please give details below.

I declare that I have no competing interests

I agree to the open peer review policy of the journal. I understand that my name will be included on my report to the authors and, if the manuscript is accepted for publication, my named report including any attachments I upload will be posted on the website along with the authors' responses. I agree for my report to be made available under an Open Access Creative Commons CC-BY license (http://creativecommons.org/licenses/by/4.0/). I understand that any comments which I do not wish to be included in my named report can be included as confidential comments to the editors, which will not be published.

Choose an item.

To further support our reviewers, we have joined with Publons, where you can gain additional credit to further highlight your hard work (see: https://publons.com/journal/530/gigascience). On publication of this paper, your review will be automatically added to Publons, you can then choose whether or not to claim your Publons credit. I understand this statement.

Yes Choose an item.
